# Effects of practical models of low-volume high-intensity interval training on glycemic control and insulin resistance in adults: a systematic review and meta-analysis of randomized controlled studies

**DOI:** 10.3389/fendo.2025.1481200

**Published:** 2025-01-23

**Authors:** Yining Lu, Julien S. Baker, Shanshan Ying, Yichen Lu

**Affiliations:** ^1^ Faculty of Sports Science, Ningbo University, Ningbo, China; ^2^ Centre for Population Health and Medical Informatics, Department of Sport, Physical Education and Health, Hong Kong Baptist University, Hong Kong, Hong Kong SAR, China; ^3^ Department of Sport and Physical Education, Zhejiang Pharmaceutical University, Ningbo, China

**Keywords:** practical model, low-volume, high-intensity interval training, glucose, insulin resistance

## Abstract

**Objectives:**

The aim of this systematic review and meta-analysis was to investigate the effects of practical models of low-volume high-intensity interval training protocols (LV-HIIT) on glucose control and insulin resistance compared with moderate-intensity continuous training (MICT) protocols and no-exercise controls (CON).

**Methods:**

Four databases (PubMed, Web of Science, Scopus, and Cochrane Library) were searched for randomized controlled studies conducted using LV-HIIT interventions (HIIT/SIT protocols involving ≤ 15 min of intense training, within a session lasting ≤ 30 min; < 30 s all-out sprint for SIT additionally). The inclusion criteria required glucose and insulin resistance markers to be evaluated pre- and post-intervention among adults who were not trained athletes.

**Results:**

As a result, twenty studies were included, and meta-analyses were conducted using sixteen studies employing HIIT protocols. Compared with CON, LV-HIIT with reduced intensity and extended interval duration significantly improved fasting glucose (FPG) (mean difference (MD) in mg/dL=-16.63; 95% confidence interval (CI): -25.30 to -7.96; p<0.001) and HbA1c (MD=-0.70; 95% CI: -1.10 to -0.29; p<0.001). Greater improvements were found in participants who were overweight/obese or having type 2 diabetes (T2D). FPG decreased with every additional second of interval duration (β;=-0.10; 95% CI: -0.19 to -0.00; p=0.046). FPI (β;=-0.65; 95% CI: -1.27 to -0.02; p=0.042) and HOMA-IR (β;=-0.22; 95% CI: -0.36 to -0.09; p=0.001) decreased with every additional minute of interval duration per session. HOMA-IR also decreased with every additional minute of weekly interval duration (β;=-0.06; 95%CI: -0.08 to -0.04; p<0.001). Compared with MICT, LV-HIIT was more effective in improving insulin sensitivity (SMD=-0.40; 95%CI: -0.70 to -0.09; p=0.01), but there were no differences in FPG, FPI, HbA1c or HOMA-IR (p>0.05). The effect of LV-HIIT on FPI was larger compared with MICT among individuals who lost weight.

**Conclusion:**

Conclusively, a practical model of LV-HIIT with reduced intensity and extended interval was effective in improving glucose control and its effects were similar to MICT. Greater improvements were found in individuals with overweight/obesity or T2D in protocols with longer intervals or accumulated interval duration per session/week. More large-scale, randomized controlled studies with similar intervention protocols in a wide range of population are warranted to confirm these important results.

**Systematic Review Registration:**

https://www.crd.york.ac.uk/prospero/, identifier CRD42024516594.

## 1 Introduction

The prevalence of type 2 diabetes (T2D) is increasing globally. The latest report (2021) from the International Diabetes Federation (IDF) showed that 1 in 10 adults (537 million) aged 20-79 years are living with diabetes, and among them, more than 90% have T2D. The estimate shows that, by 2045, the prevalence will rise to 1 in 8 adults, consisting of approximately 783 million people. Physical inactivity and obesity are identified as important contributors to the rising prevalence. Although the pathogenic mechanisms are multifarious, insulin resistance seems to play a dominant role. Exercise interventions are the cornerstones for improvement of these conditions. Although the optimal training impulse (volume x intensity x frequency) is unclear, it has remained virtually unchanged in that moderate intensity continuous training (MICT) is the recommended exercise. This includes performing aerobic exercises using a minimum of 1000 kcal/week (1) and walking for a minimum of 2h/week (2). Moreover, a single aerobic activity bout is recommended to last at least 10 min (3). The current physical activity (PA) guidelines highlight that the benefits for reducing the risk of T2D begin to accrue when PA is below the recommended 150-300 min of moderate intensity PA in adults of all body sizes, and additional amounts of moderate- or vigorous-intensity PA appear to reduce the risks even further. Indeed, a previous study showed that total exercise duration played a key role in enhancing insulin action, as more gains in insulin sensitivity could be observed in individuals who exercised more than 170 min per week than those who exercised 115 min per week, regardless of exercise intensity and volume (4). With this in mind, the findings that “lack of time” remains one of most reported exercise barriers is a major concern (3). What is more worrying is that more than 30% of Europeans (5) and 21.6% of Americans (6) fall below the minimum recommended level of PA, and the prevalence of physical inactivity continues to increase (7).

In the last decade, the development of high-intensity interval training (HIIT) offers a time efficient alternative to MICT. HIIT refers to intermittent exercise comprising of short or long bouts of high-intensity exercise interspersed by sufficient or insufficient recovery periods between each bout (8). Compared to MICT, HIIT has been found to induce similar or superior improvements on health-related outcomes, such as cardiorespiratory fitness (CRF) (9) and metabolic health (10, 11), at least in energy matched studies. Although the mechanism by which regular exercise improves glucose regulation and insulin action is not fully understood, it may be related to increased skeletal muscle glycolytic and oxidative capacities following MICT (12), and matched work HIIT has been observed to induce similar acute muscle responses (13). Cochran et al. (14)’s study further indicated that the intermittent nature of stimulus from HIIT is critical for maximizing muscle adaptations in the long term (14). In matched work HIIT programs, the duration of hard efforts was greatly reduced while the total training time was still beyond 30 min. Since low-volume sprint interval training (SIT), a unique form of HIIT, was evidenced to elicit comparable physiological responses and adaptations to MICT in healthy adults (15–18), such low-volume HIIT (LV-HIIT) have started to be used in public health research studies. These LV-HIIT protocols are generally based on the Wingate test, that utilize 4 to 6 bouts of a 30s ‘all-out’ cycling followed by 4 min of recovery.

However, there is still no usable definition of LV-HIIT to date. Sultana et al. (19) defined low-volume as less than 500 MET-min per week, which was approximately equal to 150 min of moderate-intensity physical activity per week recommended by the PA Guidelines. In addition, LV-HIIT was defined as a cumulative interval duration of less than 15 min (20) and a definition with even shorter cumulative interval duration of less than 5 min has been suggested by Yin et al. (21). These reviews have consistently reported positive pooled effects of LV-HIIT on CRF, while effects on cardiometabolic outcomes were controversial (19–21). Moreover, none of the reviews evaluated the effect of LV-HITT on glucose regulation and insulin action.

Babraj et al. (22)’s study seemed to be one of the first studies to explore the use of Wingate-based LV-HIIT to enhance glycemic control in healthy adults. The 30s all-out model was highly effective; nevertheless, it seemed intolerable and unpractical for many untrained individuals (23, 24). Therefore, researchers sought to design a more practical model of LV-HIIT. One of features common to practical LV-HIIT was to reduce exercise intensity, while extending the work bout beyond 30s, with total training time no more than 30min/session. Little et al. (25) was first to examine the efficacy of such a practical model of LV-HIIT in individuals with T2D. Participants completed 6 sessions of LV-HIIT (10 × 60s cycling bouts at 90% maximal heart rate (HRmax), interspersed with 60s rest over 2 weeks and experienced improvements in glucose regulation and skeletal muscle metabolic capacity. Additional methods to make LV-HIIT more practical was to make changes to SIT, to shorten the “all-out” duration to less than 30s, or to reduce the number of “all-out” bouts. These protocols were also termed as reduced-exertion high-intensity interval training (REHIT) (26). For example, Metcalfe et al. (27) employed a REHIT by using only one or two bouts of 10-20s sprints in healthy but sedentary young adults, and an increase in insulin sensitivity was observed following 6 weeks comprising 18 sessions (27). Recently, Sun et al. (28) utilized a LV-HIIT protocol that consisted of 80 repetitions of an extremely short sprint interval of 6s, with 8s rest between each sprint. After a total of 36 sessions over 12 weeks, insulin sensitivity, fasting insulin, and body weight were improved in overweight females (28). However, these findings were derived from small samples, and some studies had no control group. Until now, only two reviews had qualitatively and quantitatively evaluated the effects of HIIT on glucose metabolism and insulin action. Jelleyman et al. (29) concluded that HIIT had positive effects on insulin resistance compared with both MICT and a non-exercising control (CON). While a recent review highlighted that HIIT was superior to a CON but not to MICT (30). However, these reviews were limited as they were not based on randomized controlled studies, nor did they focus on LV-HIIT, making the purported “time efficiency” questionable.

It remains unknown whether practical models of LV-HIIT with a lower exercise intensity or shorter all-out intervals could be effective in improving glucose control and insulin sensitivity in healthy adults as well as those with impaired glucose regulation. It was also unclear whether its effects were different from those using MICT. Addressing these questions objectively using a systematic review and meta-analysis is particularly important because a consensus evidence base is needed to inform public health and provide clinical recommendations for the use of LV-HIIT to mitigate the increasing prevalence of T2D. As such, the primary aim of the current systematic review was to quantify the effects of LV-HIIT on markers of glucose regulation and insulin resistance compared to a MICT or CON using a meta-analysis of randomized controlled studies. A secondary aim was to assess whether observed changes were associated with characteristics of the training protocol, participants’ health status, or concurrent changes in participants’ body mass.

## 2 Method

The present systematic review and meta-analysis was reported according to the Preferred Reporting Items for Systematic Reviews and Meta-Analyses (PRISMA) statement (31), and registered in PROSPERO (CRD42024516594).

### 2.1 Search strategy

Four electronic databases (PubMed, Web of Science, Scopus, and the Cochrane Central Register of Controlled Trials) were searched from the 1^st^ of January 2000 to 31^st^ of December 2023. Medical Subject Headings (MeSH) were used to derive all literature based on the following MeSH terms: “high intensity interval training” AND “glycemic control” OR “glucose metabolism disorders” OR “insulin resistance” and their related terms. In conjunction to MeSH terms, the text words searched were “high intensity training/exercise”, OR “interval/intermittent/sprint training/exercise”, OR “low volume training/exercise”. Randomized controlled studies that reported a measure of glucose regulation (HbA1c, fasting glucose, fasting insulin) or insulin resistance markers assessed pre- and post- intervention were retrieved. Studies were limited to human participants and those published in English. Details of the search strategy are presented in **Supplementary Table S1**. Reference lists of included articles were also examined for any other appropriate studies. All retrieved studies were further manually examined using the pre-determined inclusion and exclusion criteria.

Two authors (Y.L. (Yining Lu) and S.Y.) independently conducted the literature search, quality assessment and data extraction. Disagreements were resolved by discussion and checked by a third reviewer.

### 2.2 Inclusion criteria

#### 2.2.1 Type of participants

Participants included were adults men and women ≥18 years of age, who were not trained athletes and who were not suffering from diseases or conditions that could affect exercise training (e.g. physical and intellectual disability, pregnancy, and lactation). No exclusion criteria were applied to participants’ baseline health status (overweight, obesity, pre-diabetes, diagnosed diabetes were all included); however, studies on participants receiving exogenous insulin therapy or participants with type 1 diabetes were excluded.

#### 2.2.2 Types of intervention

Based on a proposed classification for low-volume interval training, a broad definition of LV-HIIT is used in the current review, involving either HIIT or “sprint interval training (SIT)” (32). Furthermore, HIIT was sometimes referred to as aerobic interval training when the exercise intensity falls within the aerobic capacity of the participants (33). For the purpose of this review, interventions were identified to be HIIT if they were performed with repeated short bouts at high intensities of 77% to 95% HRmax or 64% to 90% VO2max according to the American College of Sports Medicine guidelines (34). SIT interventions were also included if they were performed at “all-out”, “maximal” or “supramaximal” intensities interspersed with recovery time (35).

In the current review, HIIT protocols were considered to be low volume when the intervention involved less than 15 min of intense training (20), within a single session lasting less than 30 min (including warm-up, work-out and cool down). The cut-off of 30 min was chosen because exercise training for 30 min per day for 5 days per week was generally recommended for health (34). In addition, when the intervention was implemented in the form of SIT, the sprint time should be less than 30 s.

MICT was defined as conventional aerobic exercise performed continuously for an extended period (≥ 30 min per session) at a moderate intensity. Moderate intensity was absolutely defined as 3.0 to 5.9 METs or relatively defined as 40% to 59% of oxygen uptake reserve or heart rate reserve according to PA guidelines.

Furthermore, to be included, studies needed to employ an exercise intervention lasting at least 2 weeks, with participants randomly allocated to LV-HIIT, MICT, or CON. Studies involving nutritional supplements were excluded. Studies were also excluded if exercise training was combined with strength/resistance training.

#### 2.2.3 Type of outcome

The outcome measure was glucose control utilizing HbA1c, fasting glucose, fasting insulin, or any measure of insulin resistance/sensitivity.

### 2.3 Data extraction

Data were extracted using a pre-determined form including participant characteristics (age, sex, country), exercise protocol specifics (intervention length, frequency, intensity, work/rest interval, and type), and outcome measures included (markers of glucose and insulin resistance), exercise compliance and adherence.

### 2.4 Study quality and risk of bias

Risk of bias was assessed using the Cochrane collaboration tool (36). Studies were checked for 5 items: random sequence generation, allocation concealment, blinding, description of losses and intention-to-treat analysis. For each item, the risk of bias was judged as “low”, “unclear” or “high”. A score of one point was given for each item classified as “low” and the maximum score was 5 points for each study. The overall quality was categorized as high if all items were low risk of bias.

### 2.5 Statistical analysis

The analyses were performed using Stata V17. LV-HIIT studies employing HIIT or SIT protocols were analyzed separately. Pairwise comparisons were conducted to compare the effect of LV-HIIT on glucose and insulin resistance markers to that of the MICT groups or CON. For studies that included more than one LV-HIIT group, we calculated the pooled effects from all the LV-HIIT groups. Mean difference (MD) was calculated for comparable outcome measures. Standardized mean difference (SMD) were calculated using Hedges’g. The significance level was set at p ≤ 0.05. The effect size based on standardized thresholds was classified as trivial (<0.2), low (0.2-0.6), moderate (0.6-1.2) and high (>1.2) (37). Heterogeneity of included studies was measured and a value >50% was indicative of high heterogeneity. Publication bias was assessed using contour-enhanced funnel plots and the asymmetry was initially evaluated by visual interpretation. Begg and Egger’s asymmetry test was then used for determination when publication bias was apparent. Significant publication bias was considered if p < 0.1. Subgroup analyses were conducted by the BMI category (normal weight vs. overweight/obese), health status (T2D vs. without T2D), and the demonstration of significant reduction on BMI.

Random-effects meta-regression were used to explore the dose-response effects of LV-HIIT on glucose and insulin resistance markers with restricted maximum likelihood estimation when at least 5 studies were eligible. The following variables were selected: (1) the intervention length (week), (2) total number of exercise sessions, (3) interval duration (s), (4) total interval duration per session (min), (5) total interval duration per week (min).

## 3 Results

### 3.1 Study selection

Following the initial search, 5861 potential studies were identified and 3903 were removed as duplicates. After the elimination of 1958 studies based on title and abstract, 97 studies remained for full text screening, and 19 studies met the inclusion criteria. Additionally, we searched for references of included studies, and 1 additional study was considered eligible for inclusion. Finally, a total of 20 studies were included in this review. The study flow diagram is shown in (**Supplementary Figure S1**).

### 3.2 Study quality and risk of bias

Of the 20 RCTs included, 30% were categorized as high quality (6 of 20). The quality score ranged from 0 to 5 and the median quality score was 3.5. 75% presented adequate sequence generation (15 of 20), 70% reported allocation concealment (14 of 20), 65% blinded where possible (13 of 20), 80% reported how many participants failed to follow-up (16 of 20), and only 45% used the intention-to-treat approach for statistical analysis (9 of 20). For details of the study quality and risk of bias see (**Supplementary Table S2**).

### 3.3 Publication bias

Limited publication bias was suggested when visual interpretation was performed. Funnel plots are presented as (**Supplementary Figure S2**).

### 3.4 Study characteristics

There was a total of 738 participants included (416 females and 322 males, mean age: 20-58 years). Eight studies investigated the effects of LV-HIIT against CON, while 8 studies compared the effects of LV-HIIT with MICT. Four studies included both MICT and CON. Sixteen of the 20 studies utilized HIIT protocols, and among them, 2 studies included two different HIIT groups. The remaining 4 studies employed SIT protocols. The intervention length varied from 2 to 16 weeks, with 12 weeks being the most used (n=9). The exercise frequency ranged from 3 to 5 sessions/week.

Other details, including participants’ characteristics, exercise protocols and methods for insulin sensitivity calculation are presented in **Tables 1**, **2**.

**Table 1:** Participant characteristics.

First Author	Group	Country	No.	Age	Sex	BMI	Characteristic
Ahmad et al. (38)	LV-HIIT	Egypt	24	43 ± 6	F	34 ± 3	O/O,T2D
	CON		24	42 ± 6	F	34 ± 3	O/O,T2D
Alvarez et al. (39)	LV-HIIT	Chile	13	46 ± 3	F	31 ± 1	O/O,T2D,SED,N/S
	CON		10	43 ± 2	F	30 ± 0	O/O,T2D,SED,N/S
Fisher et al. (40)	LV-HIIT	US	13	20 ± 2	M	30 ± 3	O/O,T2D,SED,N/S
	MICT		10	20 ± 2	M	29 ± 3	O/O,T2D,SED,N/S
Gallo-Villegas et al. (41)	LV-HIIT	Colombia	29	52 ± 6	21F/8M	30 ± 4	O/O,MetS
	MICT		31	50 ± 6	21F/10M	31 ± 4	O/O,MetS
Koh et al. (42)	LV-HIIT	Denmark	8	56 ± 5	3F/5M	28 ± 3	T2D,N/S
	MICT		8	58 ± 9	4F/4M	29 ± 3	T2D,N/S
Lanzi et al. (43)	LV-HIIT	Switzerland	9	35 ± 3	M	43 ± 1	O/O
	MICT		10	38 ± 2	M	41 ± 1	O/O
Li et al. (44)	LV-HIIT	China	13	38 ± 6	M	27 ± 6	T2D
	MICT		12	39 ± 5	M	27 ± 4	T2D
	CON		12	40 ± 7	M	26 ± 5	T2D
Lu et al. (45)	LV-HIIT	China	59	20 ± 2	F	21 ± 3	SED
	CON		62	20 ± 1	F	21 ± 2	SED
Metcalfe et al. (27)	LV-SIT	UK	15	24 ± 3(F),26 ± 3(M)	8F/7M	23 ± 1(F),24 ± 2(M)	SED
	CON		14	21 ± 1(F),19 ± 1(M)	8F/6M	23 ± 1(F),25 ± 2(M)	SED
RezkAllah and Takla (46)	LV-HIIT	Egypt	20	32 ± 5	9F/11M	28 ± 1	Pre-T2D,O/O,SED
	CON		20	36 ± 6	8F/12M	28 ± 1	Pre-T2D,O/O,SED
Ryan et al. (47)	LV-HIIT	Canada	16	32 ± 7	9F/7M	32 ± 3	O/O,SED,N/S
	MICT		15	30 ± 6	10F/5M	34 ± 3	O/O,SED,N/S
Sabag et al. (48)	LV-HIIT	Australia	12	57 ± 2	5F/7M	38 ± 2	O/O,T2D,SED
	MICT		10	55 ± 2	6F/4M	34 ± 1	O/O,T2D,SED
	CON		10	52 ± 1	4F/6M	36 ± 2	O/O,T2D,SED
Safarimosavi et al. (49)	LV-HIIT	Iran	8	39 ± 5	M	27 ± 3	O/O,Pre-T2D
	MICT-FAT		8	39 ± 4	M	27 ± 3	O/O,Pre-T2D
	MICT-AT		8	40 ± 4	M	27 ± 3	O/O,Pre-T2D
	CON		8	37 ± 3	M	27 ± 2	O/O,Pre-T2D
Shepherd et al. (50)	LV-HIIT	UK	42	42 ± 11	30F/12M	28 ± 5	SED
	MICT		36	43 ± 11	22F/14M	28 ± 5	SED
Sian et al. (51)	HIIT-LAB	UK	10	22 ± 4	6F/4M	25 ± 4	non-Obese
	HIIT-HOME		10	27 ± 4	5F/5M	26 ± 4	non-Obese
	CON		10	24 ± 6	4F/6M	25 ± 4	non-Obese
Skleryk et al. (52)	LV-SIT	Australia	8	40 ± 2	M	32.2 ± 2.1	O/O,SED
	MICT		8	37 ± 1	M	35.2 ± 1.8	O/O,SED
Smith-Ryan et al. (53)	S-HIIT	US	10	37 ± 12	M	32 ± 4	O/O
	L-HIIT		10	41 ± 12	M	28 ± 1	O/O
	CON		5	37 ± 10	M	35 ± 7	O/O
Sun et al. (28)	LV-SIT	China	14	21 ± 1	F	26 ± 3	O/O,SED
	MICT		14	21 ± 1	F	27 ± 2	O/O,SED
Timmons et al. (54)	LV-SIT	Ireland	9	26 ± 4	M	28 ± 2	O/O,SED
	CON		9	25 ± 5	M	27 ± 2	O/O,SED
Winding et al. (55)	LV-HIIT	Denmark	13	54 ± 6	7M/6F	28.1 ± 3.5	T2D
	MICT		12	58 ± 8	7M/5F	27.4 ± 3.1	T2D
	CON		7	57 ± 7	5M/2F	28.0 ± 3.5	T2D

CON, no-exercising control; F, females; L-HIIT, long interval high-intensity interval training; LV-HIIT, low-volume high-intensity interval training; LV-SIT, low-volume sprint interval training; M, males, MICT, moderate intensity continuous training; MICT-AT, moderate intensity continuous training with intensity equivalent to anaerobic threshold; MICT-FAT, moderate intensity continuous training with intensity equivalent to maximal fat oxidation; N/S, non-smoking; O/O, overweight/obesity; S-HIIT, short interval high-intensity interval training; SED, sedentary; T2D, type 2 diabetes.

**Table 2:** Exercise protocols details.

First Author	Group	Week	Frequency (n/week)	Exercise	Intensity required	HR response	Type	Adherence
Ahmad et al. (38)	LV-HIIT	12	3	2×240s,180s	85-90%HRmax	–	Treadmill	–
Alvarez et al. (39)	LV-HIIT	16	3	8-14×30s,120s	90-100%HRmax	–	Jogging/Running	89 ± 5%
Fisher et al. (40)	LV-HIIT	6	3	4×30s,240s	85%HRmax	178 ± 9bpm	Cycling	87%
	MICT	6	5	45-60min	55-65%HRmax	158 ± 11bpm	Cycling	77%
Gallo-Villegas et al. (41)	LV-HIIT	12	3	6×60s,120s	90%VO2max	91 ± 9%,81 ± 7%HRmax	Treadmill	88 ± 11%
	MICT	12	3	30min	60%VO2max	91 ± 9%,81 ± 7%HRmax	Treadmill	85 ± 15%
Koh et al. (42)	LV-HIIT	11	3	10×60s,60s	95%Wpeak	82%HRmax	Cycling	97 ± 13%
	MICT	11	3	40min	50%Wpeak	77%HRmax	Cycling	98 ± 8%
Lanzi et al. (43)	LV-HIIT	2	4	10×60s,60s	90%HRmax	90%HRmax	Cycling	99 ± 1%
	MICT	2	4	40min	67%HRmax	70%HRmax	Cycling	100%
Li et al. (44)	LV-HIIT	12	5	8×60s,60s	80-95%HRmax	–	Cycling	–
	MICT	12	5	30min	50-70%HRmax	–	Cycling	–
Lu et al. (45)	LV-HIIT	12	3	8×20s,10s	80%HRmax	83 ± 2%HRmax	Body-weight	98%
Metcalfe et al. (27)	LV-SIT	6	3	1-2×10-20s	All-out		Cycling	97%
RezkAllah and Takla (46)	LV-HIIT	12	3	10×60s,60s	90%HRmax	–	Treadmill	–
Ryan et al. (47)	LV-HIIT	12	4	10×60s,60s	90%HRmax	–	Multi	95 ± 8%
	MICT	12	4	45min	70%HRmax	–	Multi	95 ± 7%
Sabag et al. (48)	LV-HIIT	12	3	240s	90%VO2max	–	Cycling	98%
	MICT	12	3	40-55min	60%VO2max	–	Cycling	93%
Safarimosavi et al. (49)	LV-HIIT	12	4	10×60s,60s	90%VO2max		Cycling	–
	MICT-FAT	12	4	55min	–		Cycling	–
	MICT-AT	12	4	35min	–		Cycling	–
Shepherd et al. (50)	LV-HIIT	10	3	(4-12)×15-60s,45-120s	90%HRmax	91 ± 6%HRmax	Cycling	83 ± 14%
	MICT	10	5	30-45min	70%HRmax	72 ± 5%HRmax	Cycling	61 ± 15%
Sian et al. (51)	HIIT-LAB	4	3	5×60s,90s	85%HRmax	>85%HRmax	Cycling	100%
	HIIT-HOME	4	3	5×60s,90s	85%HRmax	>85%HRmax	Cycling	90%
Skleryk et al. (52)	LV-SIT	2	3	8-12×10s	All-out	–	Cycling	–
	MICT	2	5	30min	65%VO2max	–	Cycling	–
Smith-Ryan et al. (53)	LV-HIIT-S	3	3	10×60s,60s	90%VO2max	–	Cycling	–
	LV-HIIT-L	3	3	5×120s,60s	80-100%VO2max	–	Cycling	–
Sun et al. (28)	LV-SIT	12	3	80×6s,9s	All-out	82 ± 2%HRmax	Cycling	100%
	MICT	12	3	52-69min	60%VO2max	65 ± 4%HRmax	Cycling	100%
Timmons et al. (54)	LV-SIT	8	3	6×(8×20s,10s),60s	All-out	–	Body-weight	91 ± 7%
Winding et al. (55)	LV-HIIT	11	3	10×60s,60s	95%VO2max	82 ± 4%HRmax	Cycling	91 ± 18%
	MICT	11	3	40min	50%VO2max	75 ± 4%HRmax	Cycling	94 ± 9%

CON, no-exercising control; HR_max_, maximal heart rate; L-HIIT, long interval high-intensity interval training; LV-HIIT, low-volume high-intensity interval training; LV-SIT, low-volume sprint interval training; MICT, moderate intensity continuous training; MICT-AT, moderate intensity continuous training with intensity equivalent to anaerobic threshold; MICT-FAT, moderate intensity continuous training with intensity equivalent to maximal fat oxidation; S-HIIT, short interval high-intensity interval training; VO_2max_, maximal oxygen uptake.

### 3.5 LV-HIIT vs no-exercise CON

#### 3.5.1 Main analysis

**Figure 1:**
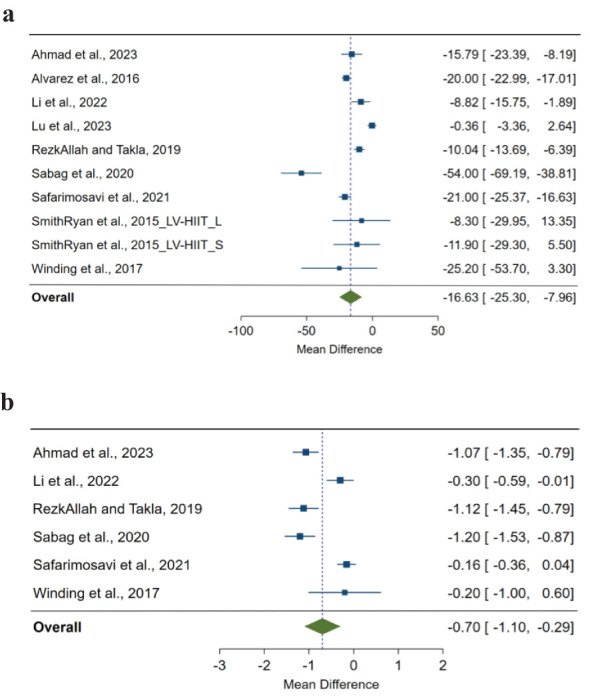
**(A)** The effect of low-volume high-intensity interval training on fasting glucose compared with non-exercising control. The effects are presented as mean difference with 95% confidence interval in mg/dL. A negative value suggests a larger decrease in fasting glucose as a result of low-volume high-intensity interval training compared with a non-exercising control. The overall pooled effect for random effects model represented by green diamond. **(B)** The effect of low-volume high-intensity interval training on HbA1c (%) compared with non-exercising control. The effects are presented as mean difference with 95% confidence interval in the percentage value. A negative value suggests a larger decrease in HbA1c as a result of low-volume high-intensity interval training compared with a non-exercising control. The overall pooled effect for random effects model represented by the green diamond.

We found a significant pooled effect of LV-HIIT, when compared with CON, on FPG (MD=-16.63; 95%CI: -25.30 to -7.96; p<0.001; n=10; **Figure 1A**), and HbA1c (MD=-0.70; 95%CI: -1.10 to -0.29; p<0.001; n=6; **Figure 1B**). Heterogeneity between the studies was substantial for FPG (I^2^ = 95.54%; p<0.001) and HbA1c (I^2^ = 90.07%; p<0.001). We did not find any significant difference on FPI (p>0.05) or HOMA-IR (p>0.05).

#### 3.5.2 Subgroup analyses by BMI category

Among individuals with overweight/obesity, we found significant pooled effects for LV-HIIT on FPG (MD=-18.77; 95%CI: -27.32 to -10.23; p<0.001; n=9; I^2^ = 93.12%) and HOMA-IR (MD=-1.01; 95%CI: -2.03 to 0.00; p=0.05; n=5; I^2^ = 85.66%). The pooled effect on FPI was not significant (p=0.056).

For individuals with normal weight, the pooled effects were not significant on HOMA-IR (p=0.916). The pooled effects on other outcome measures were not calculated due to the insufficient number of studies.

Overweight/Obesity appeared to be a significant moderator for the effect of LV-HIIT on FPG (p<0.001). There were no significant moderation effects for the BMI category on FPI (p=0.058) or HOMA-IR (p=0.052). Details of subgroup analyses are presented in **Table 3**.

**Table 3:** The effect of low-volume high-intensity interval training compared with non-exercising control: subgroup and moderation meta-analyses.

Outcomes	Subgroups	No. Studies	Meta-analyses				Heterogeneity		Moderation effect
			MD	95% CI		p	I^2^	p	p
FPG	BMI category								
	Normal weight	1	-0.36	-3.36	2.64	0.814	–	–	<0.001
	Overweight/Obese	9	-18.77	-27.32	-10.23	<0.001	93.12%	<0.001	
	Healthy Status								
	T2D	7	-20.73	-31.18	-10.28	<0.001	95.73%	<0.001	0.006
	Without T2D	3	-2.84	-10.14	4.45	0.445	33.04%	0.300	
	Significant weight loss after intervention								
	Y	4	-13.25	-24.20	-2.29	0.018	94.51%	<0.001	0.750
	N	2	-30.88	-75.15	13.38	0.171	96.44%	<0.001	
FPI	BMI category								
	Normal weight	1	<0.001	-0.38	0.38	1.000	–	–	0.058
	Overweight/Obese	6	-3.00	-6.09	0.08	0.056	93.03%	<0.001	
	Healthy Status								
	T2D	4	-1.67	-4.31	0.97	0.214	92.78%	<0.001	0.410
	Without T2D	3	-5.80	-13.77	2.17	0.154	75.44%	0.008	
	Significant weight loss after intervention								
	Y	2	<0.001	-0.38	0.38	0.999	<0.001	0.981	0.013
	N	2	-0.25	-0.45	-0.05	0.017	<0.001	0.864	
HbA1c	Significant weight loss after intervention								
	Y	2	-0.72	-1.56	0.12	0.092	75.23%	0.044	0.983
	N	2	-0.75	-1.63	0.14	0.097	93.66%	<0.001	
HOMA-IR	BMI category								
	Normal weight	3	-0.01	-0.09	0.08	0.916	<0.001	0.685	0.052
	Overweight/Obese	5	-1.01	-2.03	0.00	0.050	85.66%	<0.001	
	Healthy Status								
	T2D	3	-0.85	-1.95	0.25	0.131	92.66%	<0.001	0.135
	Without T2D	5	-0.01	-0.09	0.08	0.879	<0.001	0.469	
	Significant weight loss after intervention								
	Y	4	-0.01	-0.09	0.08	0.883	0.00%	0.770	<0.001
	N	1	-0.20	-0.46	0.06	0.124	–	–	

BMI, body mass index; FPG, fasting glucose; FPI, fasting insulin; MD, mean difference; N, no; T2D, type 2 diabetes; Y, yes.

#### 3.5.3 Subgroup analyses by health status

Among participants with T2D, we found significant pooled effects of LV-HIIT on FPG (MD=-20.73; 95%CI: -31.18 to -10.28; p<0.001; n=7; I^2^ = 95.73%). We did not find any significant pooled effects for LV-HIIT on FPI (p=0.214) or HOMA-IR (p=0.131).

As for participants without T2D, there were no significant pooled effects on FPG (p=0.445), FPI (p=0.154), or HOMA-IR (p=0.879). The pooled effects on HbA1c were not calculated because no studies were included.

T2D appeared to be a significant moderator of the effect of LV-HIIT on FPG (p=0.006). We did not find significant moderation effects for T2D on FPI (p=0.410) or HOMA-IR (p=0.135).

#### 3.5.4 Subgroup analyses by the demonstration of significant weight loss after intervention

Among participants who experienced significant weight loss after the intervention, we found a significant pooled effect of LV-HIIT on FPG (MD=-13.25; 95%CI: -24.20 to -2.29; p=0.018; I^2^ = 94.51%; n=4). The pooled effect was not significant on FPI (p=0.999), HbA1c (p=0.092), or HOMA-IR (p=0.883).

Among those who did not lose weight significantly, we found a significant pooled effect on FPI (MD=-0.25; 95%CI: -0.45 to -0.05; p=0.017; I^2^ = 0.00%; n=2). The pooled effects were not significant on FPG (p=0.171), HbA1c (p=0.097), or HOMA-IR (p=0.124).

The demonstration of weight loss post-intervention was a significant moderator of the effect of LV-HIIT on FPI (p=0.013).

#### 3.5.5 Meta-regression

**Figure 2:**
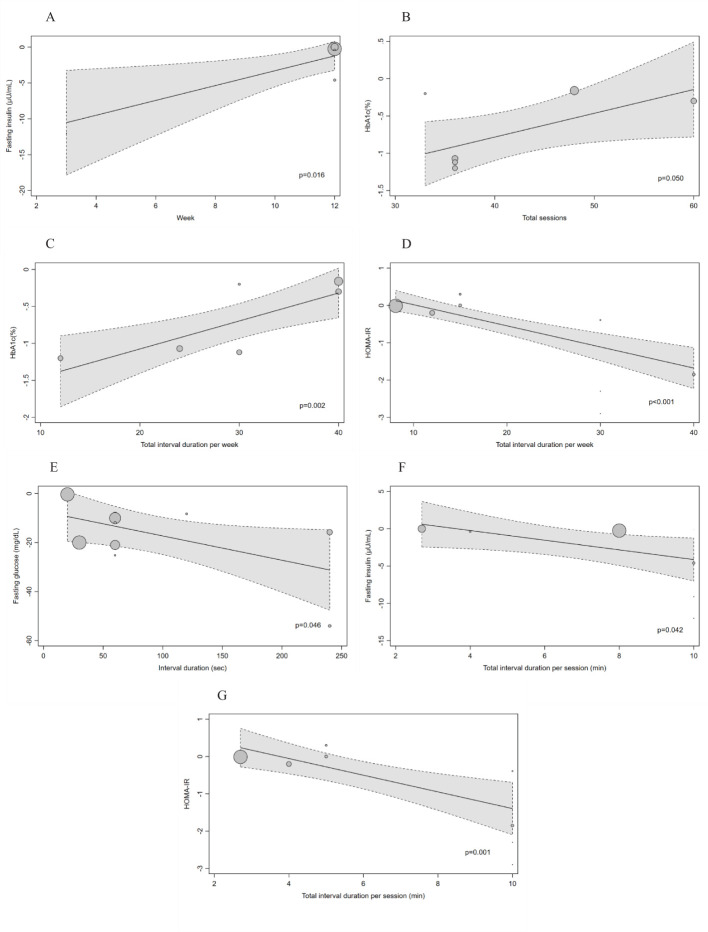
**(A–G)** Dose-response effects of LV-HIIT on FPG, FPI, HbA1c and HOMA-IR: results of meta-regression analysis for variables related to the exercise protocol. The effects are presented as mean difference. The circle sizes are proportional to the effect size of each study. A negative value indicates a larger improvement as a result of low-volume high-intensity interval training compared with a non-exercising control. The dashed line represents the 95% CI of the regression line.

We found a significant dose-response relationship between the intervention length and the effect of LV-HIIT on FPI (β;=1.03; 95%CI: 0.19 to 1.88; p=0.016; **Figure 2A**).

The total number of training sessions was found to be significantly associated with the effect on HbA1c (β;=0.03; 95%CI: 0.00 to 0.06; p=0.05; **Figure 2B**). The effect on HbA1c was also found to be associated with total interval duration per week (β;=0.04; 95%CI: 0.01 to 0.06; p=0.002; **Figure 2C**). It indicated that the more training sessions or the longer interval duration per week, the weaker the effect of LV-HIIT on HbA1c.

Total interval duration per week was also found to be positively related to the effect on HOMA-IR (β;=-0.06; 95%CI: -0.08 to -0.04; p<0.001; **Figure 2D**).

As for the interval duration, we found a significant inverse dose-response relationship with effect on FPG (β;=-0.10; 95%CI: -0.19 to -0.00; p=0.046; **Figure 2E**), showing that longer interval duration had more beneficial effects on FPG. Furthermore, we found significant inverse relationships between the total interval duration per session with the effect on FPI (β;=-0.65; 95%CI: -1.27 to -0.02; p=0.042; **Figure 2F**) and HOMA-IR (β;=-0.22; 95%CI: -0.36 to -0.09; p=0.001; **Figure 2G**). It revealed that the more interval durations per training session, the larger the effect of LV-HIIT on FPI and HOMA-IR.

The complete results of meta-regression analyses are shown in the (**Supplementary Figure S3**).

### 3.6 LV-HIIT vs MICT

#### 3.6.1 Main analysis

**Figure 3:**
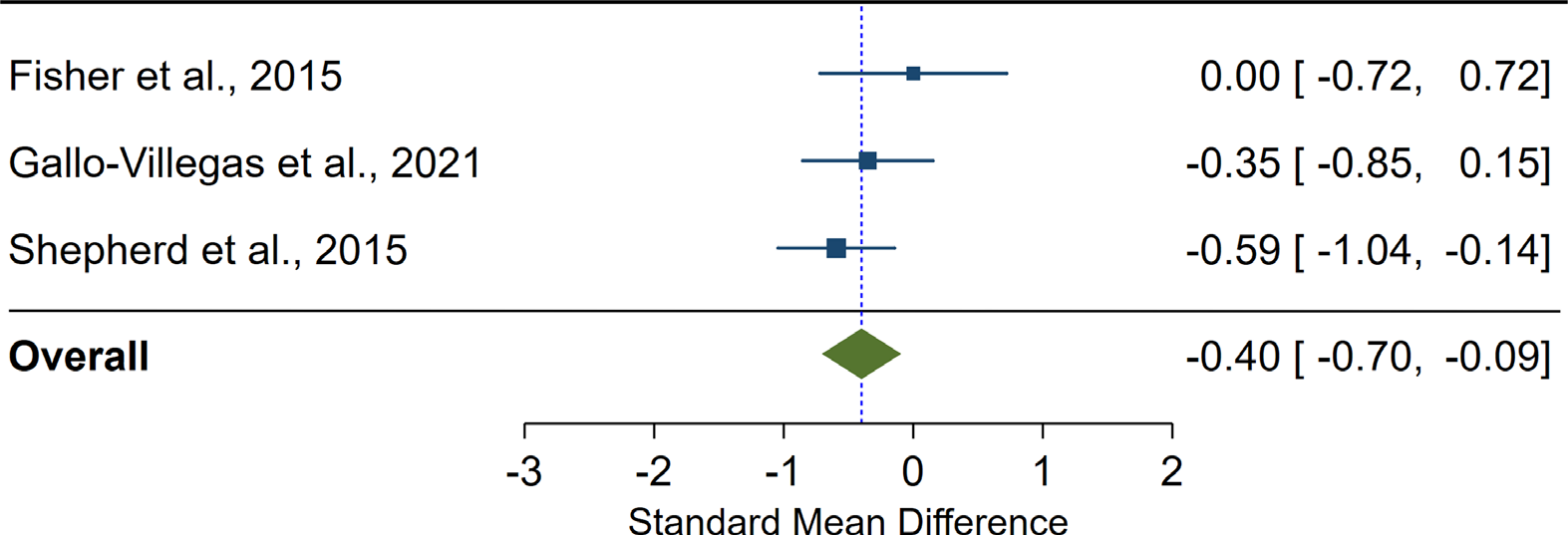
The effect of low-volume high-intensity interval training on insulin sensitivity compared with moderate-intensity continuous training. The effects are presented as standard mean difference with 95% confidence interval. A positive value suggests a larger improvement in insulin sensitivity as a result of low-volume high-intensity interval training compared with moderate-intensity continuous training. The overall pooled effect for the random effects model is represented by the green diamond.

We found a significant pooled effect of LV-HIIT, when compared with MICT on insulin sensitivity (SMD=-0.40; 95%CI: -0.70 to -0.09; p=0.01; n=3; I^2^ = 0.00%; **Figure 3**).

We did not find any significant differences between the effects of LV-HIIT and MICT on FPG, FPI, HbA1c, or HOMA-IR (p>0.05 for all).

#### 3.6.2 Subgroup analyses by BMI category

All the studies included for the calculation of an overall mean difference of FPG, FPI, HbA1c, HOMA-IR and insulin sensitivity were based on participants with overweight/obesity. Details of subgroup analyses were presented in **Table 4**.

**Table 4:** The effect of low-volume high-intensity interval training compared with moderate-intensity continuous training: subgroup and moderation meta-analyses.

Outcomes	Subgroups	No. Studies	Meta-analyses				Heterogeneity		Moderation effect
			MD/ SMD	95% CI		p	I^2^	p	p
FPG	Healthy Status								
	T2D	5	-4.08	-9.89	1.73	0.169	48.40%	0.161	0.256
	Without T2D	4	2.89	-7.66	13.44	0.591	93.43%	<0.001	
	Significant weight loss after intervention								
	Y	2	-3.80	-10.81	3.21	0.288	0.00%	0.817	0.593
	N	4	3.95	-9.72	17.62	0.571	87.67%	<0.001	
FPI	Healthy Status								
	T2D	4	-0.81	-2.03	0.41	0.193	66.06%	0.016	0.432
	Without T2D	3	-2.34	-5.97	1.28	0.205	51.56%	0.127	
	Significant weight loss after intervention								
	Y	2	-1.81	-11.61	7.99	0.717	73.69%	0.051	0.036
	N	2	-0.11	-0.27	0.05	0.193	0.00%	0.361	
HbA1c	Healthy Status								
	T2D	5	-0.05	-0.18	0.09	0.497	26.90%	0.368	0.054
	Without T2D	1	0.20	-0.01	0.41	0.064	–	–	
	Significant weight loss after intervention								
	Y	1	-0.20	-0.83	0.43	0.532	–	–	0.072
	N	2	-0.16	-0.35	0.03	0.102	0.00%	0.588	
HOMA-IR	Healthy Status								
	T2D	5	-0.09	-0.47	0.30	0.657	76.17%	0.006	0.886
	Without T2D	2	-0.05	-0.29	0.18	0.648	0.00%	0.616	
	Significant weight loss after intervention								
	Y	1	-0.21	-0.71	1.13	0.655	–	–	0.790
	N	3	-0.09	-0.29	0.11	0.374	0.00%	0.680	
Insulin sensitivity	Healthy Status								
	T2D	2	-0.24	-0.65	0.18	0.264	0.00%	0.434	0.250
	Without T2D	1	-0.60	-1.05	-0.14	0.010	–	–	
	Significant weight loss after intervention								
	Y	1	-0.59	-1.04	-0.14	0.01	–	–	0.380
	N	1	0.00	-0.72	0.72	0.999	–	–	

BMI, body mass index; FPG, fasting glucose; FPI, fasting insulin; MD, mean difference; N, no; SMD, standard mean difference; T2D, type 2 diabetes; Y, yes.

#### 3.6.3 Subgroup analyses by health status

In participants without T2D, we found that there was a significant mean difference between the effect of LV-HIIT and MICT on insulin sensitivity (SMD=-0.60; 95%CI: -1.05 to -0.14; p=0.01).

For other glucose and insulin markers, including FPG, FPI, HbA1c, or HOMA-IR, we did not find any significant difference between effects of LV-HIIT and MICT in participants with or without T2D.

When compared to MICT, we did not find a significant moderation effect of the effects of LV-HIIT on FPG (p=0.256), FPI (p=0.432), HbA1c (p=0.054), HOMA-IR (p=0.886) or insulin sensitivity (p=0.250) for health status (with T2D vs without T2D).

#### 3.6.4 Subgroup analyses by the demonstration of significant weight loss after intervention

In participants who significantly lost weight following interventions, we found that there was a significant mean difference between the effect of LV-HIIT and MICT on insulin sensitivity (SMD=-0.59; 95%CI: -1.04 to -0.14; p=0.01).

We did not find any significant mean difference between the effect of LV-HIIT and MICT on FPG, FPI, HbA1c or HOMA-IR, in participants with or without significant weight loss after intervention (p>0.05 for all).

When compared to MICT, we found a significant moderation effect of the effects of LV-HIIT on FPI (p=0.036) for the demonstration of weight loss after intervention.

#### 3.6.5 Meta-regression

We did not find any other significant associations between type of training dose and the mean difference between effects of LV-HIIT and MICT on FPG, FPI, HOMA-IR, or HbA1c (p>0.05 for all).

The complete results of meta-regression analyses for LV-HIIT vs. MICT were shown in (**Supplementary Figure S4**).

## 4 Discussion

### 4.1 Main findings

The main findings of this systematic review and meta-analysis were that:

1. LV-HIIT with reduced intensity and extended interval was effective for improving glucose regulation compared to a non-exercising control group, with a mean decrease of 16.63 (7.96 to 25.30) mg/dL in FPG, and a mean decrease of 0.70% (0.29% to 1.1%) in HbA1c. The beneficial effect on FPG was found to be greater among individuals with overweight/obesity or T2D. The demonstration of weight loss after the intervention had moderation effects on FPI and HOMA-IR. Furthermore, a greater effect on FPG could be identified with the LV-HIIT protocol employing longer interval durations, with a further decrease of 1.0 (0-1.9) mg/dl for each additional 10 s. Results from the meta-regression analyses also showed that the longer total interval duration per session was associated with greater effects on FPI and HOMA-IR, with each additional 10 s expected to further decrease FPI by 6.5 (0.2-12.7) μU/mL and decrease HOMA-IR values by 2.2 (0.9-3.6).
2. LV-HIIT was as effective as MICT in improving most glucose and insulin resistance markers. Compared to MICT, a standardized mean decrease of 0.4 (0.09 to 0.7) for insulin sensitivity could be expected when participating in LV-HIIT. It should be noted that these findings were based on participants with overweight/obesity. The demonstration of weight loss after the intervention had a moderation effect on FPI. All components of HIIT protocols defined here did not significantly alter the difference on intervention effectiveness in terms of FPG, FPI, HOMA-IR or HbA1c between LV-HIIT and MICT.

### 4.2 Effects of LV-HIIT on glucose and insulin resistance markers

Results showed that LV-HIIT with reduced intensity and extended intervals was effective in improving glucose control, however FPI and insulin resistance measured by HOMA-IR were not improved. Specifically, the positive effects were categorized as large for both FPG (g=1.69) and HbA1c (g=1.45). These results were in line with recent reviews (56–58). We found the mean reductions in FPG and HbA1c were 15.58mg/dL and 0.75%, respectively. The reductions were comparable to those reported in previous meta-analyses (29, 30, 59, 60). As these studies with comparable findings varied with exercise type and most of them pooled the effects of MICT, HIIT and resistance training, it seemed that LV-HIIT with reduced intensity and extended interval was equally effective in improving glucose regulation despite a reduced time and volume commitment. This notion was supported by recent reviews that evaluated the effectiveness of HIIT protocols with various characteristics on glycemic control (57, 58). It was found that in people with T2D or metabolic syndrome, LV-HIIT was not inferior to higher volume protocols for improving FPG and Hb1Ac (57, 58). However, two LV-HIIT studies that employed SIT protocols did not show any improvements on glucose control or insulin sensitivity (27, 54). Both involved several 10 or 20s maximal exercise bouts. Metcalfe et al. (27)’s study used an extremely low volume protocol, which involved only 1 or 2 bouts per session. After a total of 18 sessions over 6 weeks, a gender-specific result on insulin sensitivity was reported, with improvements in men but not women. This was explained by the fact that women were not able to reach “maximal intensity” quickly at the beginning of a sprint, leading to a greater aerobic contribution involved for this group. In the study by Timmons et al. (54), one training session consisted of six repetitions of 8 bouts of 20 s all-out sprint. Although the training volume was increased compared to that of Metcalfe et al. (27)’s study, FPG was not improved. This was because neither body mass nor fat mass was reduced after training, which was associated with the development of glycemic regulation (61).

In terms of insulin resistance, several studies reported inconsistent results, with the pooled effects ranging from small to large (29, 56, 62). The reason for the inconsistency might be that HOMA-IR was a better measure of hepatic insulin resistance (63), while HIIT was more likely to improve peripheral insulin resistance by increasing the capacity of glucose and fatty acid oxidation in skeletal muscles (64). Therefore, an oral glucose tolerance test might be more suitable to evaluate the effect on peripheral insulin action after HIIT (65). Another potential explanation for our finding might lie in the extremely short exercise duration, since a previous study had suggested that the total exercise duration per week is key to improving insulin action (4). However, this study did not explore the minimum exercise duration to improve insulin sensitivity. In the current review, a minimum exercise duration of 10 min was required to record a significant improvement in HOMA-IR (49). The mechanism by which exercise improved insulin sensitivity requires further studies.

#### 4.2.1 Moderators

Furthermore, our subgroup results supported findings from previous studies suggesting that baseline health status moderates the effects on glucose control (29, 66, 67). Participants with overweight/obesity were more likely to benefit from HIIT (29, 66). In our study, the pooled effects were strengthened in the “overweight/obesity” subgroups when compared to the overall effect, although a few studies were in the “normal weight” subgroup. Similar results were also observed among participants with T2D. This made sense, because T2D and obesity were closely linked, with over 80% of individuals with T2D identified as overweight/obese (68). Although there remained a small percentage of individuals with non-obese diabetes, the pathogenesis of T2D was similar to that of individuals with obese diabetes. It is possible for individuals who were not obese to have excess body fat since “obesity” was commonly defined by BMI, and the excess body fat was associated with insulin resistance, and T2D. Previous studies had delved into the mechanisms by which excess body fat caused T2D. The process started with the increasing secretion of macrophages caused by the hypertrophy of adipocytes, with additional macrophages leading to a pro-inflammatory state. If this condition is not prevented or improved, it can progress to chronic inflammation, resulting in impaired triglyceride deposition and enhanced lipolysis. The excessive circulating triglycerides and free fatty acids may have increased the activated lipid accumulated, inducing a range of metabolic dysfunction including insulin resistance, β-cell dysfunction, prediabetes, and T2D (69, 70).

Thus, the observed positive effects in individuals with obesity or T2D in the present study indicated that LV-HIIT improved body fat or inflammatory status. We did not pool the effect on these two biomarkers because limited studies had reported these outcomes concurrently. Subgroup analyses were conducted among a few studies reporting changes in BMI between the pre- and the post- intervention and results showed that the significant weight loss could not moderate the effects of LV-HIIT. This was consistent with previous reviews (71, 72). While in other studies, weight loss had been identified as a key component in improving insulin resistance (73, 74). This might be the result of limited accuracy of BMI as a measure of body fat (75). Nevertheless, findings from previous reviews showed positive effects of HIIT on waist circumstance, fat mass, percentage body fat and inflammatory markers in individuals with overweight/obesity (21, 66, 76). Another potential explanation may be related to the higher baseline value in this population. Visceral adiposity was found to be associated with elevated FPG regardless of BMI defined obesity (77, 78). This supposition could be supported by the meta-regression findings of a previous study whereby the improvement in glucose control in terms of FPG and HbA1c was not associated with any HIIT characteristics, but rather with the baseline level (29).

#### 4.2.2 Dose-response effects

Our results also showed that longer interval durations in a single bout or accumulated in a session were associated with greater improvements in glucose regulation and insulin resistance. These findings were in agreement with a recent review (57). Our study further presented important first dose-response data. For each additional 10 s of interval duration, FPG was further reduced by 1.0 mg/dL. For each additional 60 s of interval duration in a session, FPI and HOMA-IR were further reduced by 0.65μU/mL and 0.22. Despite the trivial improvements, this indicates that LV-HIIT should preferably be performed with longer intervals to accumulate positive effects on glucose control and insulin resistance. In the current review, the interval duration and the total interval duration per session ranged from 20 to 240 s and 2 to 10 min, respectively. The weekly high intense exercise times ranged from 6 to 40 min. The training intensity was above 85% of HRmax.

Previous studies had demonstrated that in an all-out 30 s sprint, which was generally considered anaerobic, about 50% of the energy contribution was aerobic and muscle glycogen was the major substrate for increased ATP from aerobic pathways (79). With successive bouts or extended exercise duration, there was an expanded contribution from oxidative phosphorylation (80), resulting in a substantially high muscle glycogen aerobic metabolism (81). Although these findings were from SIT protocols, which were Wingate based, it was suggested that the initial intensity bout worked as a starting point, allowing subsequent bouts to stimulate glucose aerobic metabolism more effectively. From the perspective of site specificity of exercise training, improvement in skeletal muscle oxidative capacity following LV-HIIT had been reported in several studies (82, 83), potentially through increases in mitochondrial capacity and GLUT4 protein content (25). Similar skeletal muscle adaptations were observed in original Wingate-based LV-HIIT (15, 16). Furthermore, it was also apparent from work by McCartney et al. (84) that with successive 30 s sprints, there was greater contribution of lipolysis due to inhibition of glycogen degradation and inability to resupply PCr maximally due to the relatively brief recovery duration. Therefore, skeletal muscle adaptations following LV-HIIT might involve fat oxidation, which had been identified as a predictor of glycemic control. Although less well documented, findings from recent reviews showed that interval training in the form of HIIT or SIT can elicit increases in fat oxidation, with HIIT more likely to increase fat oxidation than SIT (85, 86). In addition to skeletal muscle adaptations, intense exercise might stimulate hepatic glucose production because of increases in catecholamine and glucagon levels in response to LV-HIIT (87), and enhanced hepatic insulin sensitivity. In Terada et al. (87)’s study, the LV-HIIT comprised of 15 bouts of 1 min high intensity exercise interspersed by 3 min active recovery.

Therefore, the pathway by which LV-HIIT with extended duration and reduced intensity improves glucose regulation and insulin resistance may be the result of enhanced skeletal muscle glycogen and fat oxidation capacity and liver glycogen metabolism.

Accordingly, even with a reduced training volume, LV-HIIT should be performed at a longer interval to maximize the stimulation of glucose and fat metabolism. However, there was still no consensus on the optimal interval. Perhaps there was no point in discussing “optimal”, what seems to be important was the rapid depletion of glycogen through intense exercise to effectively mobilize glucose and fat metabolism in successive high-intensity bouts.

### 4.3 Differences between effects of LV-HIIT and MICT on glucose and insulin resistance markers

Results showed that LV-HIIT protocols with reduced intensity and extended interval duration were more effective than MICT in improving insulin sensitivity, and were equally as effective in improving FPG, FPI, HbA1c and HOMA-IR. Our findings advanced a recent review which demonstrated that HIIT improved glucose regulation similarly in adults with T2D when compared with MICT (57). That study, however, did not find any further benefits from LV-HIIT, whereas our study observed that LV-HIIT was superior to MICT in reducing FPI and insulin sensitivity. This discrepancy might be due to the more strict definition of LV-HIIT used in Opazo−Díaz et al’s study, as HIIT was considered to be low volume when the session involved less than 5 min of training as compared with our 15 min. Despite the small effect size (g=0.40 for insulin sensitivity), this could be considered practically important. In the studies included in the meta-analysis, the average exercise time of the LV-HIIT was only a quarter of that of the MICT.

Furthermore, although a meta-analysis was not performed, one of LV-HIIT studies using SIT protocol reported improvements in insulin resistance after training (28) while the other study reported no gains (52). These contradictory results might be explained by substantial differences in the training protocol used in terms of training length, interval duration, and number of repeats. In the work by Skleryk et al. (52), participants completed 2 weeks of LV-HIIT, which involved a total of 6 sessions of 8-12 repeated 10 s all-out cycling. It differed from previous studies that had reported improvements in insulin sensitivity using the 30 s all-out model (22, 88). The authors explained that on the one hand, SIT-induced improvements on insulin action were less pronounced in individuals with obesity compared with those with normal weight, and on the other hand, 10 s interval duration was too short to stimulate substantial short-term glucose uptake. Sun et al. (28) employed an even shorter interval of 6 s, and participants and measurement timepoints were both similar to Skleryk et al. (52). In Sun et al. (28)’s study, after performing 80 repetitions per session over 12 weeks, participants in the SIT group experienced significant improvements in insulin sensitivity, while those in the MICT group had no gains. It seemed that for “all-out” models, total interval duration and training length might be important determinants for positive adaptations.

Conversely, a previous review found that HIIT was less effective than MICT in improving insulin sensitivity (89). The authors explained that MICT enhanced fatty acid metabolism in skeletal muscle, reduced lipid accumulation, and induced a direct improvement in insulin sensitivity (90). It was noteworthy that all participants in McGarrah et al.’s study were overweight or obese, and the result was based on energy matched studies, which was not the case in our reviewed studies. The underlying mechanism for improving glucose metabolism and insulin resistance through a certain amount of energy expenditure was consistent with the basis on which current PA guidelines were developed. One of the mechanisms by which MICT was recommended was that GLUT4 concentration increased after MICT. The increase in skeletal muscle GLUT4 content was linked to an enhanced capacity for insulin-stimulated glucose uptake, and thus was a key factor regulating insulin sensitivity (91). However, comparable increases in GLUT4 had been reported after low-volume sprint interval training (16, 17), as well as after LV-HIIT as defined in the current review (92). Although there were no comparisons, other LV-HIIT studies have reported training induced increases in GLUT4 (25, 93). This suggests that there might be another mechanism existing in the LV-HIIT as both training volume and duration were largely reduced. As we discussed above, during LV-HIIT, glucose was rapidly depleted at the beginning of exercise, when insulin was temporarily suppressed, and instead the rapid muscle contraction led to the translocation of GLUT4 (94). Simultaneously, whole-body glucose metabolism was mobilized and the delivery rate of glucose to exercising muscle was largely increased to meet the high energy demands during intense exercises. It was unique in terms of HIIT that the rate of glucose production was greater than that of utilization, resulting in an increase of glycemia and the exhaustion of plasma insulin. During the recovery period after intense exercise, it took about 40-60 min to restore plasma glucose through substantial secretion of insulin (95, 96). From this point of view, the effect of LV-HIIT on glucose regulation and insulin resistance might, in part, be caused by rapid muscle contraction during exercise and highly activated insulin action post exercise. Although several studies had found differential metabolic responses involved between MICT and HIIT, the mechanisms by which they improved glucose regulation and insulin action were not fully understood.

Nevertheless, given that the lack of time was one of the most cited barriers to exercises (97), our findings suggest that, for most individuals, LV-HIIT seemed to be a pragmatic way accompanied by feasibility and accessibility to gain health benefits via exercises.

#### 4.3.1 Moderators

Our results showed that LV-HIIT was as effective as MICT in improving glucose and insulin resistance markers, regardless of whether the participants were overweight/obese, had T2D, and experienced significant weight loss after the intervention. This agrees with previous studies (30, 98). The findings indicated that LV-HIIT could be used as an alternative when prescribing exercise to individuals who were overweight/obese and have T2D, particularly among those who have exercise time restrictions, priority should be given to LV-HIIT.

#### 4.3.2 Dose-response effects

Previous studies have found that interventions with longer durations favorably influenced the effect of HIIT on fat mass and CRF compared to MICT (99, 100). However, in the current review, the differences on training effects between LV-HIIT and MICT were not associated with any components of the HIIT protocol, including training length, total sessions, interval duration and time spent at high intensity in a session. This was in agreement with recent reviews (57, 58). The longest training duration in the current review was 16 weeks, which might not be long enough to see clinical changes and sustainability in physiological outcomes. To clearly investigate the difference in training effects on glucose control and insulin resistance between LV-HIIT and MICT, long-term trials of high methodological quality are warranted.

#### 4.3.3 Practical implications

LV-HIIT was effective in the primary, secondary, and tertiary prevention of T2D in adults. Individuals could expect to achieve a large improvement in glucose regulation by engaging in LV-HIIT. Greater improvements would be expected in individuals with overweight/obesity or T2D. When prescribing LV-HIIT protocols, those with longer interval duration and longer interval duration accumulated in a session would gain more benefits for glucose regulation and insulin resistance markers.

When recommending exercises to improve glucose regulation and insulin resistance, LV-HIIT should have the same priority as MICT in terms of the intervention effectiveness. It should also be highlighted that, in terms of training efficiency, LV-HIIT would be preferable than MICT as it took only a quarter of the exercise time to achieve similar improvements. The reduced time and volume commitment might be of great importance for most individuals.

Furthermore, it was crucial to acknowledge that the intense nature of LV-HIIT might be intolerable and unpractical for some untrained individuals. Such intense exercise requires high levels of participant motivation and negative affective responses were previously reported (23, 24). These negative responses include exercise exertion, unpleasant, maladaptive, or even noxious experiences, resulting in attenuated exercise fidelity and maintenance (24). Therefore, it was important to choose the appropriate LV-HIIT protocol for different populations. In addition, affective responses should be evaluated concurrently to evaluate the feasibility of LV-HIIT in long-term health promotion strategies.

#### 4.3.4 Limitations

There were several limitations that need consideration. Firstly, different LV-HIIT and MICT protocols were used in the studies included in the meta-analyses, and different population groups were also included. These contributed to the high heterogeneity between studies, making the results needing caution during interpretation. Secondly, we should acknowledge that a relatively small number of studies were included in some of the subgroup analyses. For example, most studies were conducted in participants with overweight/obesity, leading to limited data in normal weight individuals. In fact, it was equally important and meaningful to investigate the “normal weight” group. Since the BMI thresholds were widely used to define being overweight and obesity, this led to individuals with normal weight obesity being largely ignored. Individuals with “normal weight obesity” were also associated with insulin resistance (101, 102), and they were at an increased risk of developing T2D (103, 104). Moreover, moderation effects were assessed independently without considering any potential interactions. Thus, results of subgroup and moderator analyses should be interpreted with caution. Thirdly, because no studies had proposed dose-response relationships between changes in glucose and insulin markers and health outcomes, it was difficult to interpret our results in the clinical sense. The utilization of the general classification of effect size for intervention studies might not be valid. Furthermore, the mean age of participants in the current study was relatively young, which makes the effects of LV-HIIT on older adults unclear. Finally, only a small number of studies reported exercise compliance. This is an important metric for the successful delivery of LV-HIIT while a more recent study claimed that participants in the HIIT were more likely to exercise at lower-than-prescribed intensities (105). The average heart rate during exercise was the most common measure for exercise compliance. However, for HIIT, there was a tremendous lag of heart rate responses from the beginning of exercise. The mean heart rate or the time spent at higher intensities, which were the important mediators of the adaptive responses to HIIT need further investigations.

#### 4.3.5 Future directions

Several directions for future research are proposed. As the majority of participants included in our meta-analyses were young adults, future research should clarify the effects of LV-HIIT in children, adolescents, and older adults. The moderating effects of weight loss and body fat on insulin resistance were still unclear. More research is warranted to investigate these factors. Furthermore, more evidence on long-term health benefits from the large-scale, long-term randomized clinical trials are needed to confidently inform public health and physical activity guidelines on LV-HIIT. In addition, future intervention studies should provide information regarding exercise compliance, more detailed participant information such as race/ethnicity, co-morbidity and co-behavioral information. Finally, most of LV-HIIT protocols in our meta-analyses were performed in an equipment-based manner, such as cycling on the cycle ergometer and running on treadmills. A few studies used bodyweight-based exercises. Investigating the effectiveness of non-equipment-dependent LV-HIIT are of great importance in exercise adherence and compliance and require further investigation.

## 5 Conclusion

Our systematic review and meta-analysis showed that LV-HIIT was effective for improving glucose regulation, and the effects were comparable to that of MICT. Greater improvements were observed in participants with overweight/obesity or T2D. We also found that the prolonged LV-HIIT protocol and that employed longer interval durations and interval durations accumulated per session were associated with greater benefits. More high quality RCTs with similar protocols in a wide range of populations are needed to improve the certainty of the evidence on the effects of LV-HIIT on glucose regulation and insulin resistance. The findings observed here have important implications in the prescription of exercise for improving glucose regulation.

## Data Availability

The original contributions presented in the study are included in the article/**Supplementary Material**. Further inquiries can be directed to the corresponding author.
