# Supplementary material for: Effects of practical models of low-volume high-intensity interval training on glycemic control and insulin resistance in adults: a systematic review and meta-analysis of randomized controlled studies
Source: Front Endocrinol (Lausanne). 2025 Jan 23;16:1481200. doi: 10.3389/fendo.2025.1481200 (PMC11798773; doi:10.3389/fendo.2025.1481200)
Supplement: Supplementary file 1 [file DataSheet1.docx]

**Supplementary Figure S1: Study flow diagram**


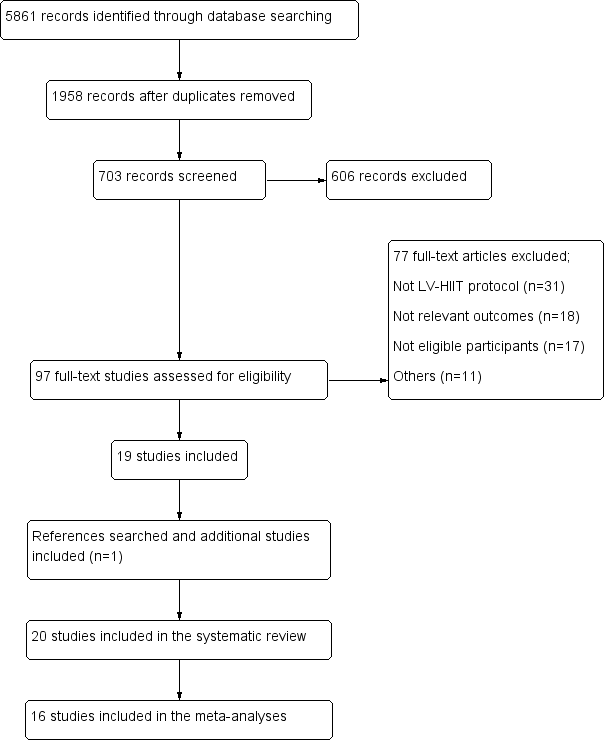


**Supplementary Figure S2: Publication bias**

Fasting glucose LV-HIIT vs. CON


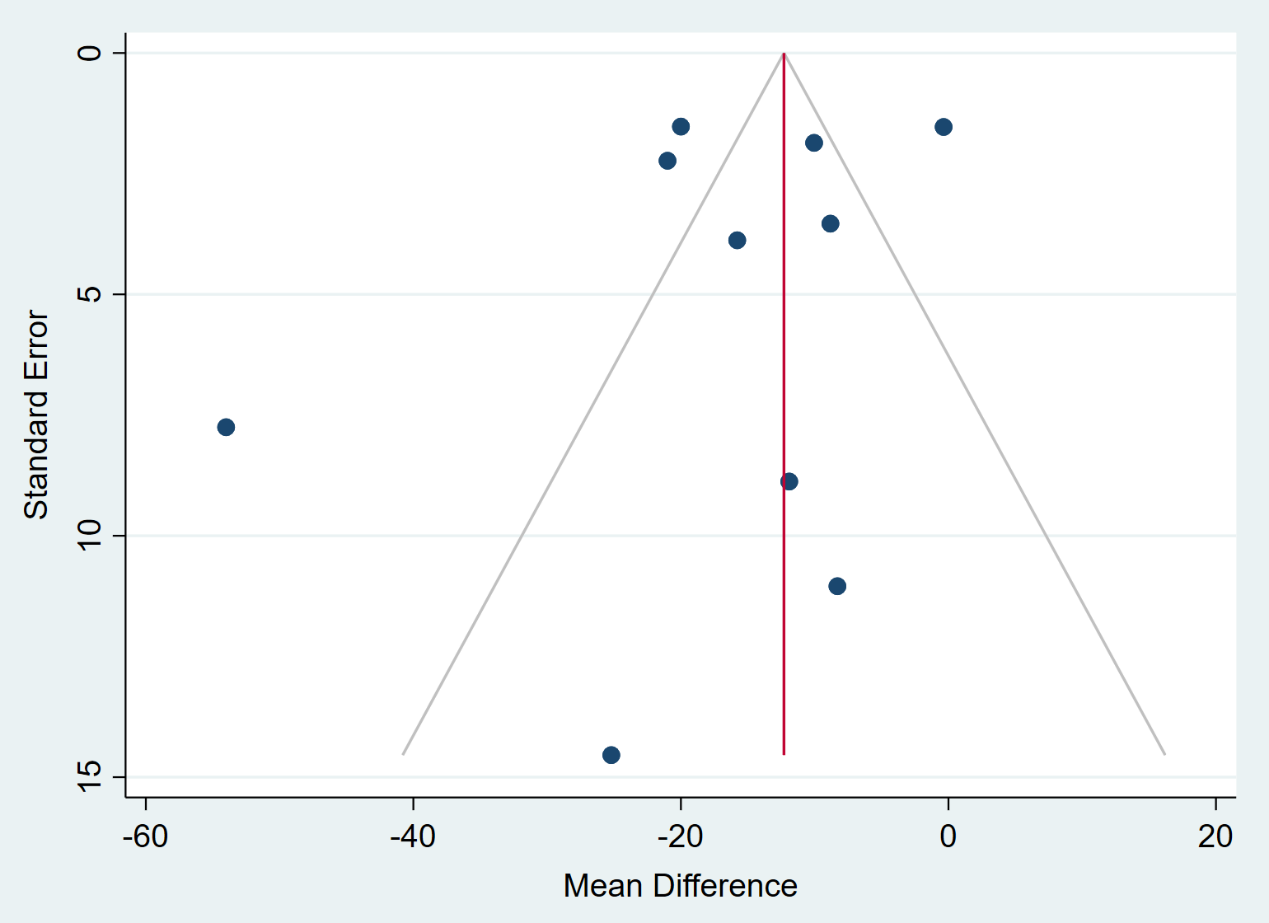


Fasting glucose LV-HIIT vs. MICT


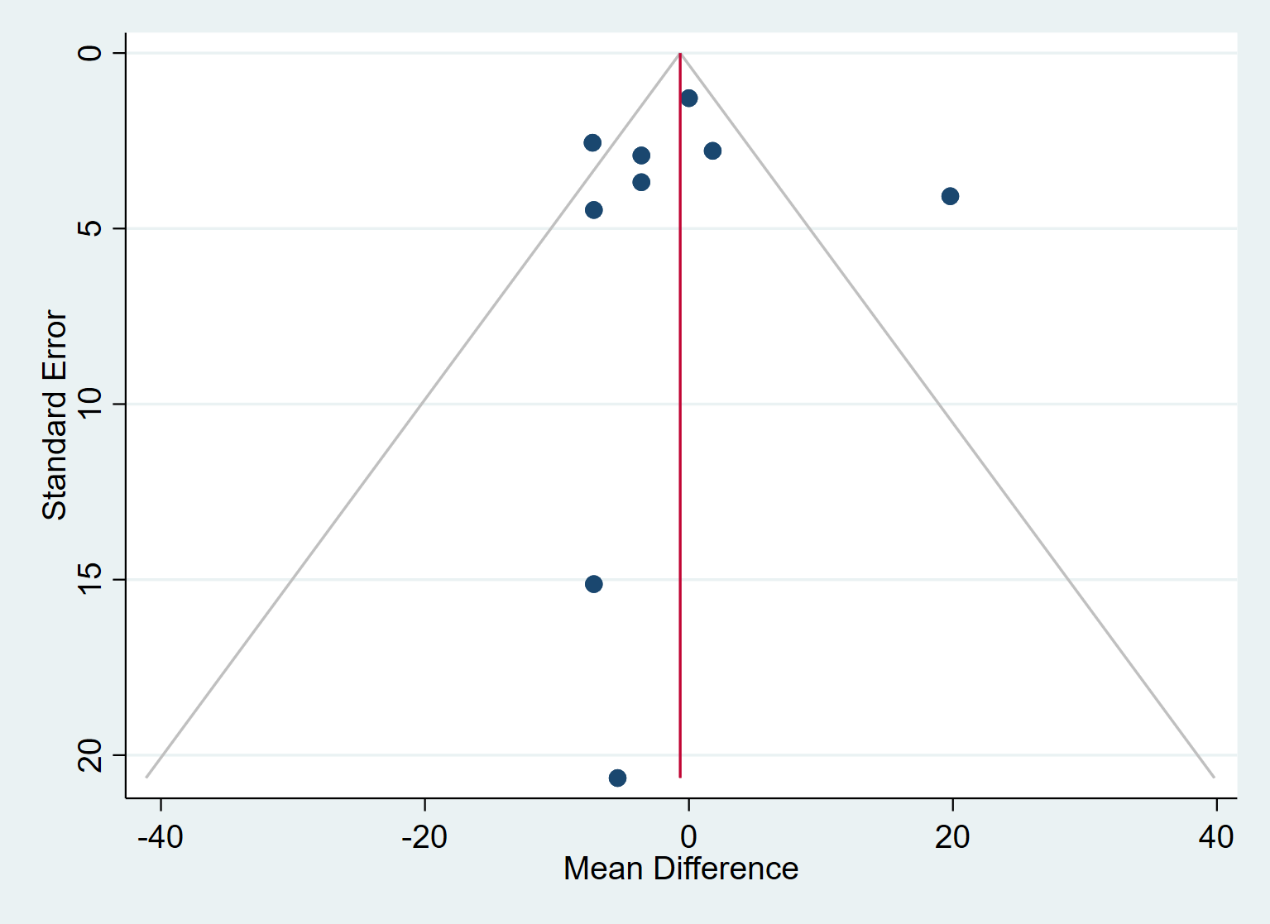


Fasting insulin LV-HIIT vs. CON


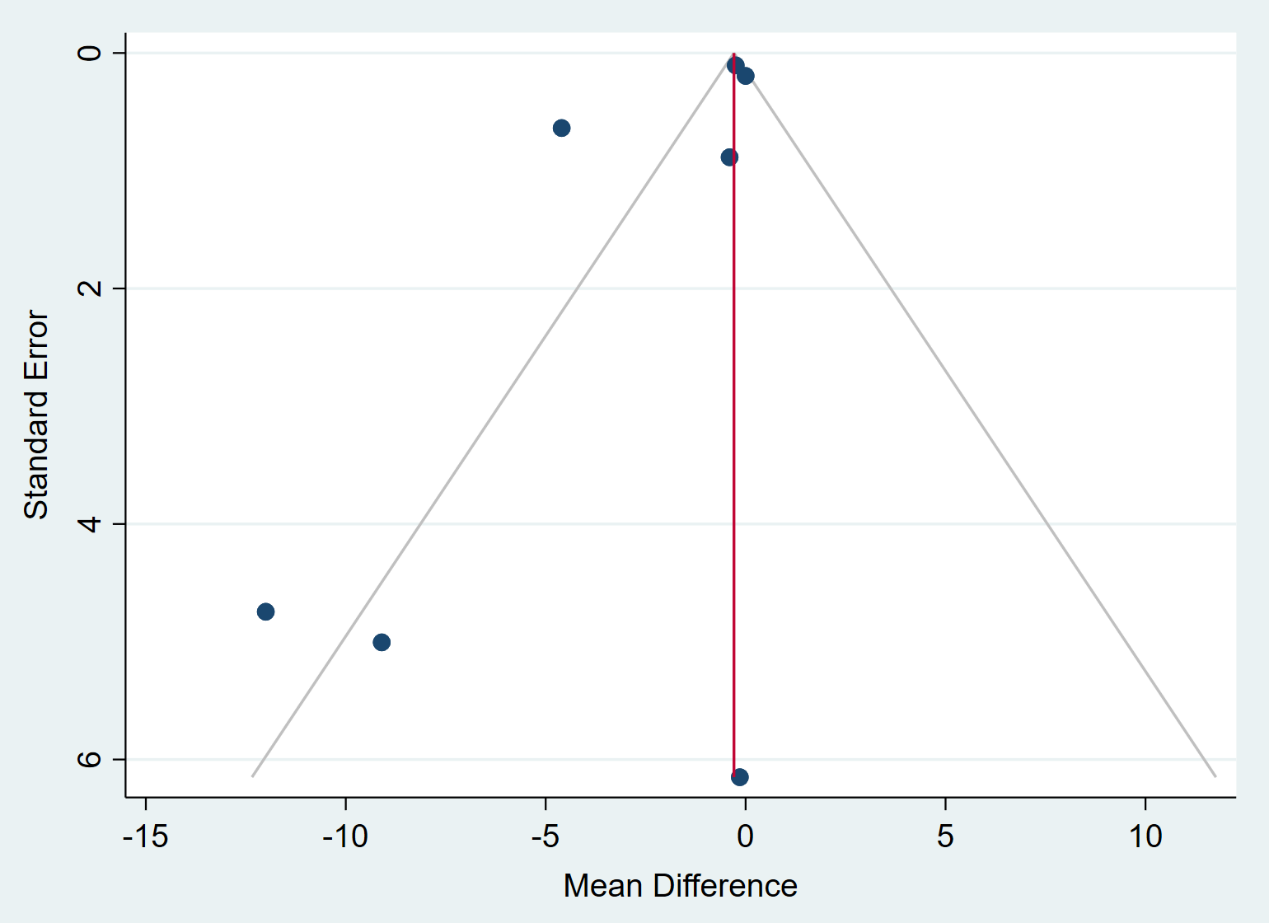


Fasting insulin LV-HIIT vs. MICT


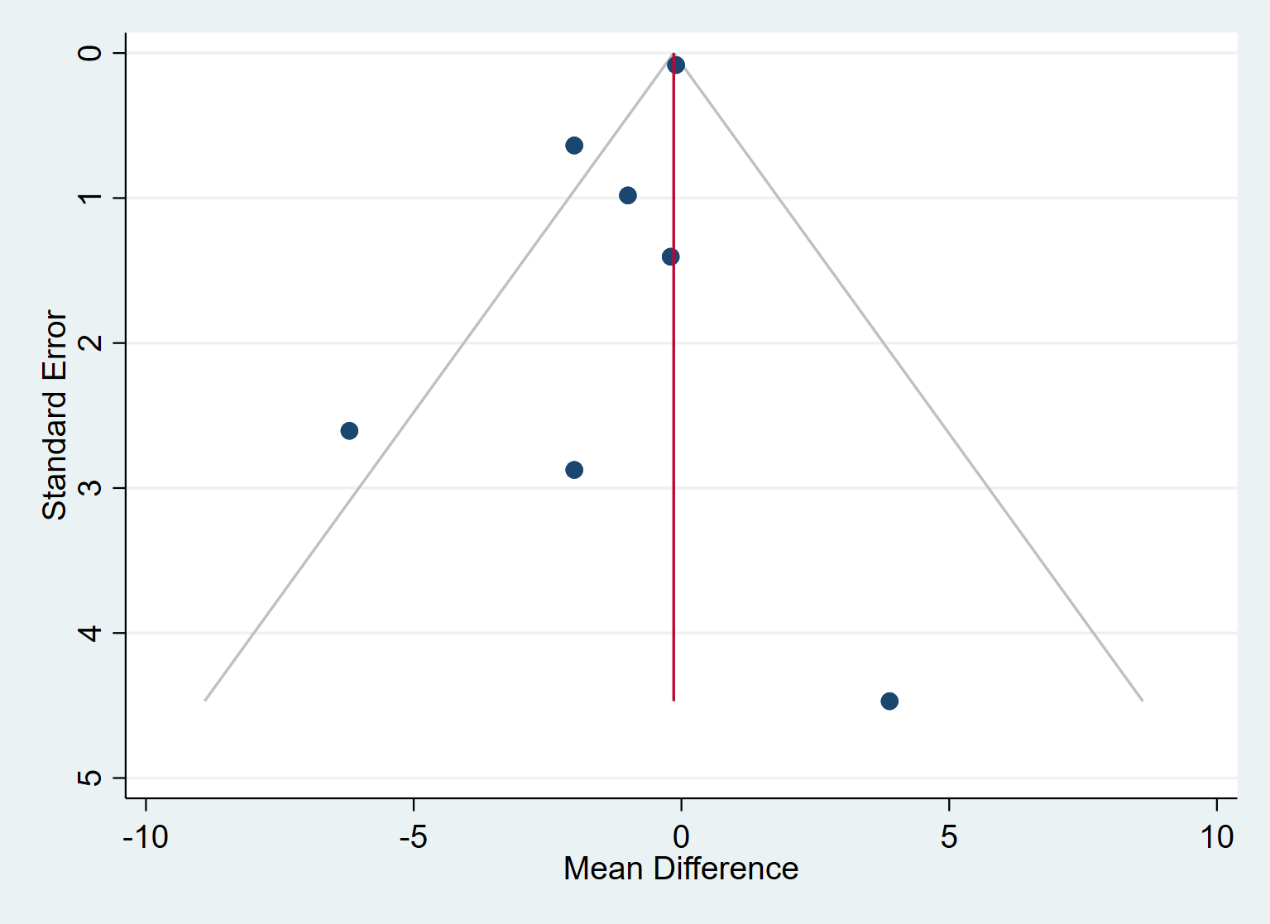


HbA1c(%) LV-HIIT vs. CON


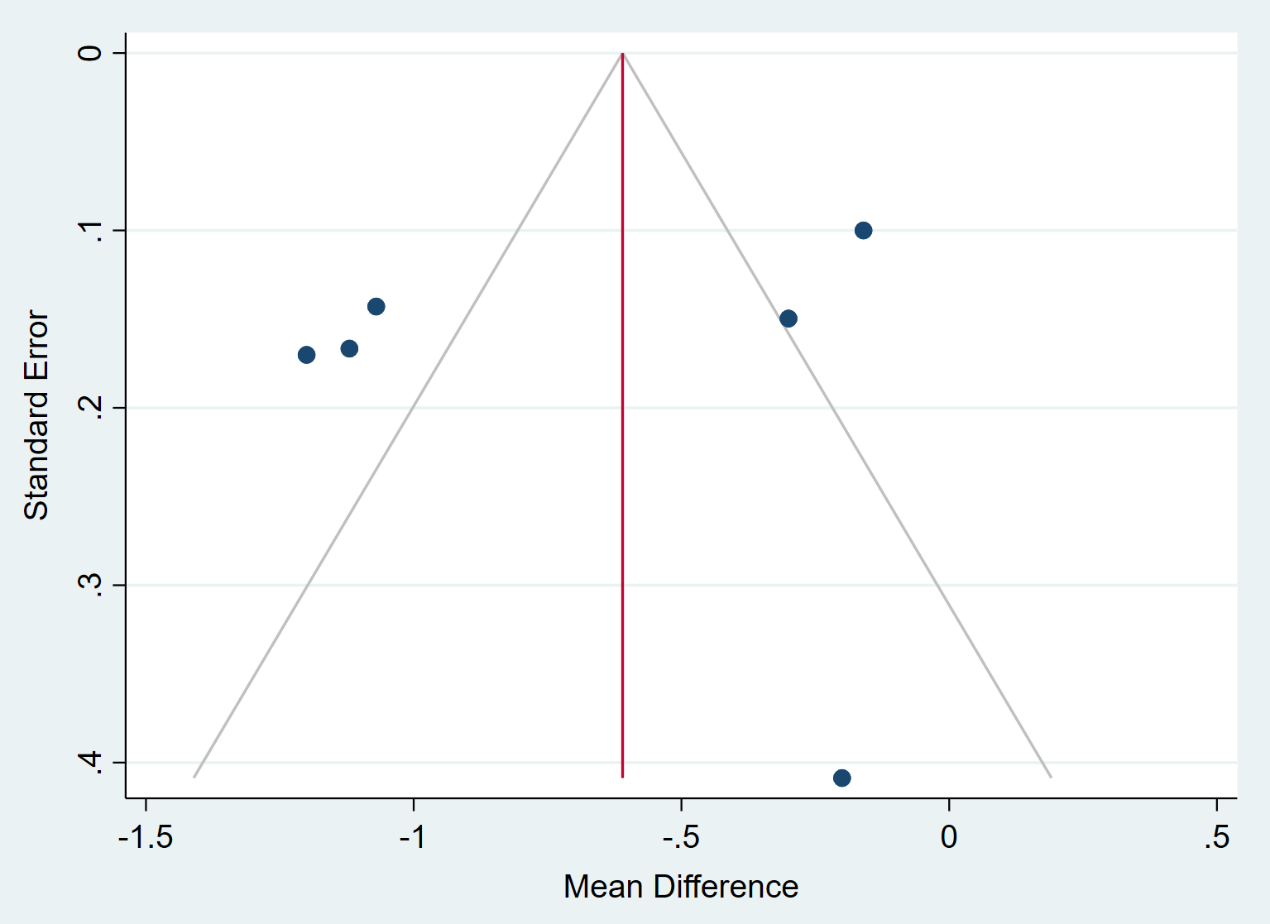


HbA1c(%) LV-HIIT vs. MICT


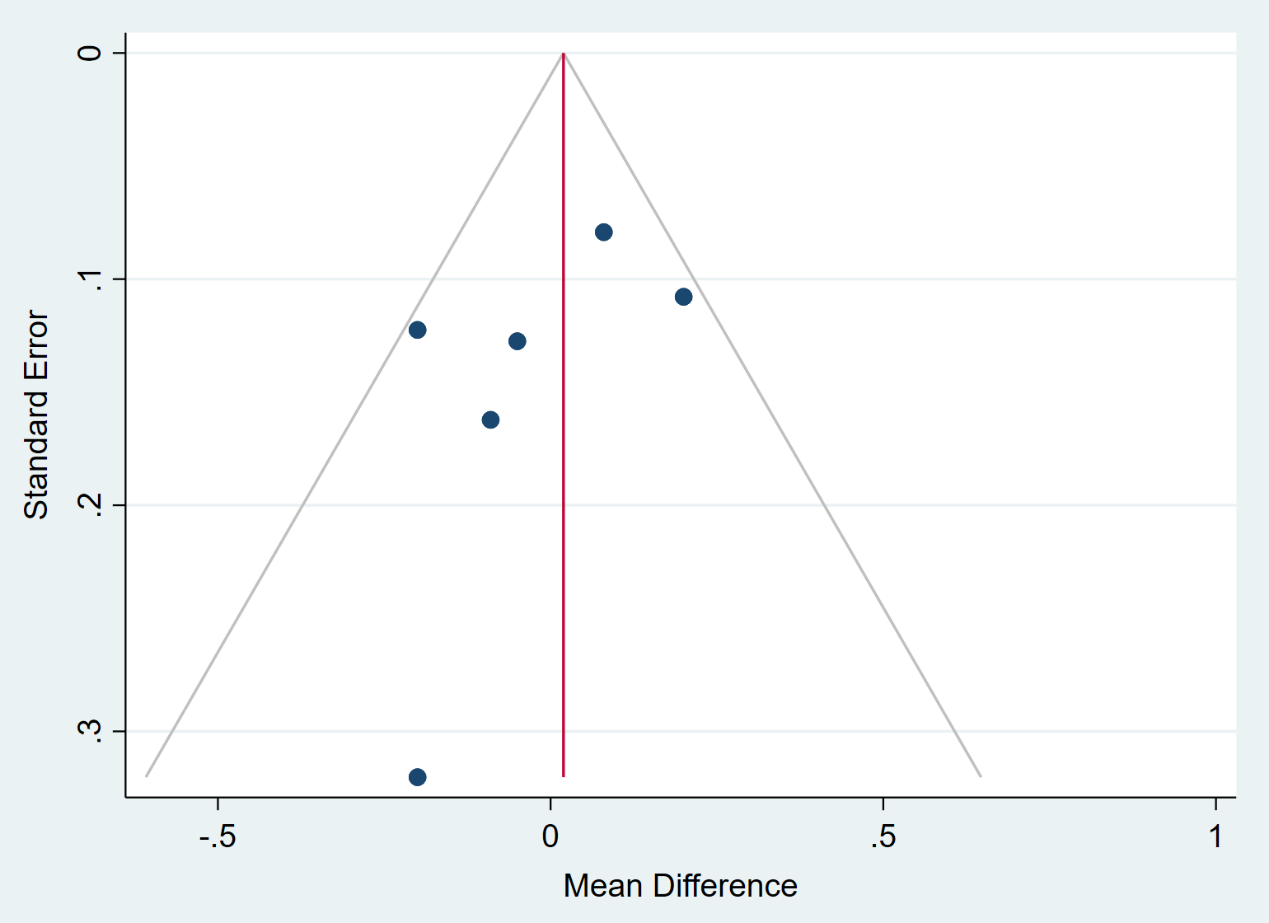


HOMA-IR LV-HIIT vs. CON


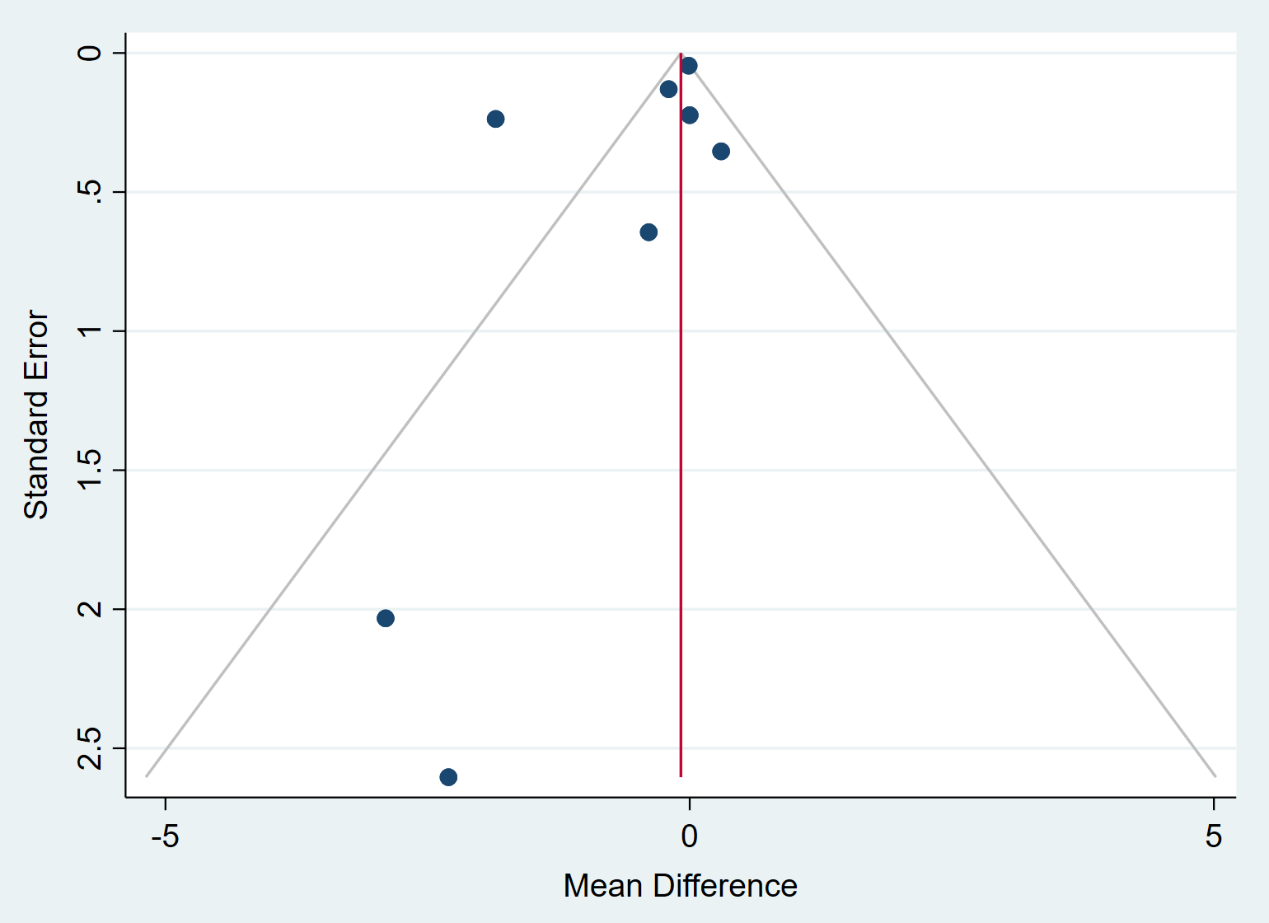


HOMA-IR LV-HIIT vs. MICT


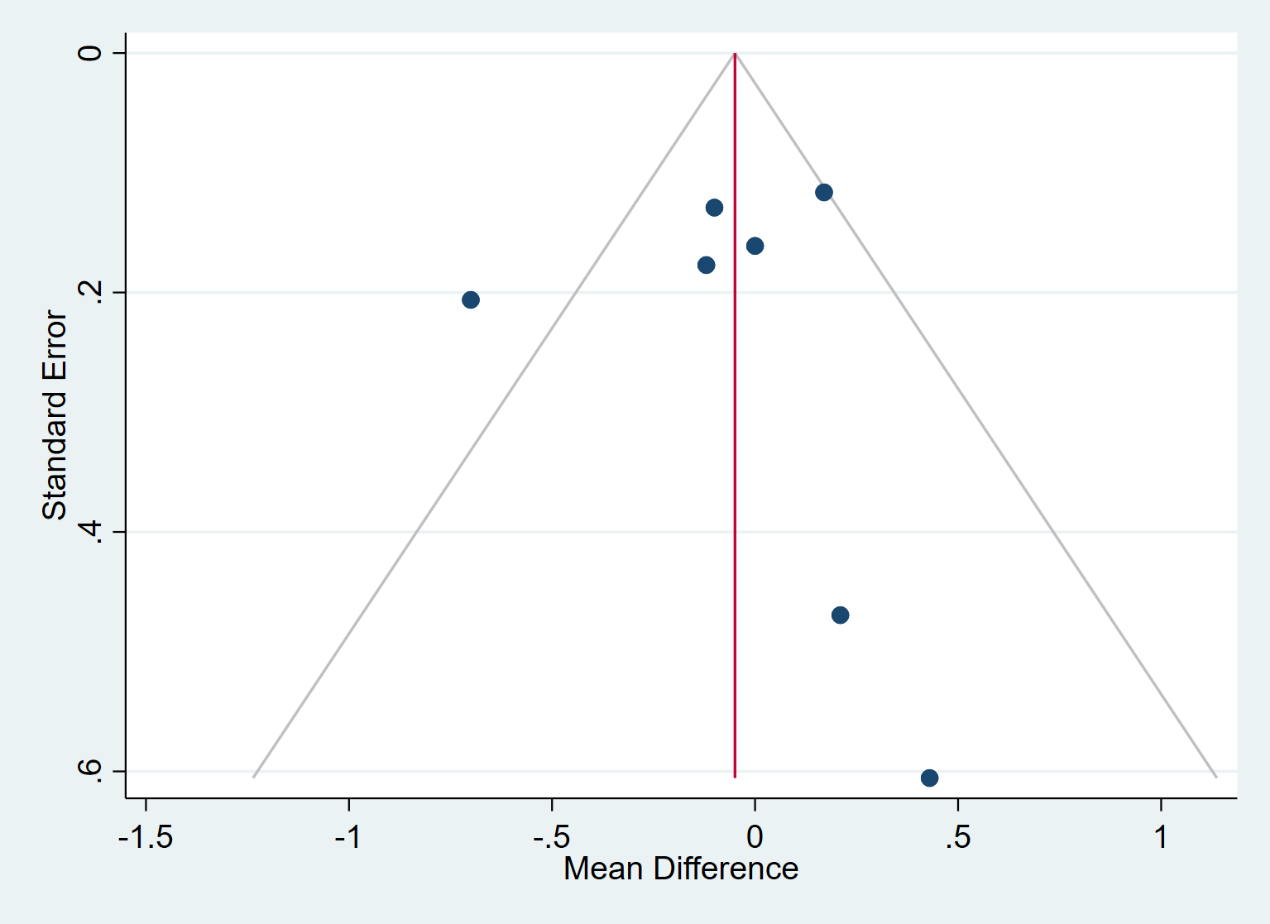


Insulin sensitivity LV-HIIT vs. MICT


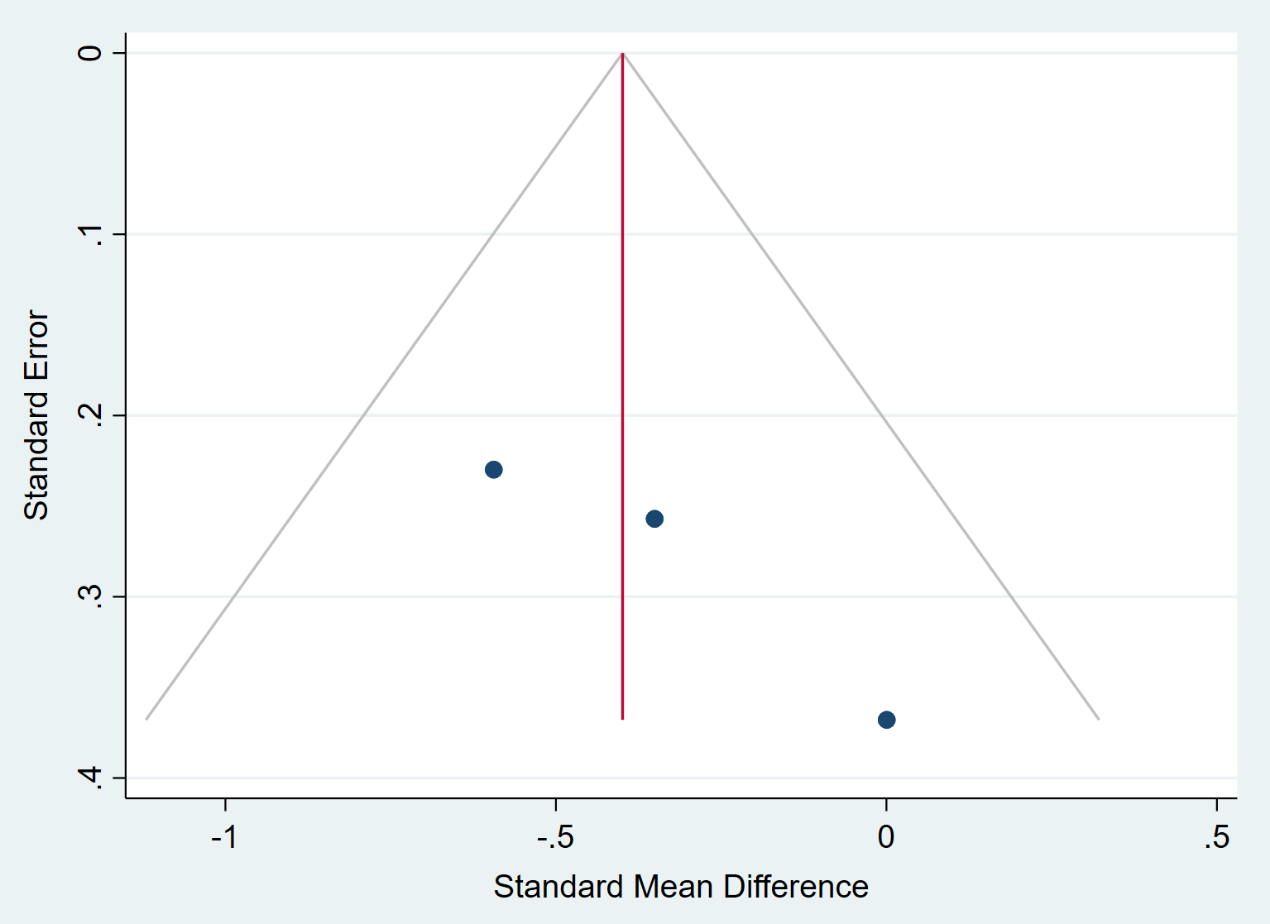


**Supplementary Figure S3:** **Dose-response effects of LV-HIIT on FPG, FPI, HbA1c and HOMA-IR:** results of meta-regression analysis for variables related to an exercise protocol. The effects are presented as mean difference. The circle sizes are proportional to the effect size in each study. A negative value indicates a larger improvement as a result of low-volume high-intensity interval training compared with no exercise. The dashed line represents the 95% CI of the regression line.


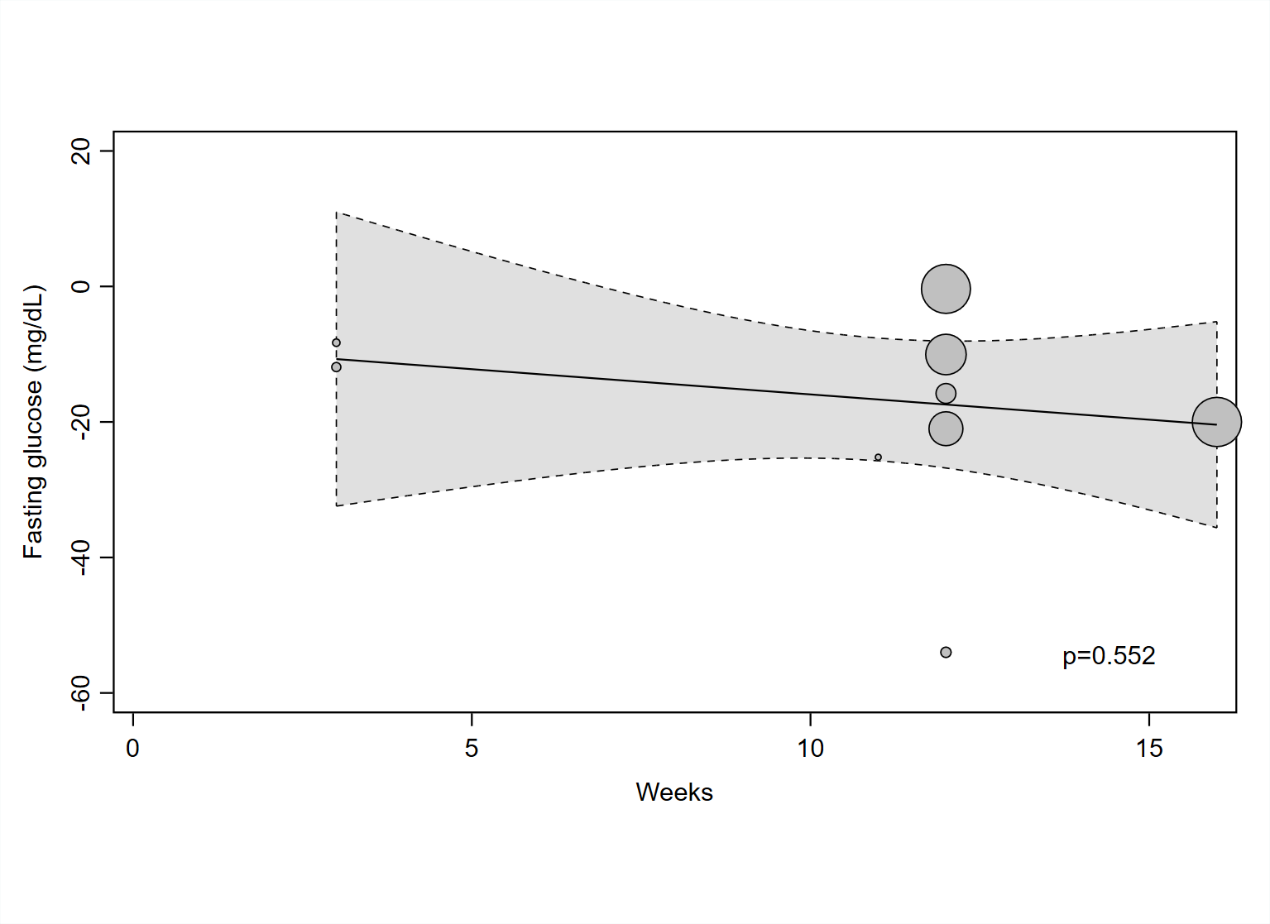

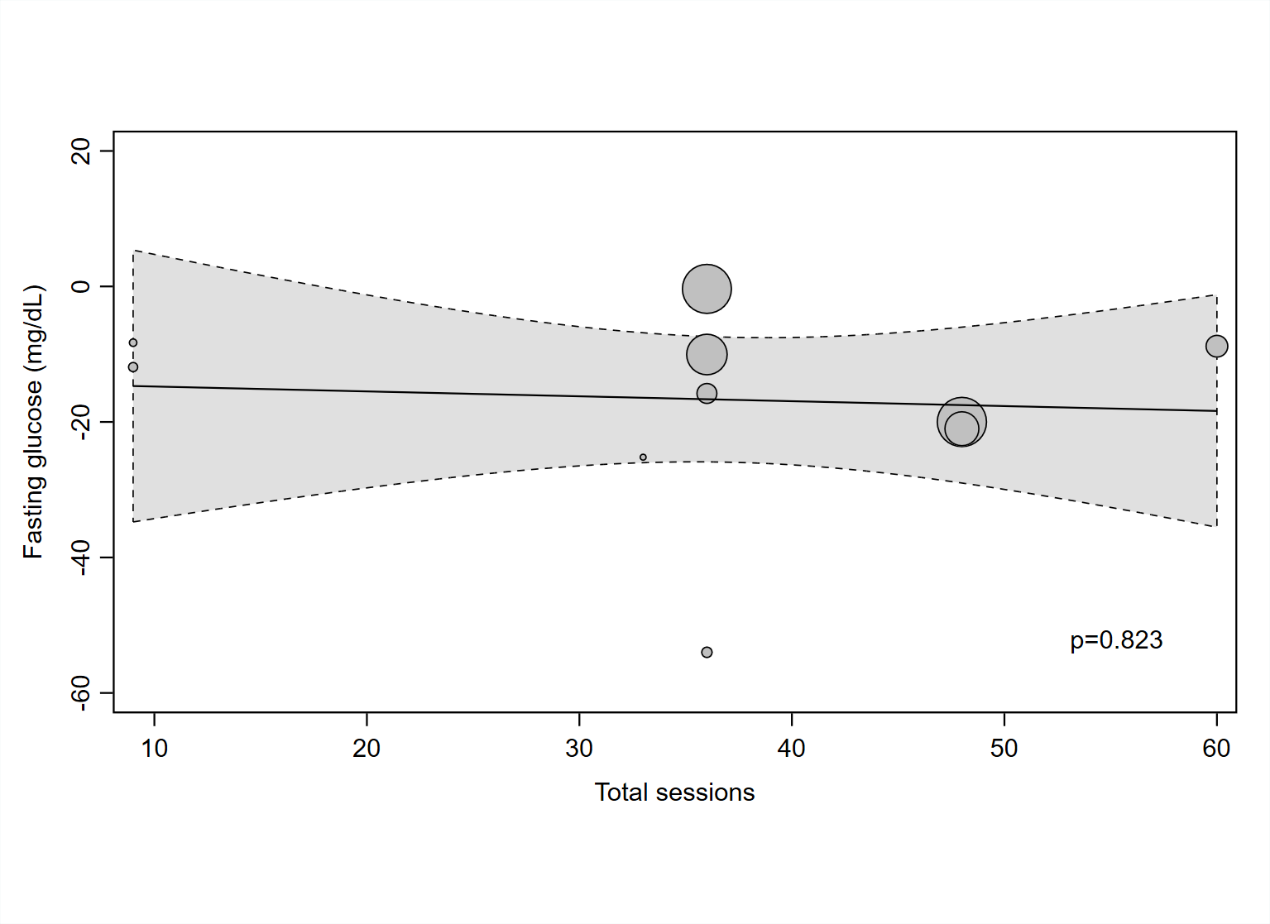


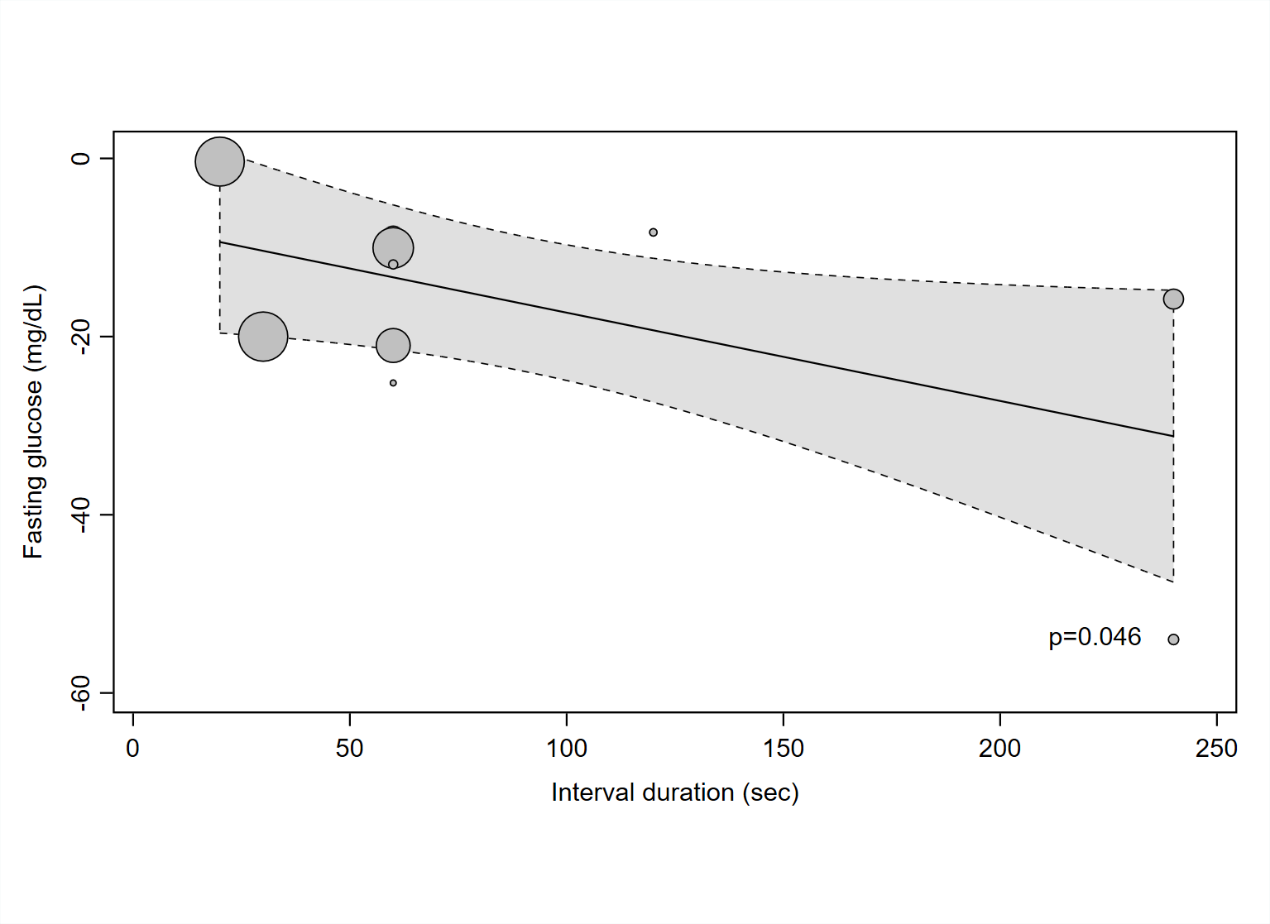


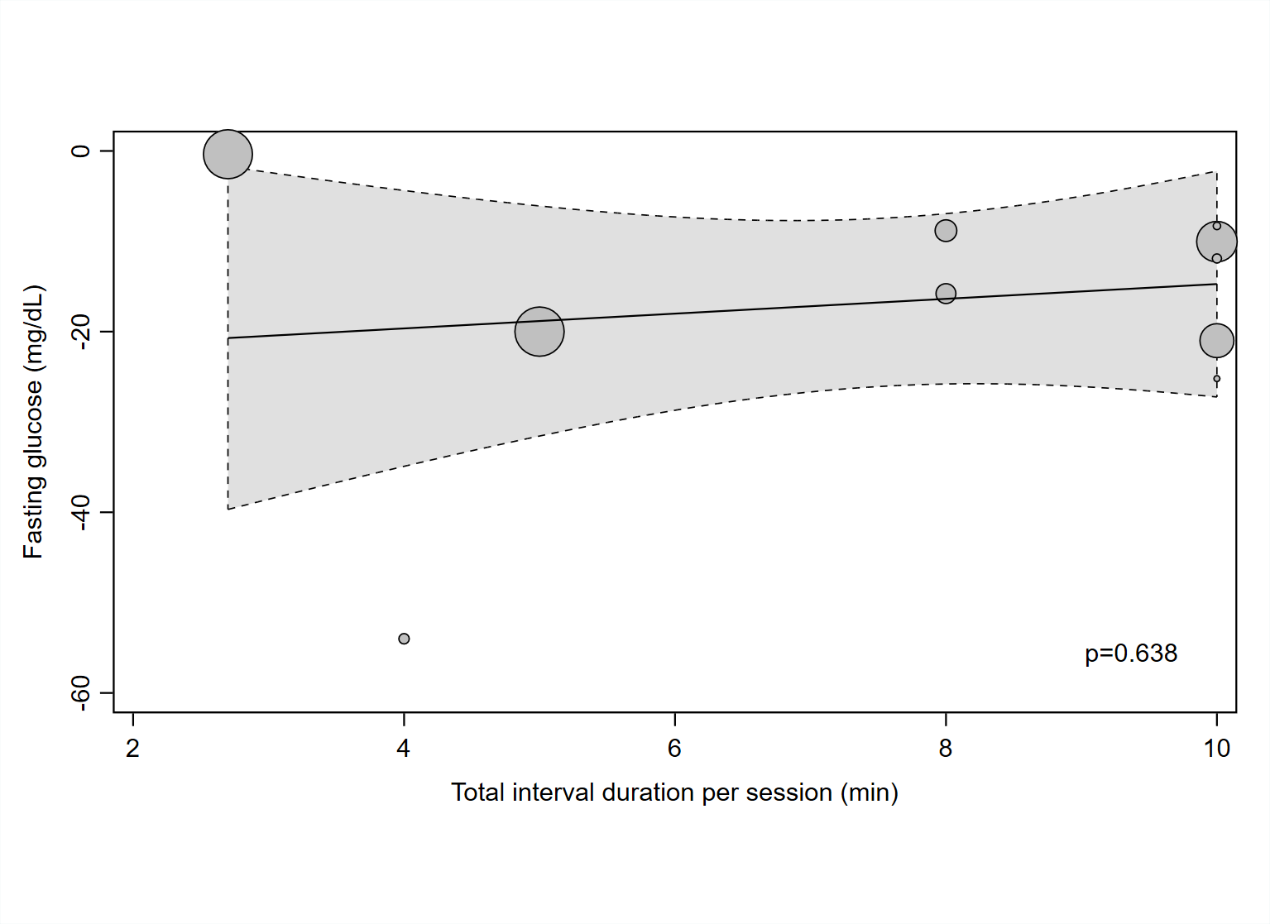

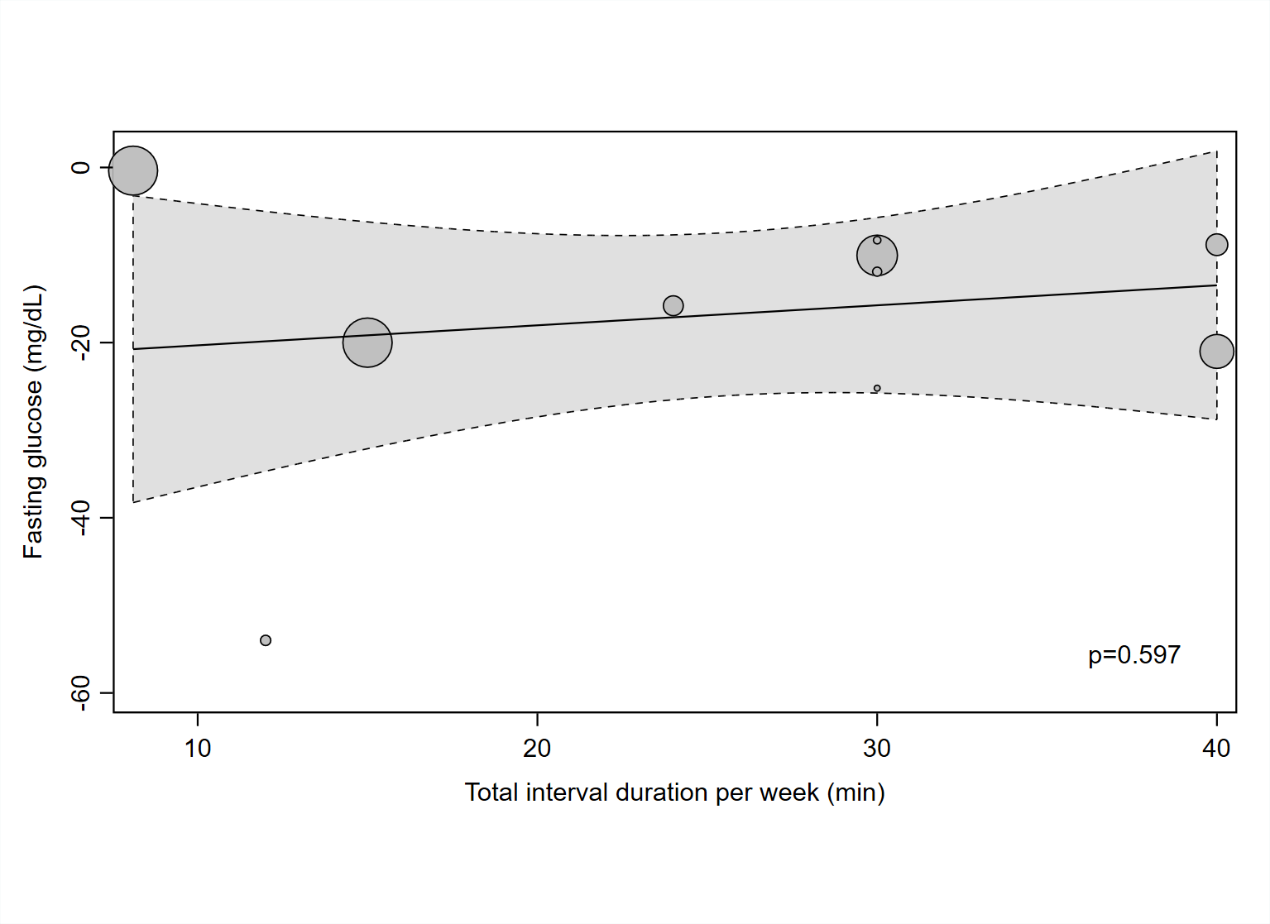

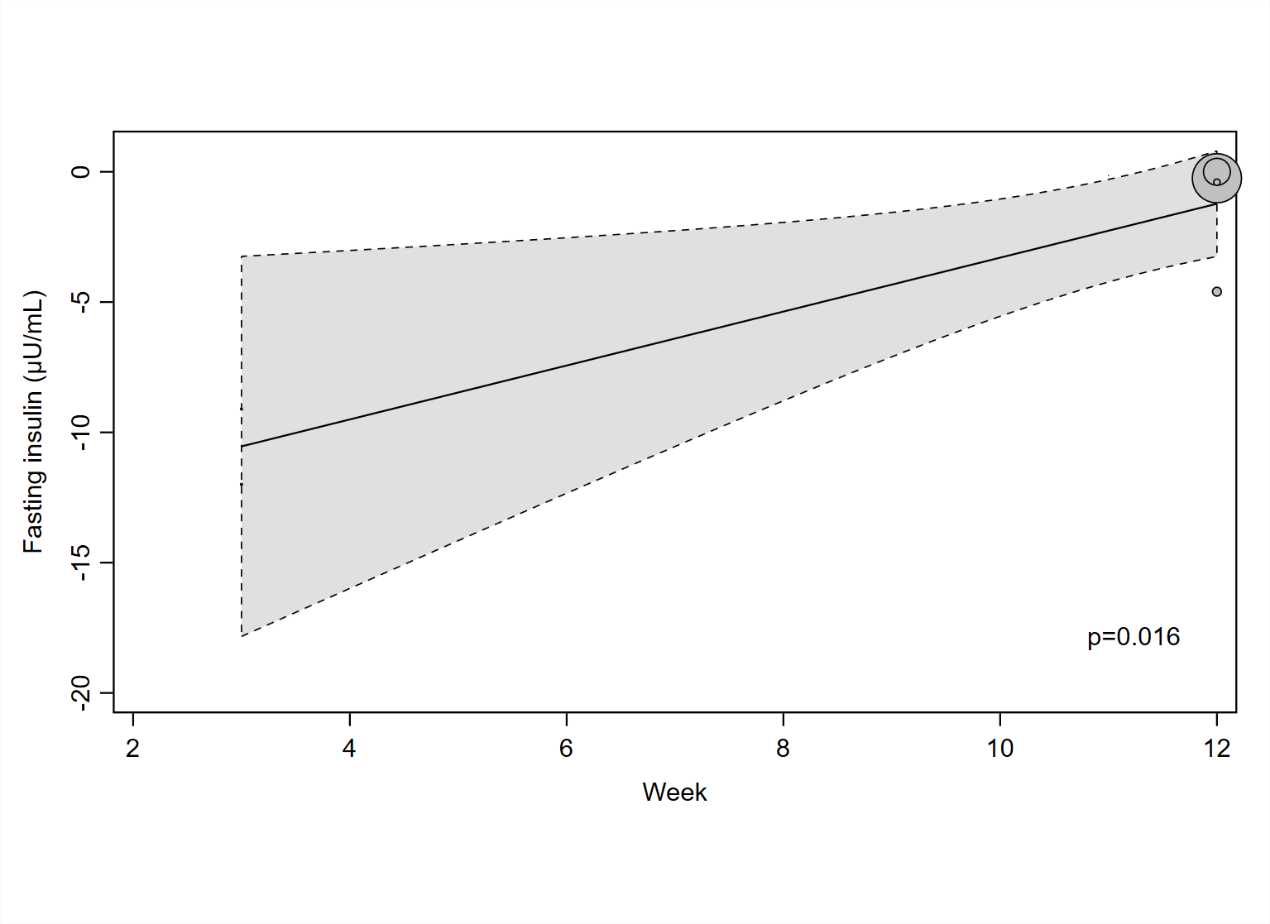

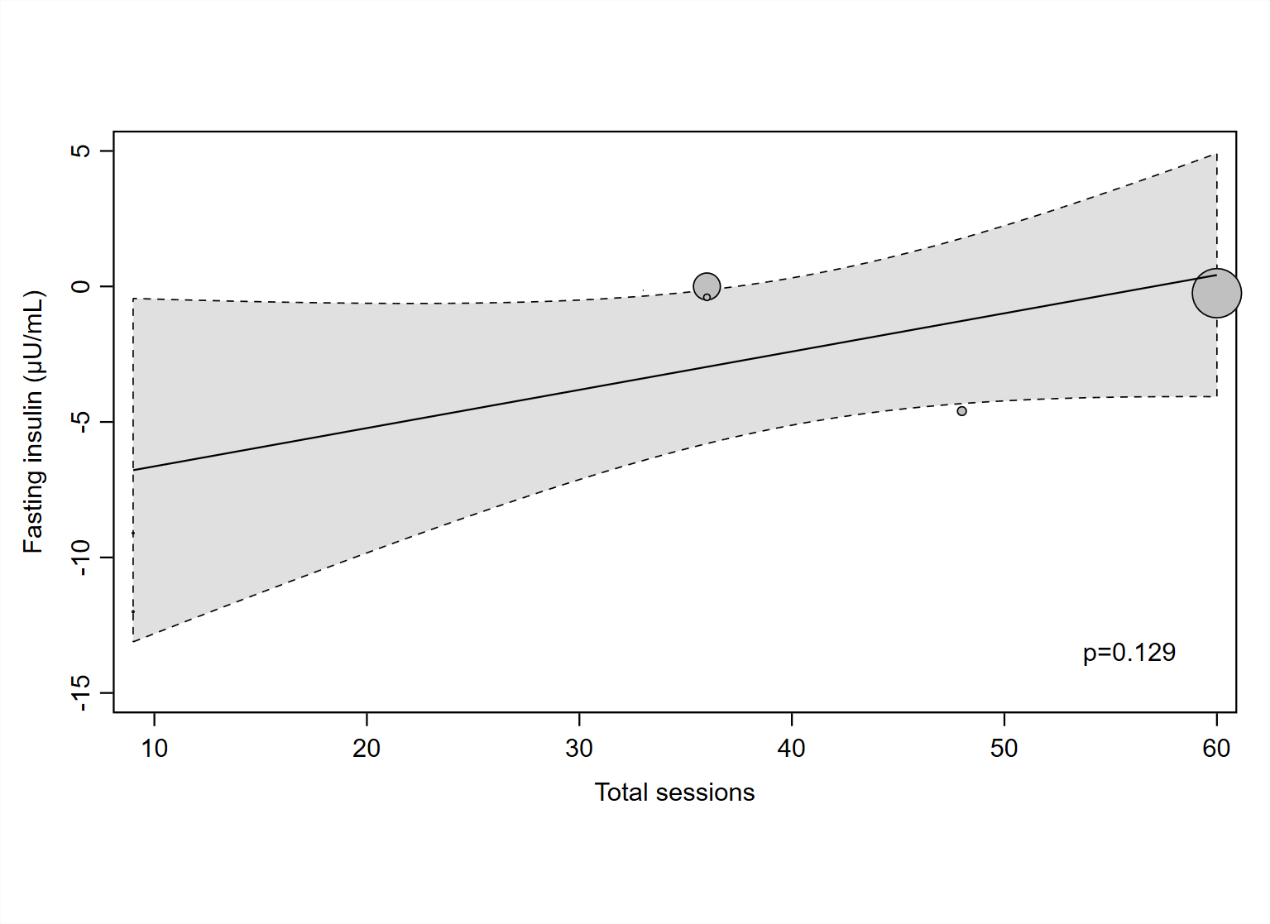

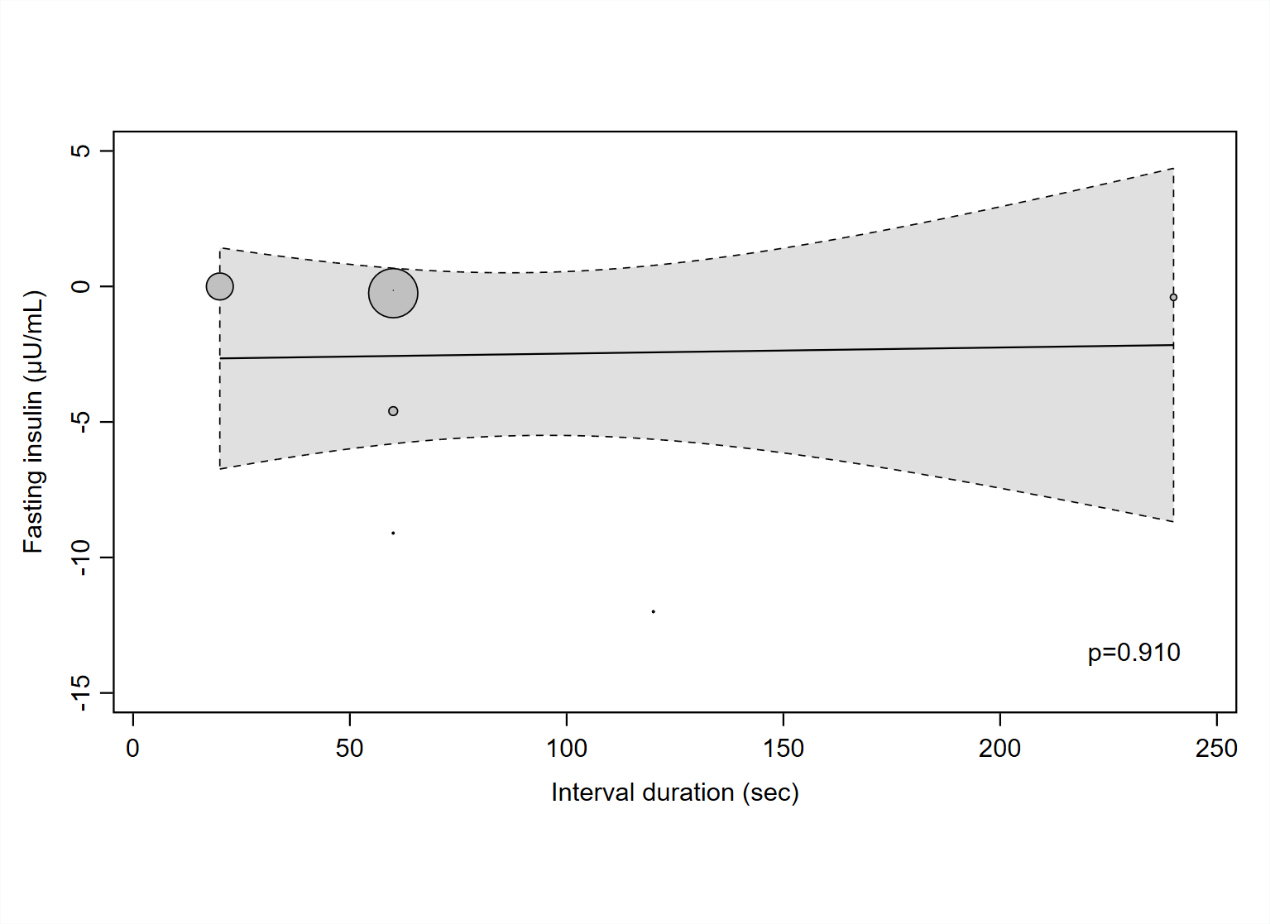

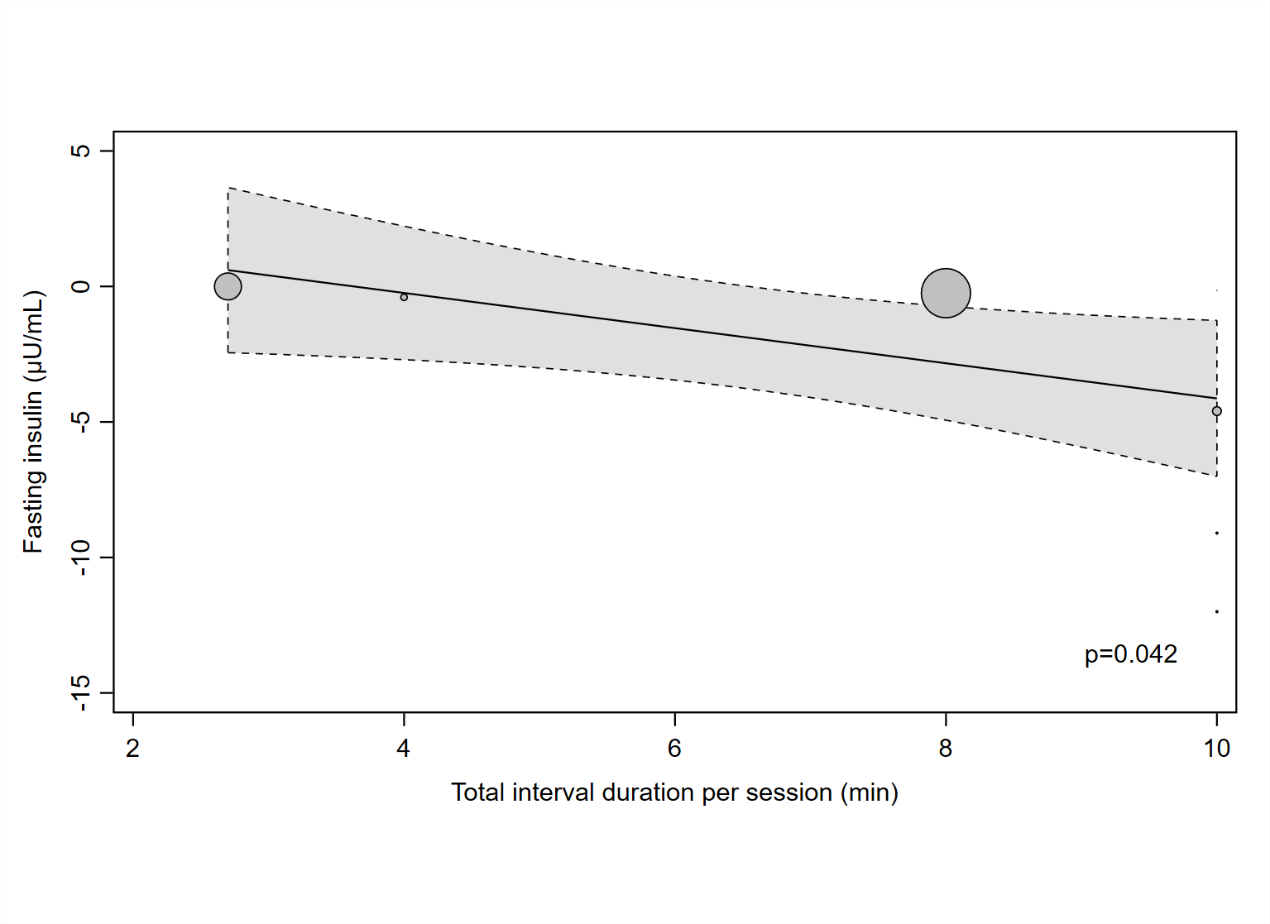

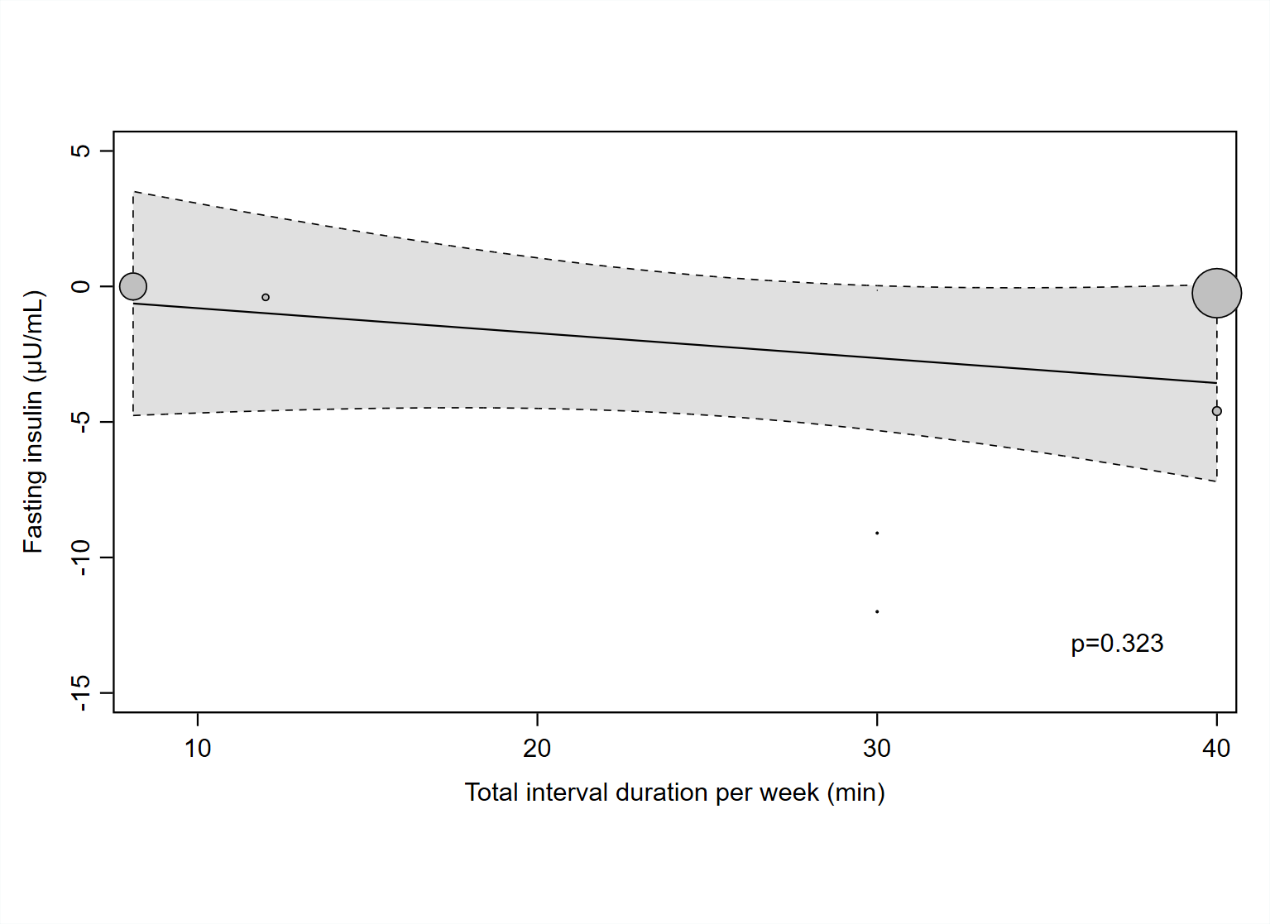

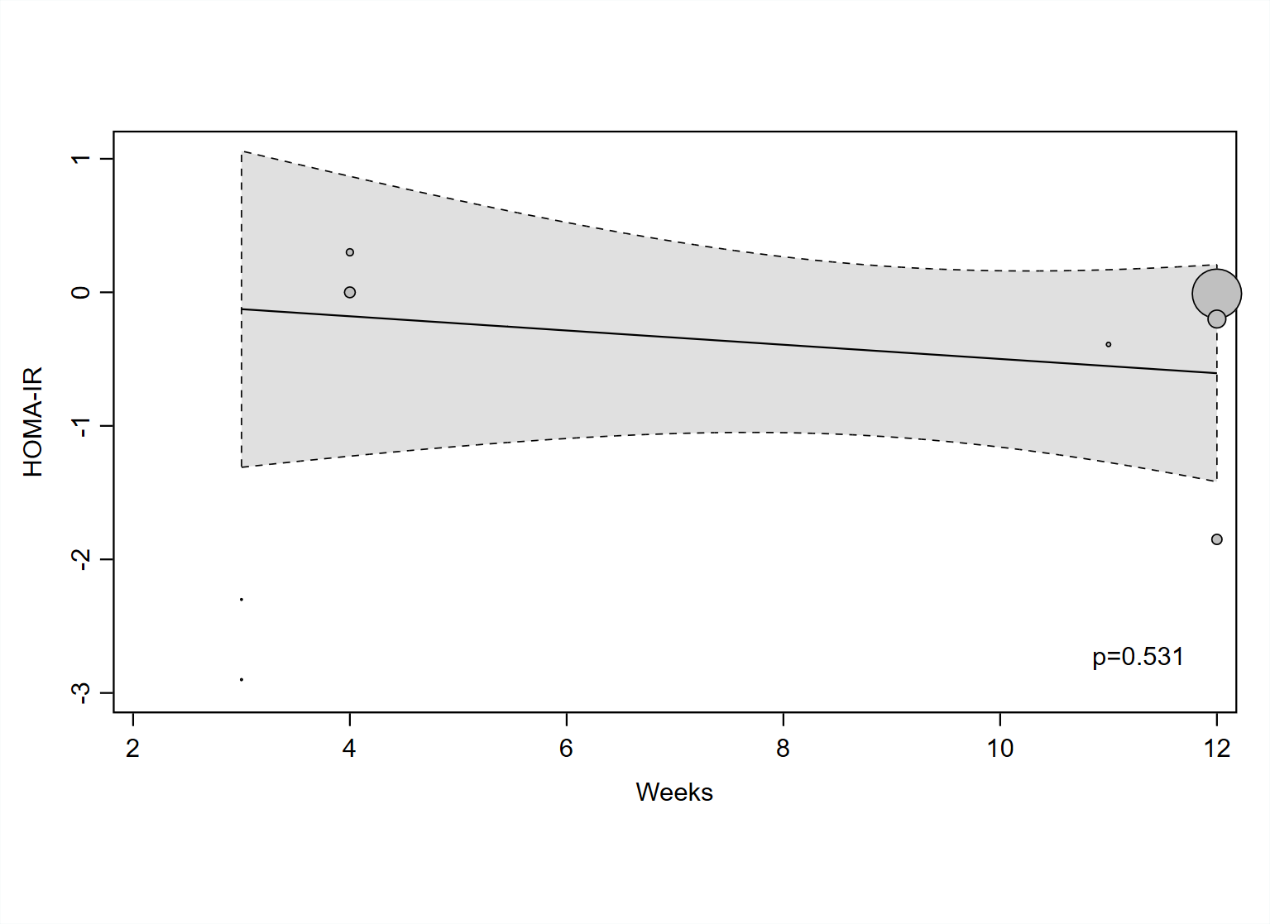

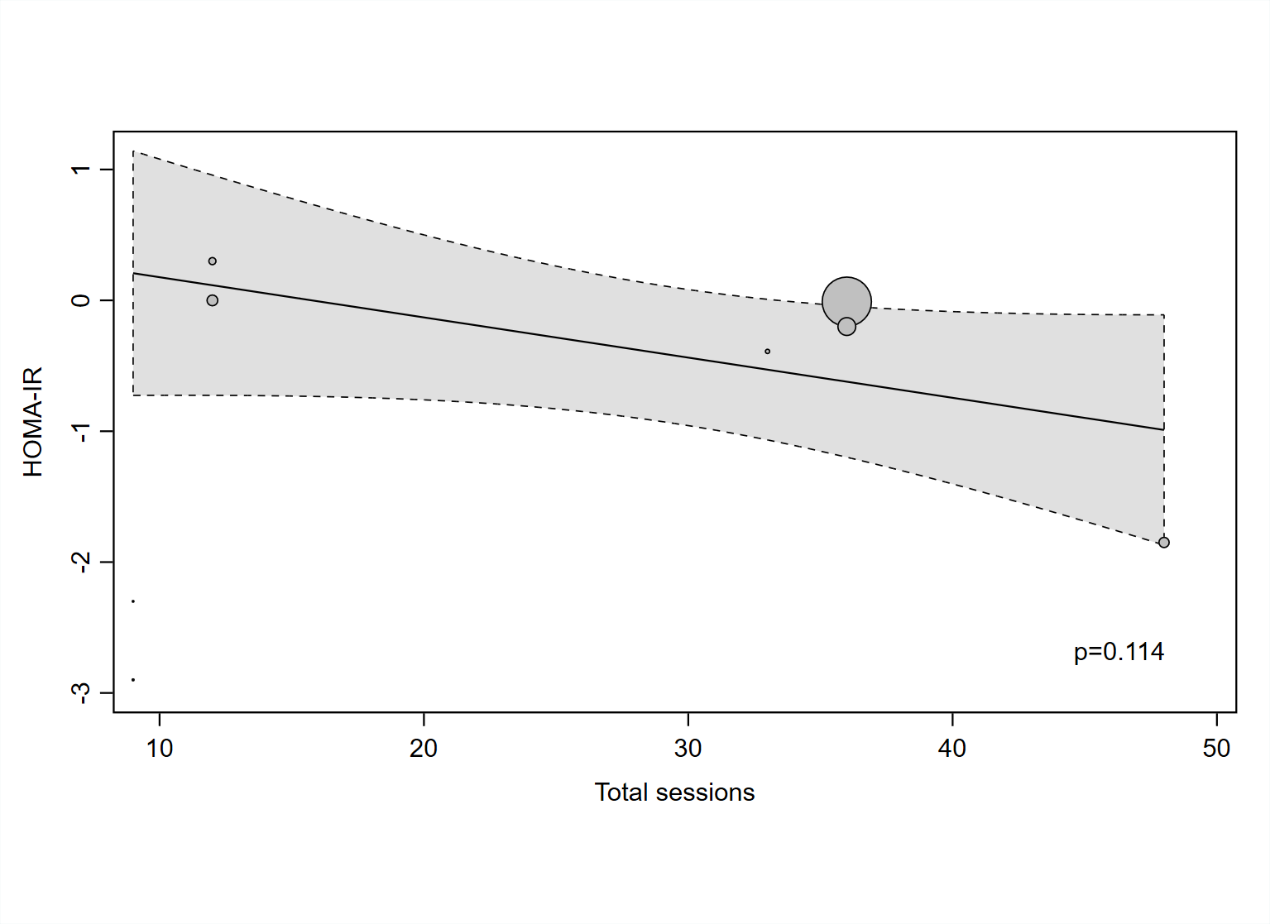

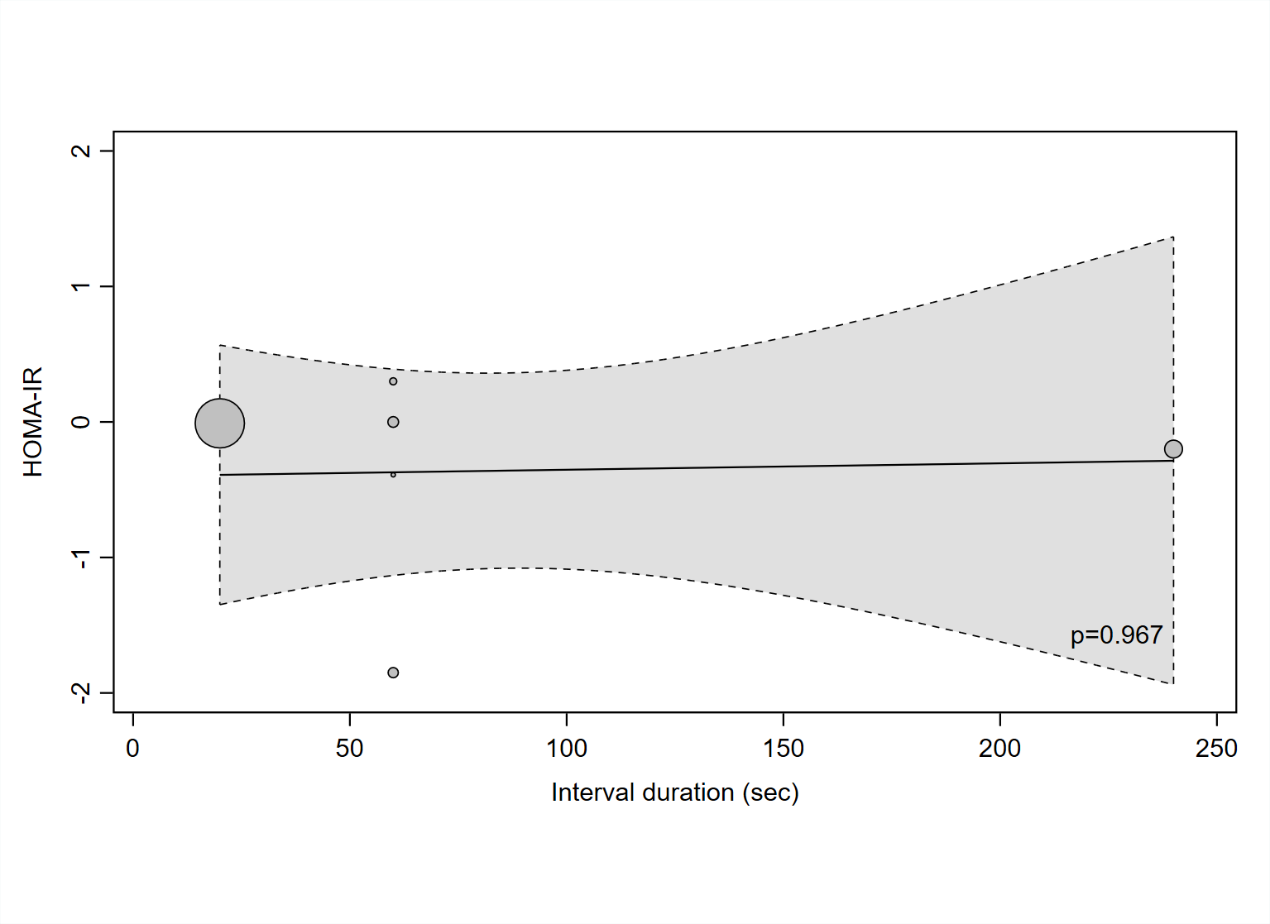

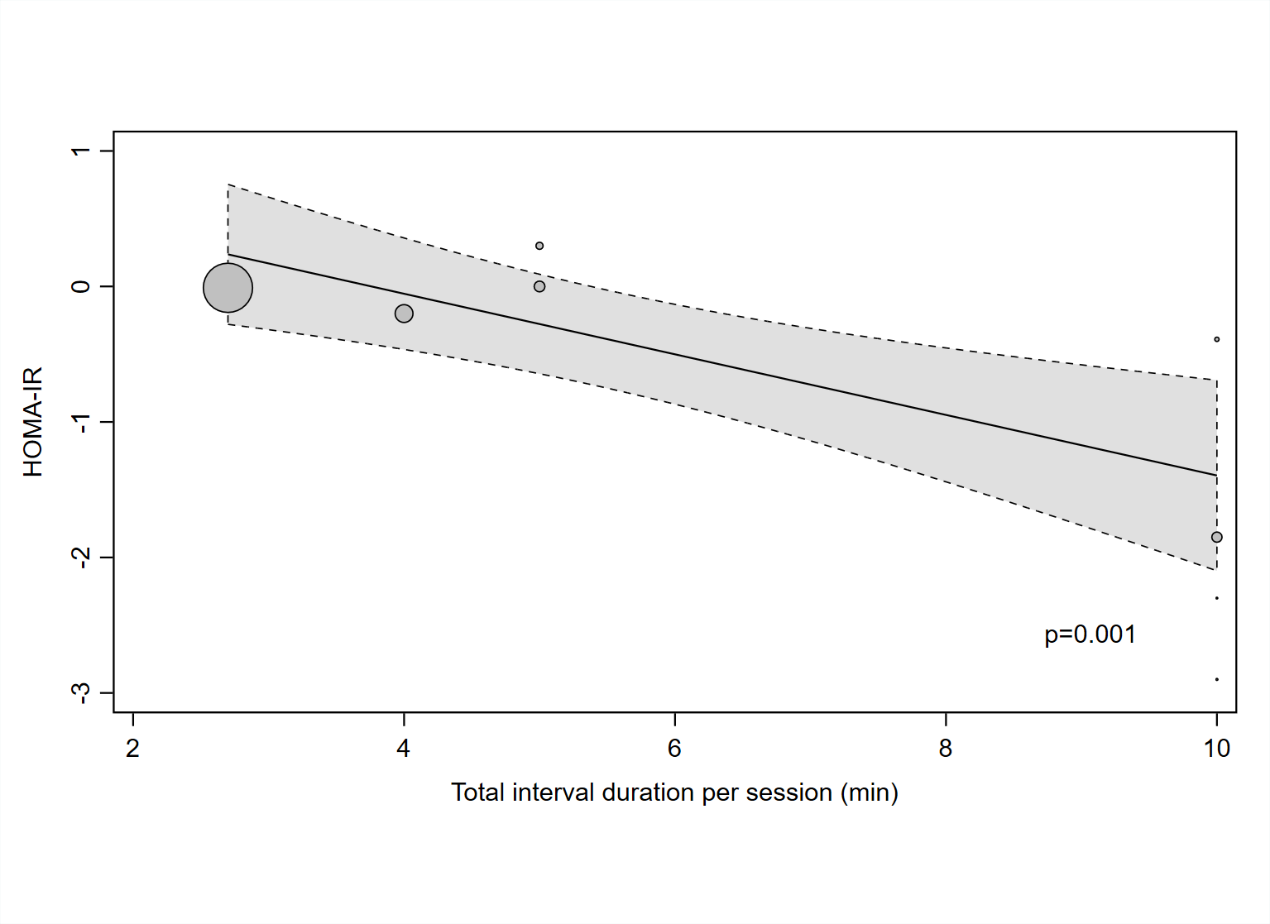

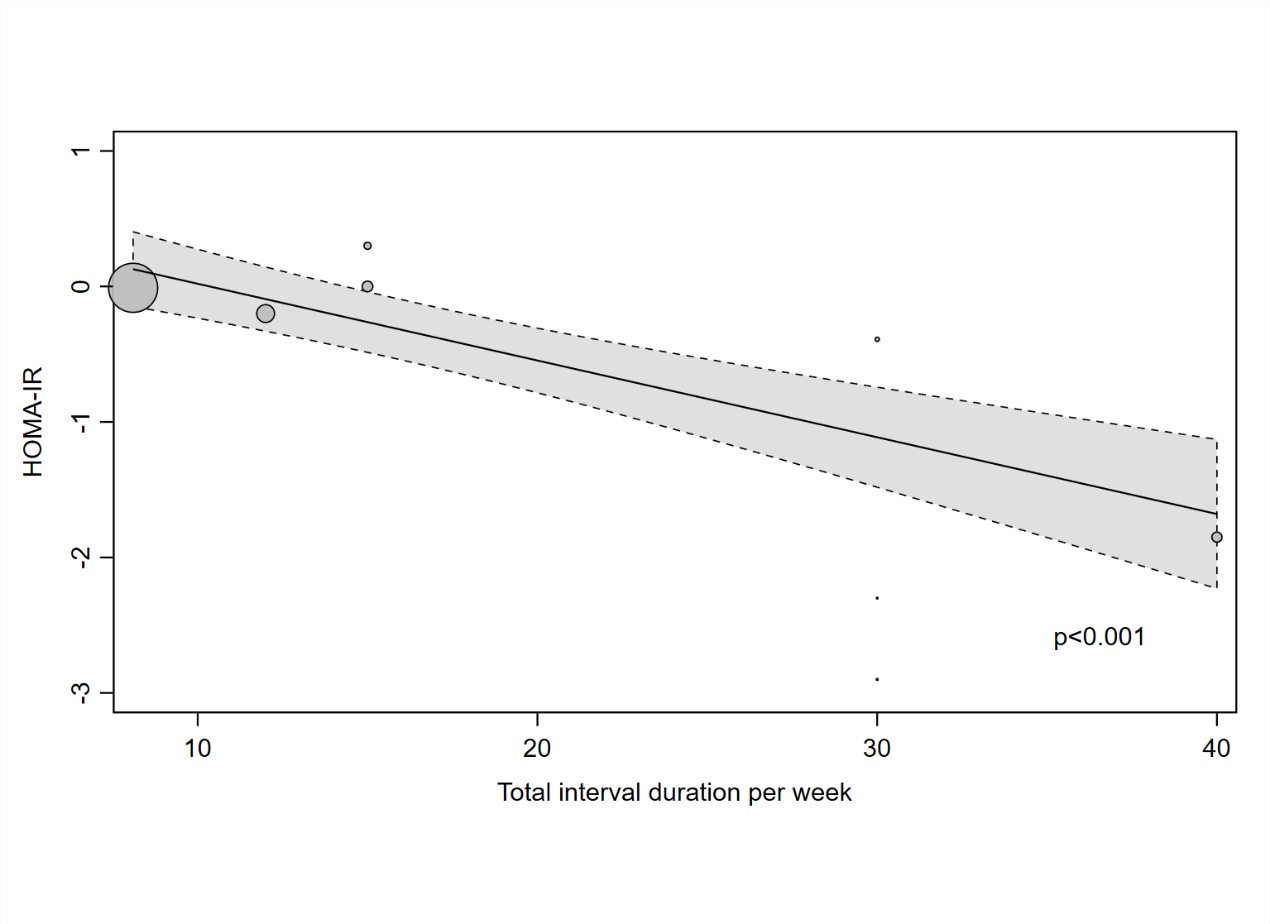

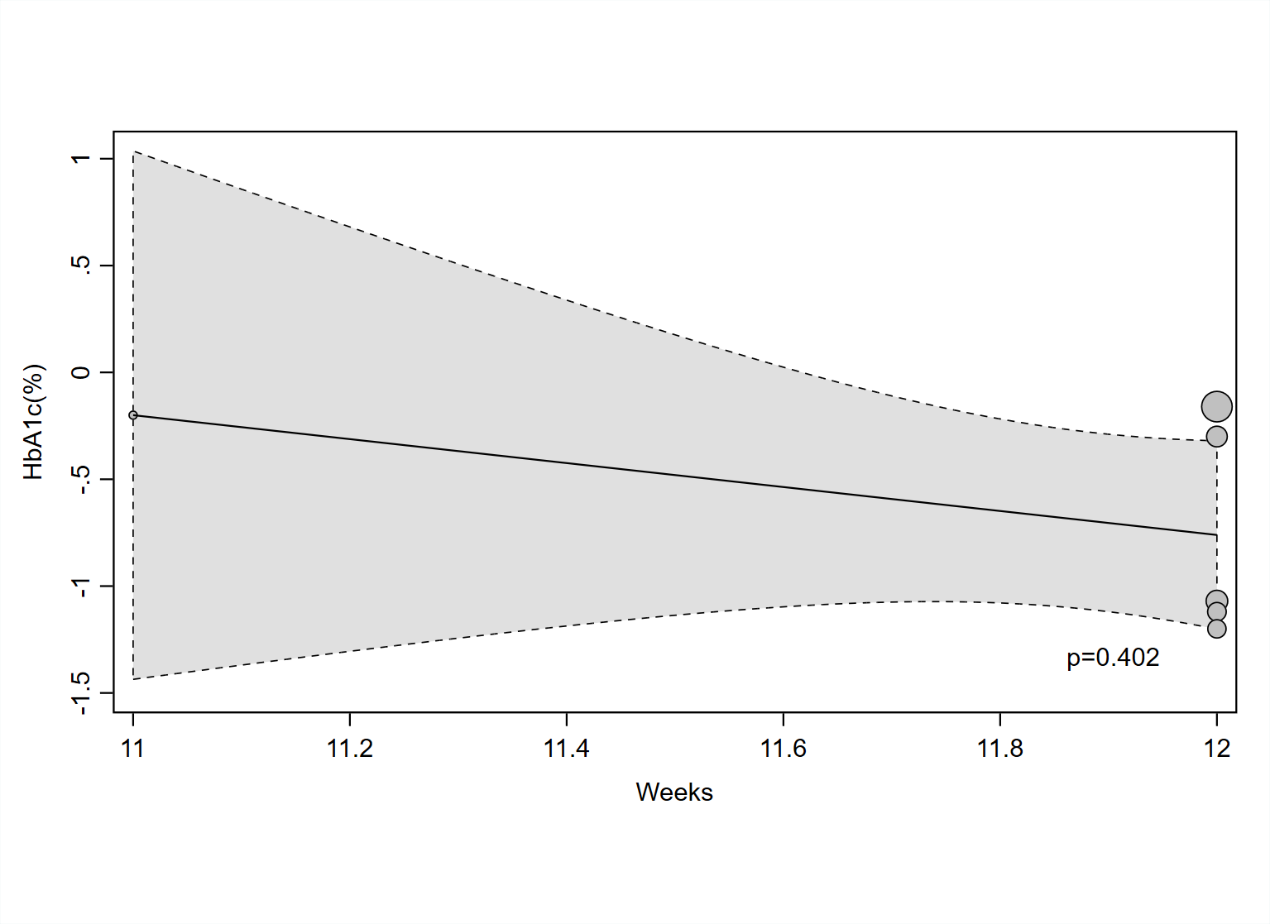

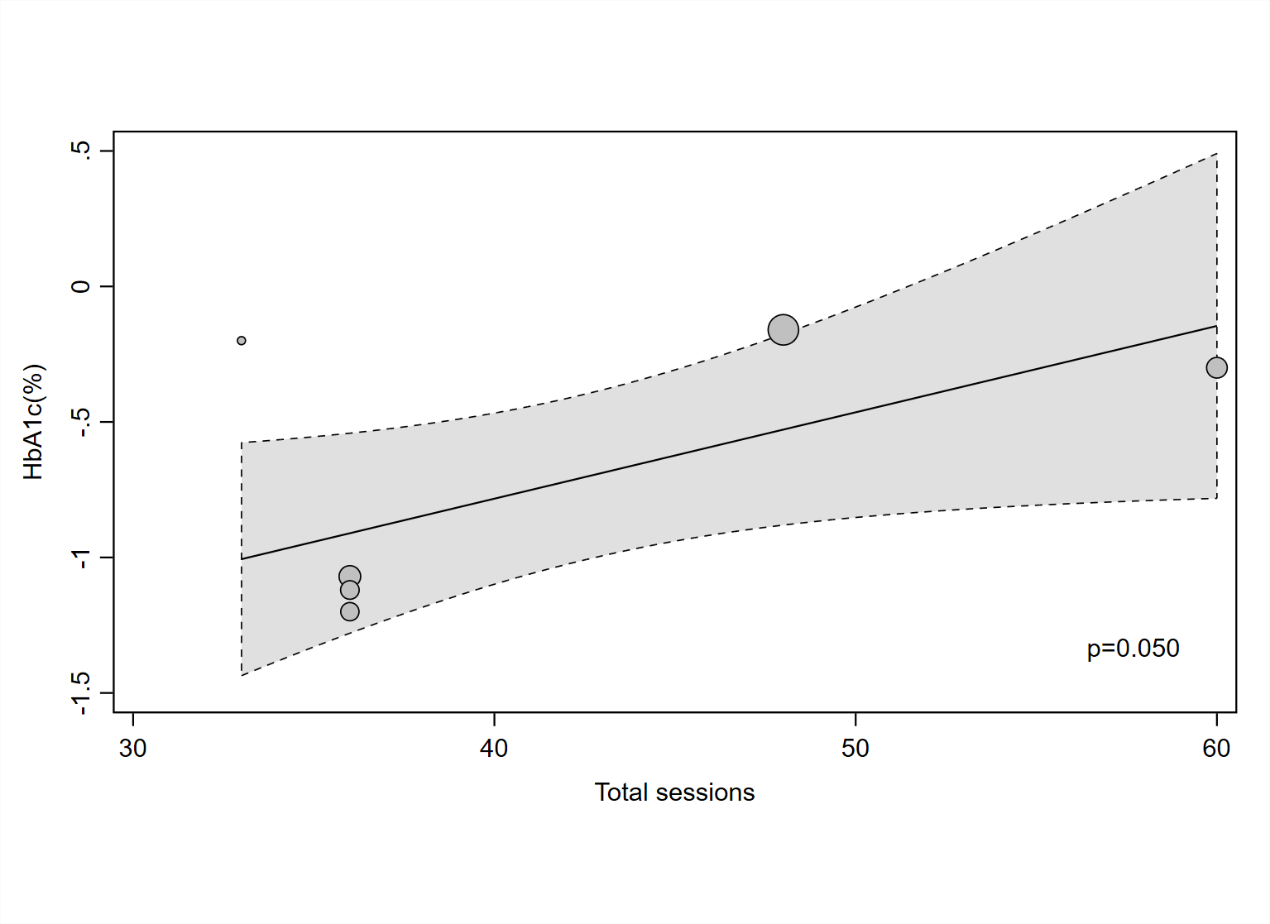

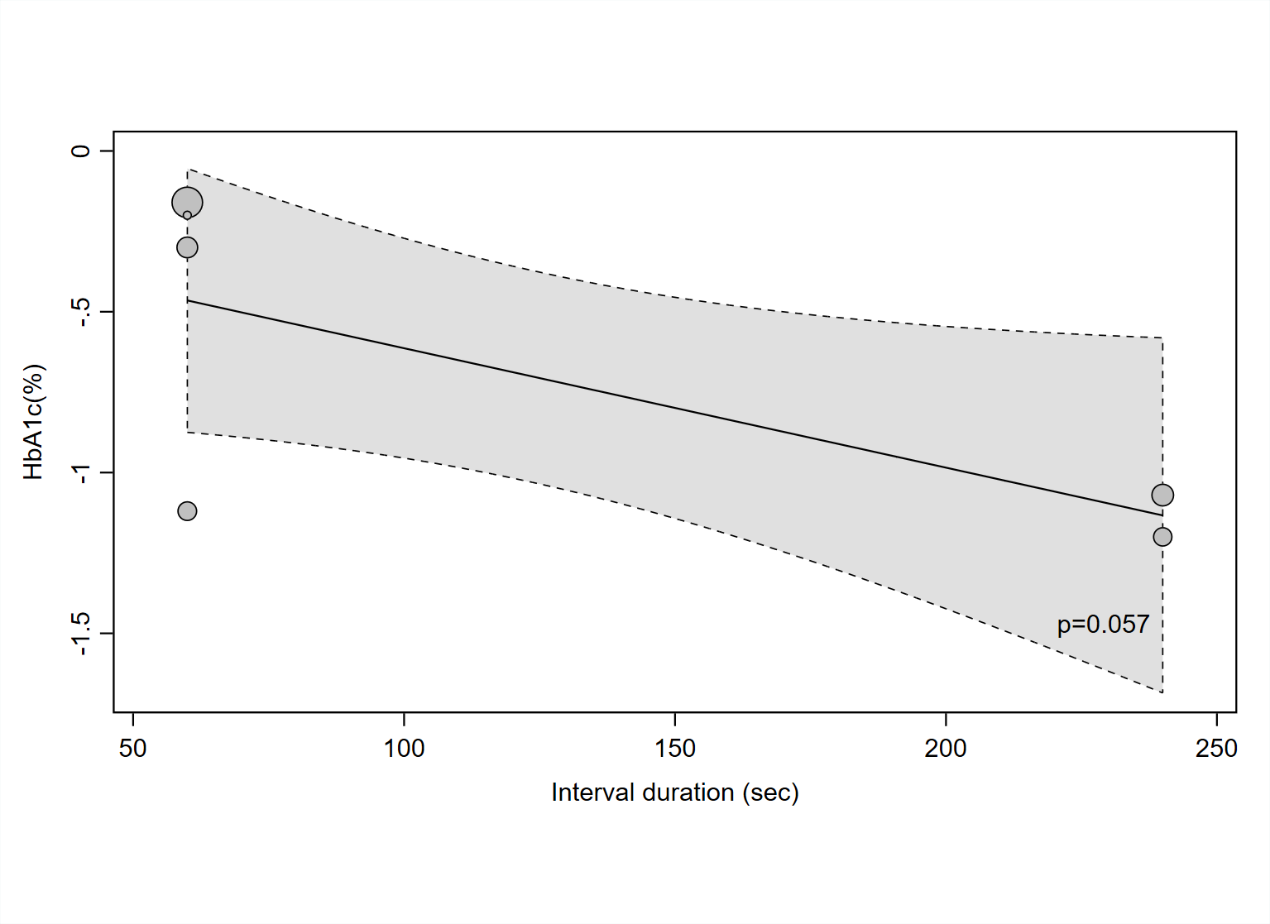

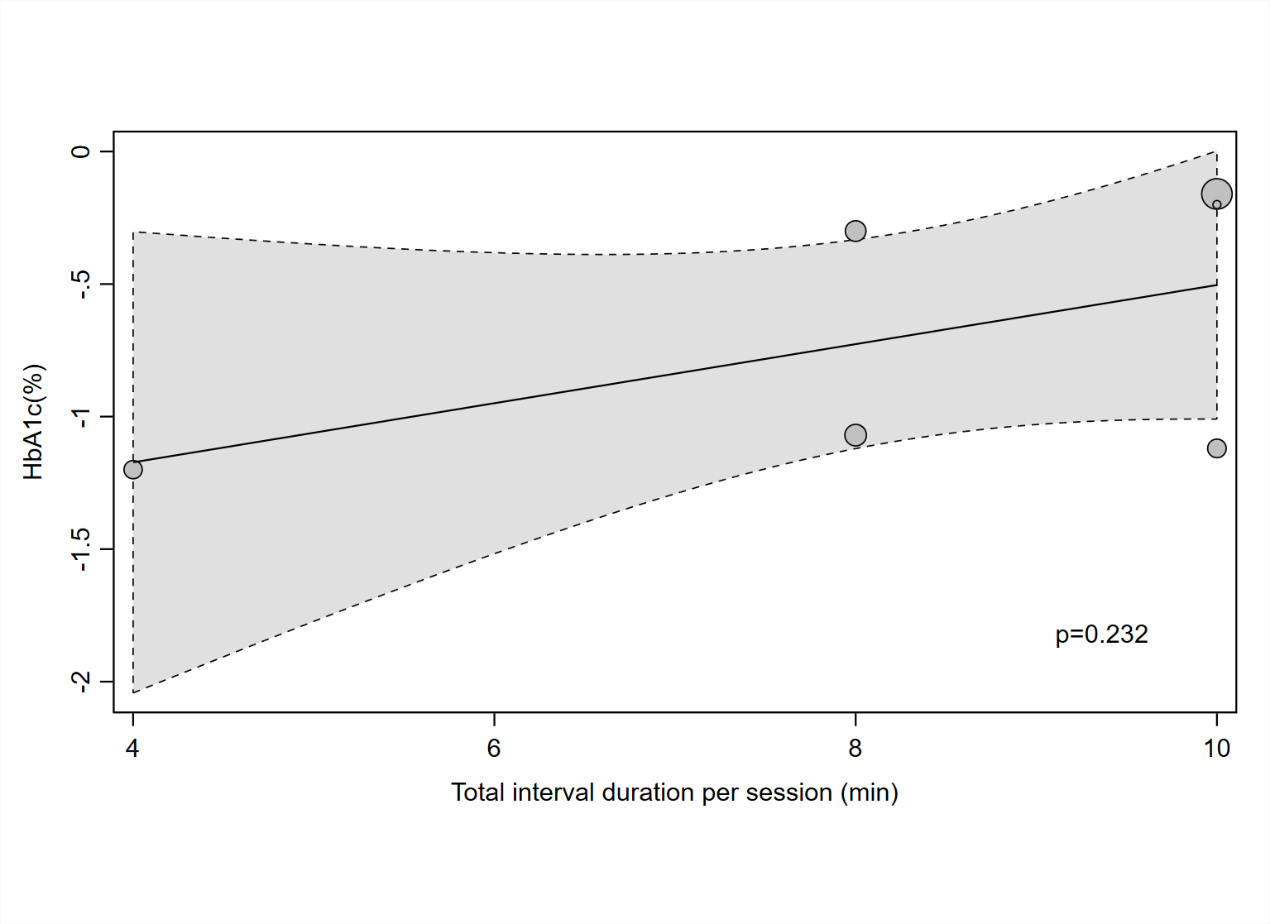

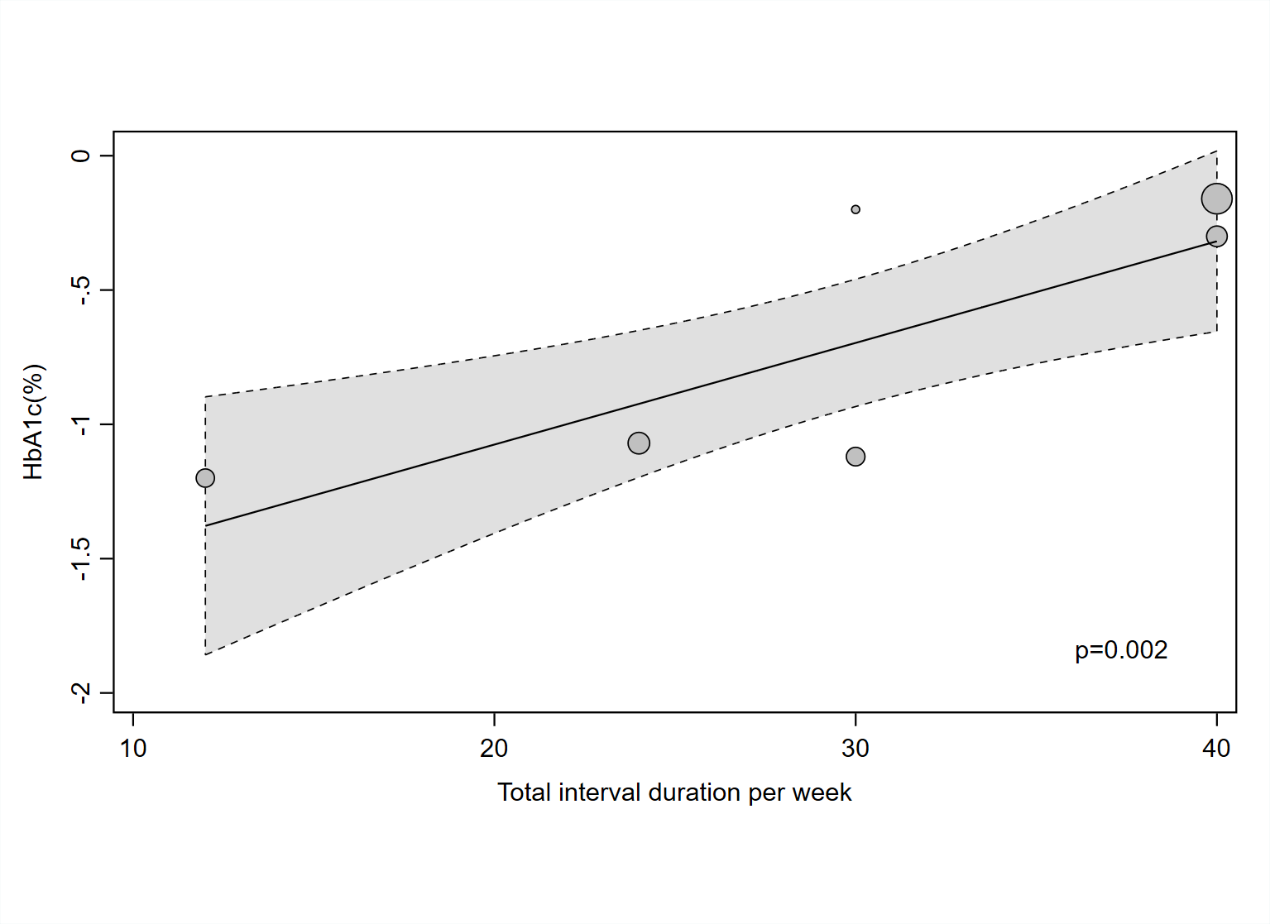


**Supplementary Figure S4: Dose-response effects of low-volume high-intensity interval training on fasting glucose, fasting insulin, HbA1c and HOMA-IR compared with moderate-intensity continuous training:** results of meta-regression analysis for variables related to an exercise protocol. The effects are presented as mean difference. The circle sizes are proportional to the effect size in each study. A negative value indicates a larger improvement as a result of low-volume high-intensity interval training compared with moderate-intensity continuous training. The dashed line represents the 95% CI of the regression line.


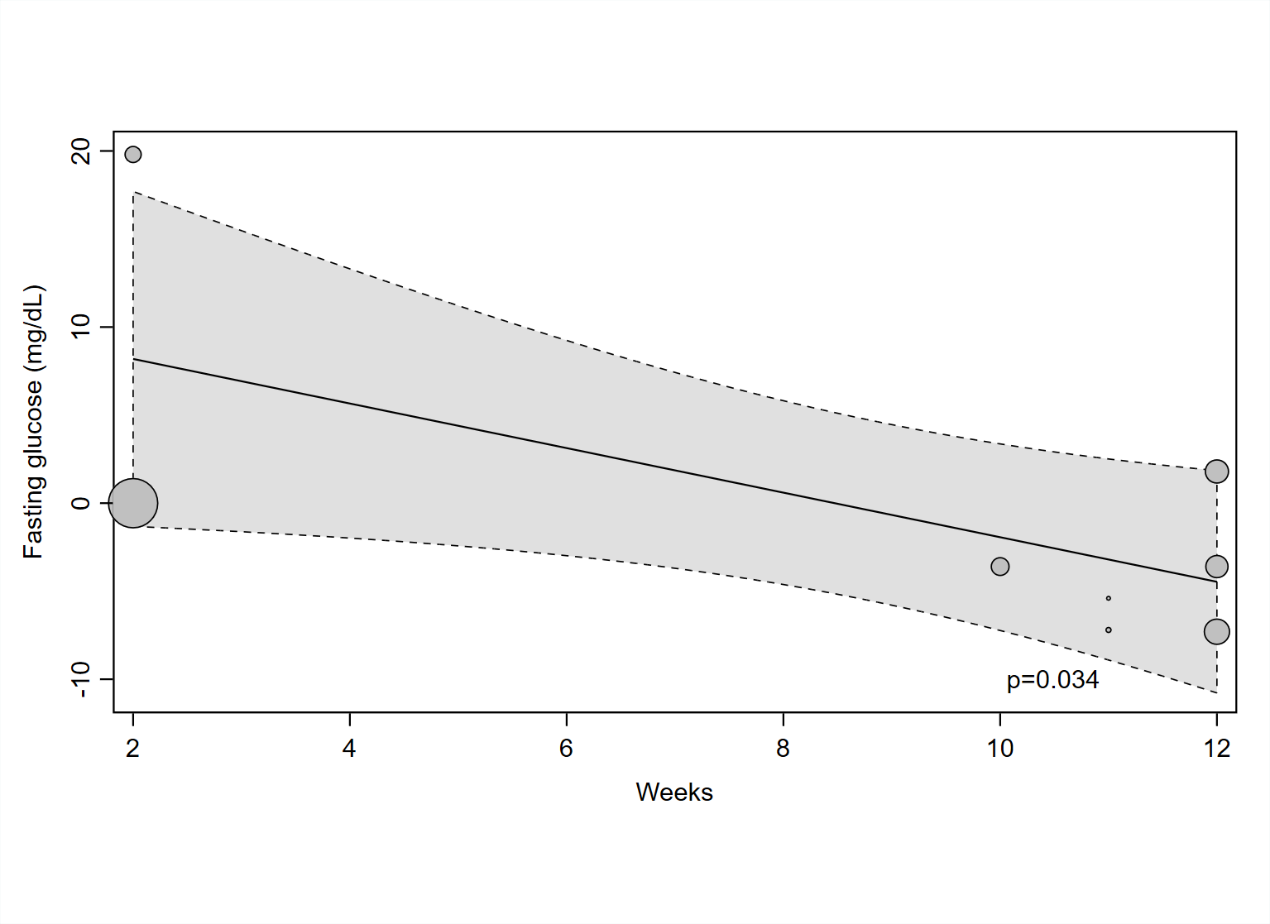

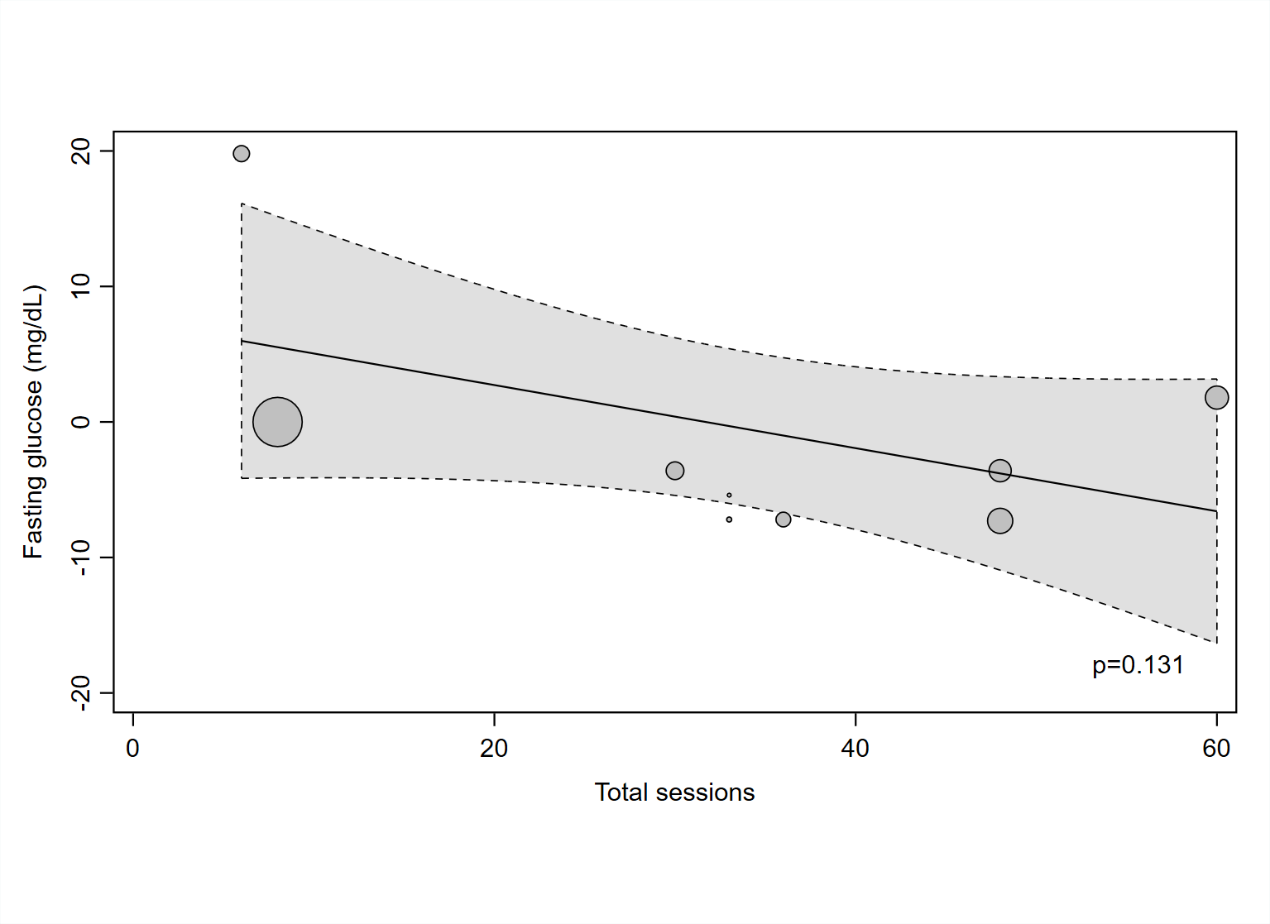

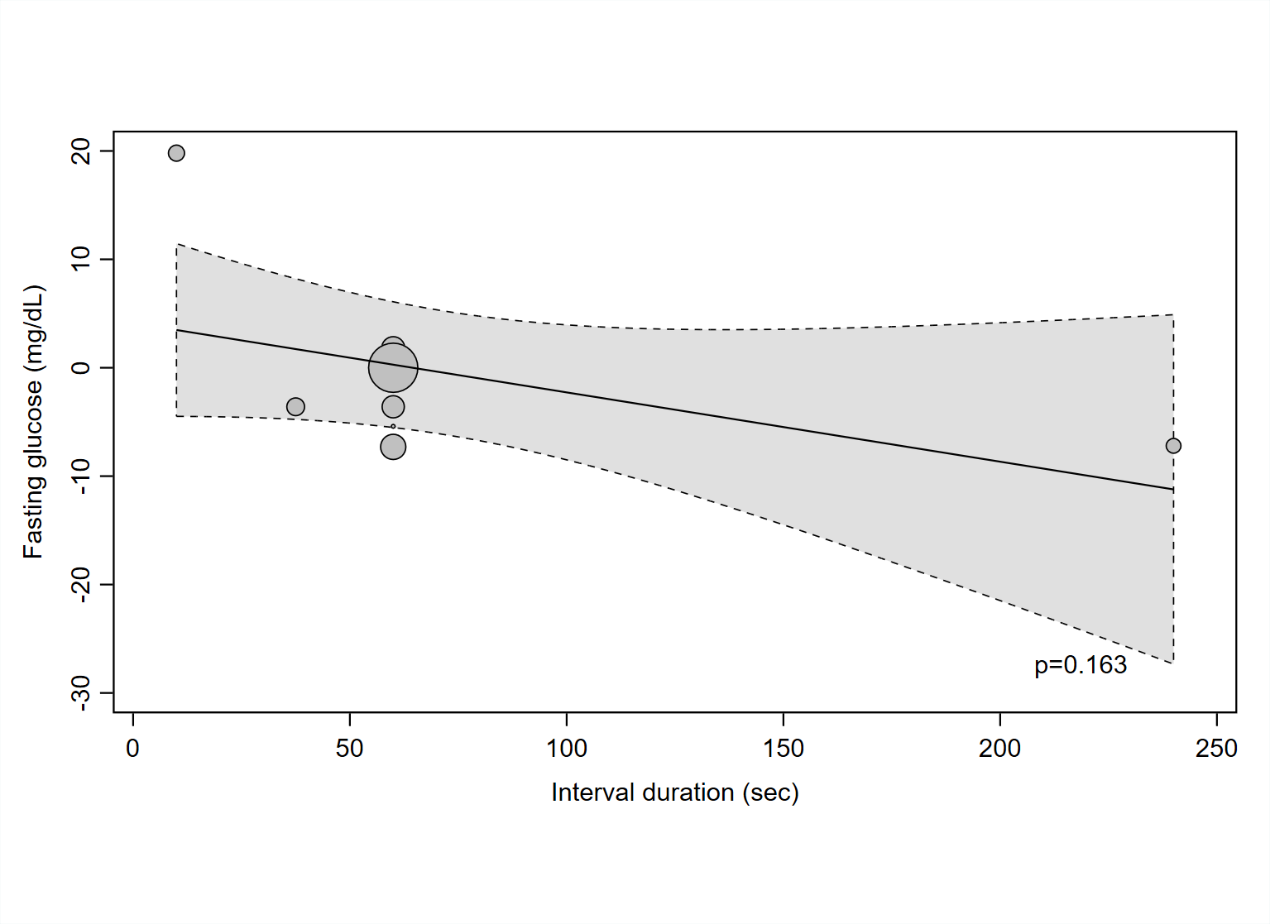

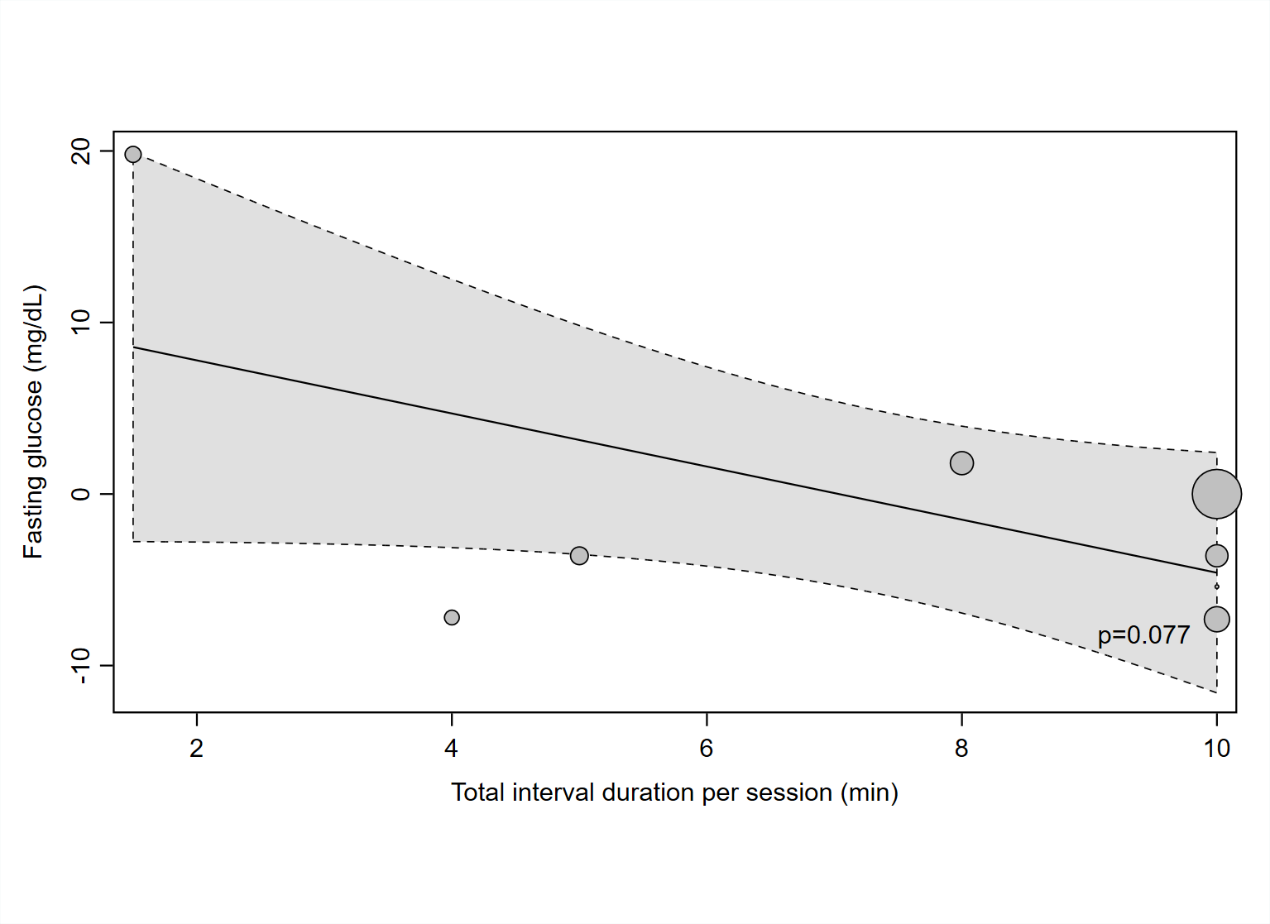

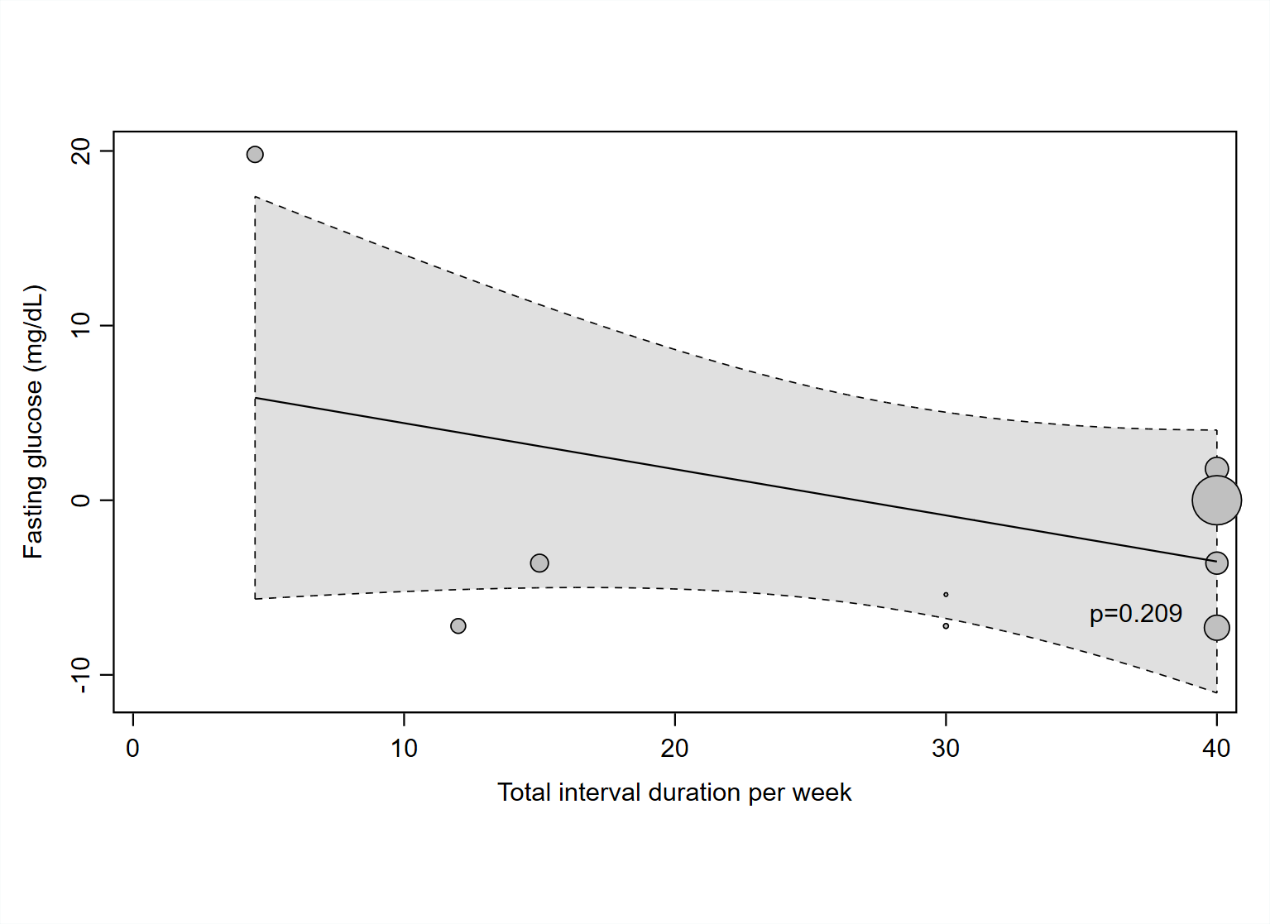

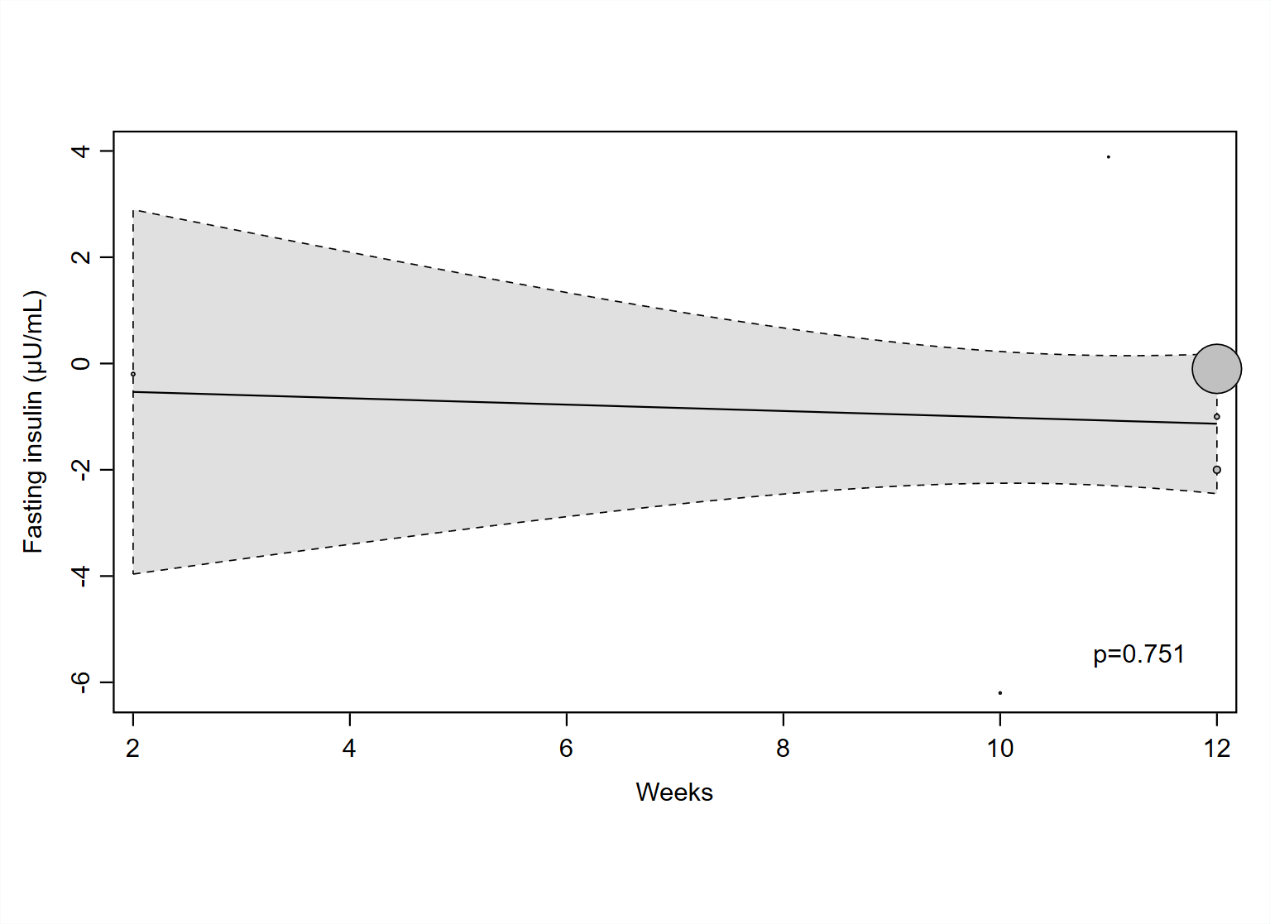

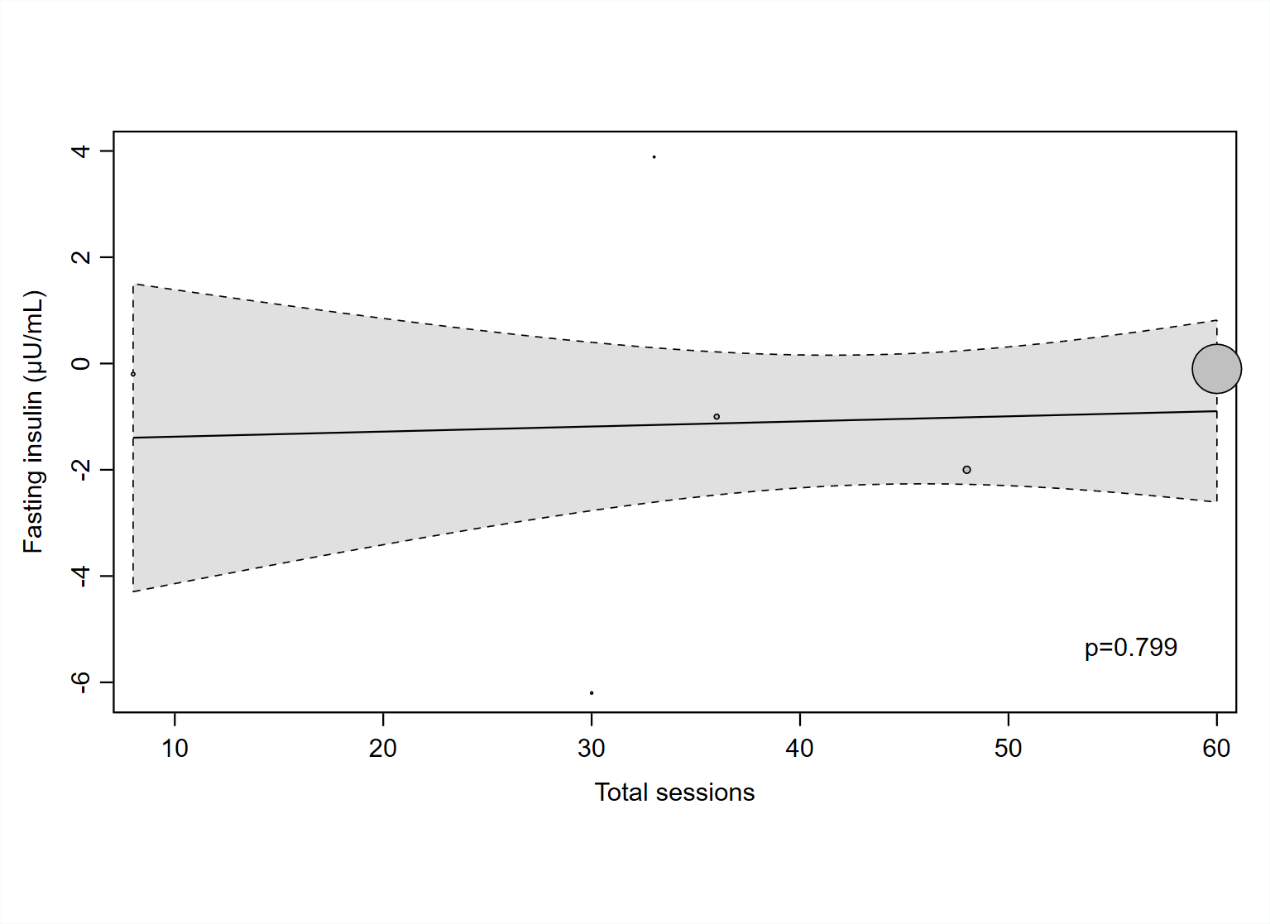

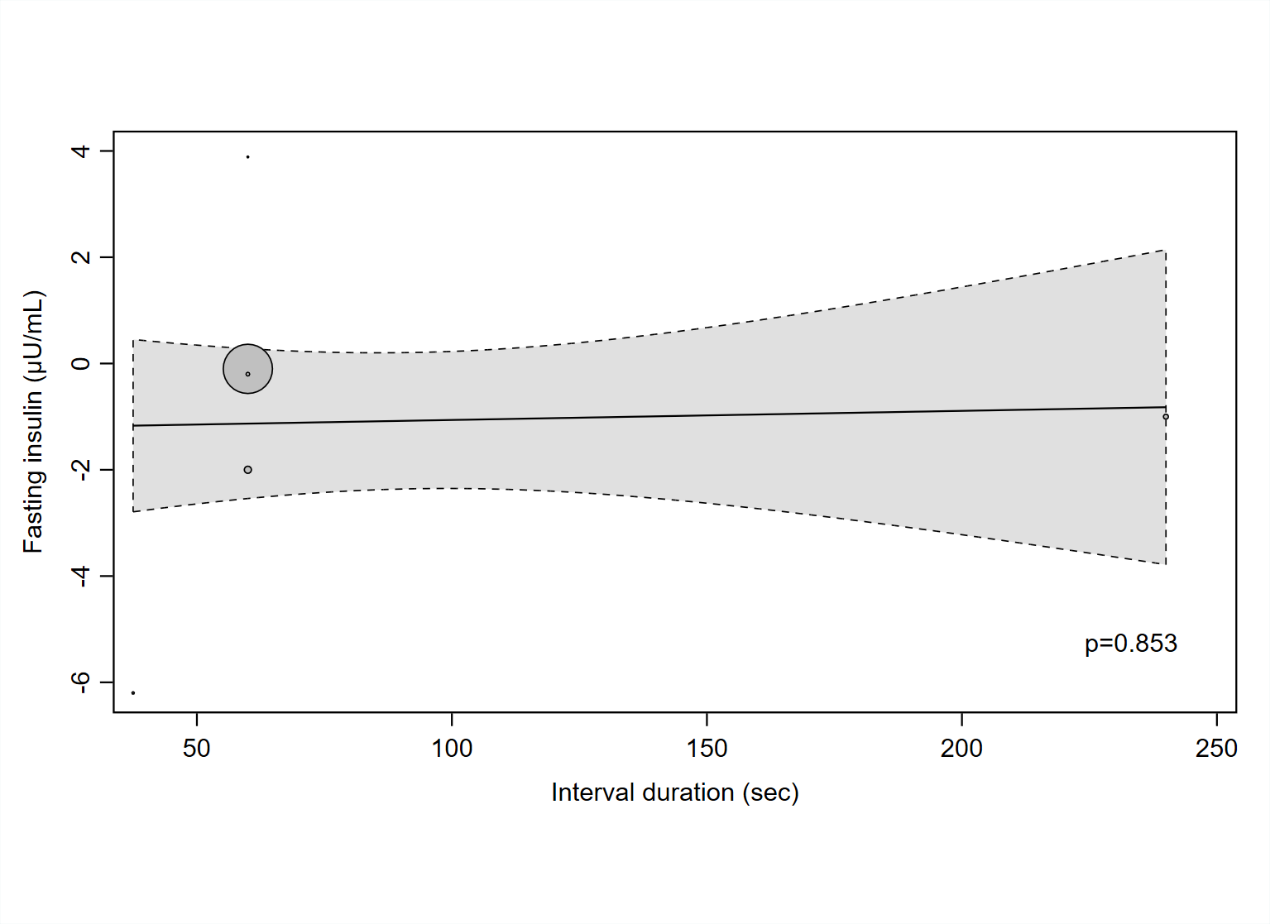

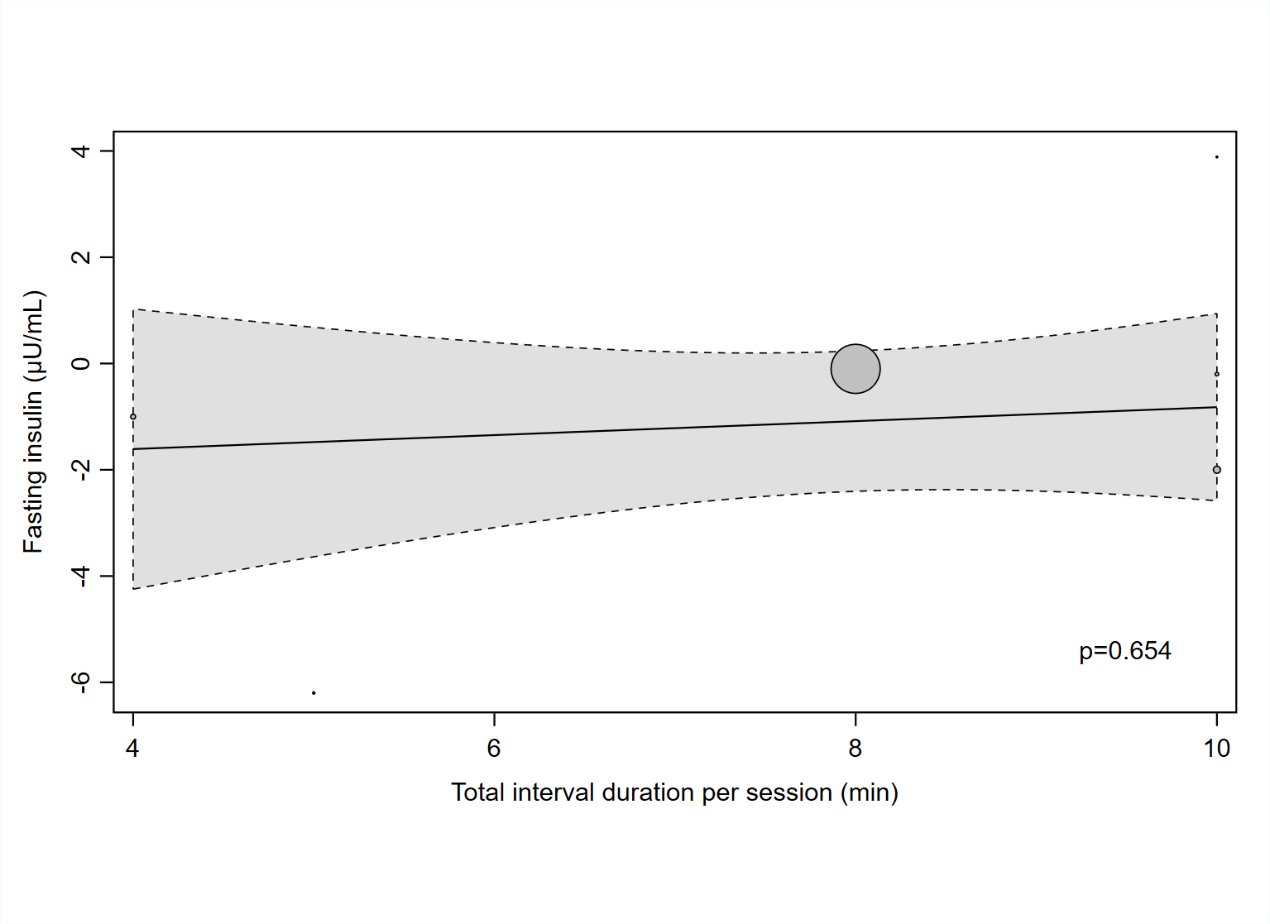

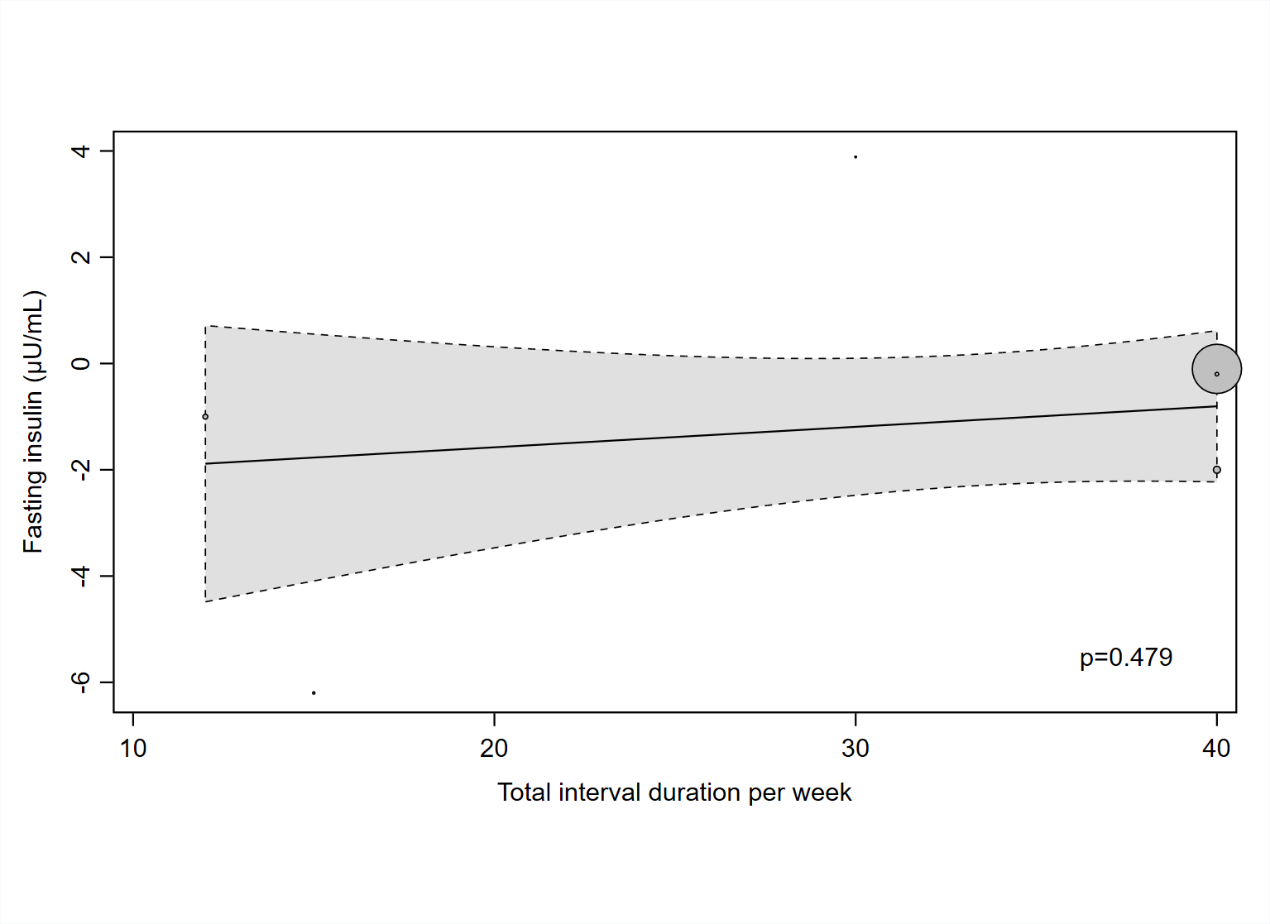

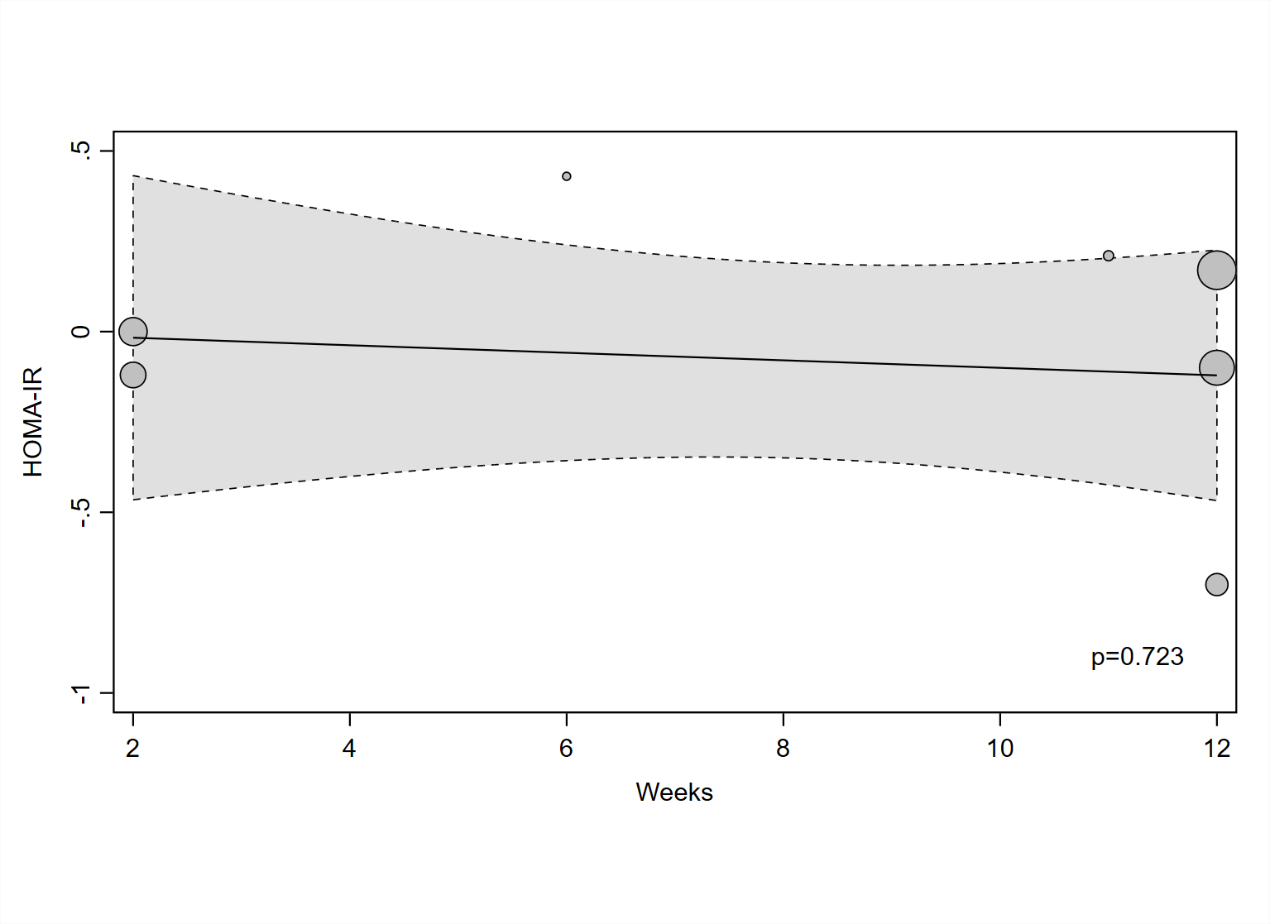

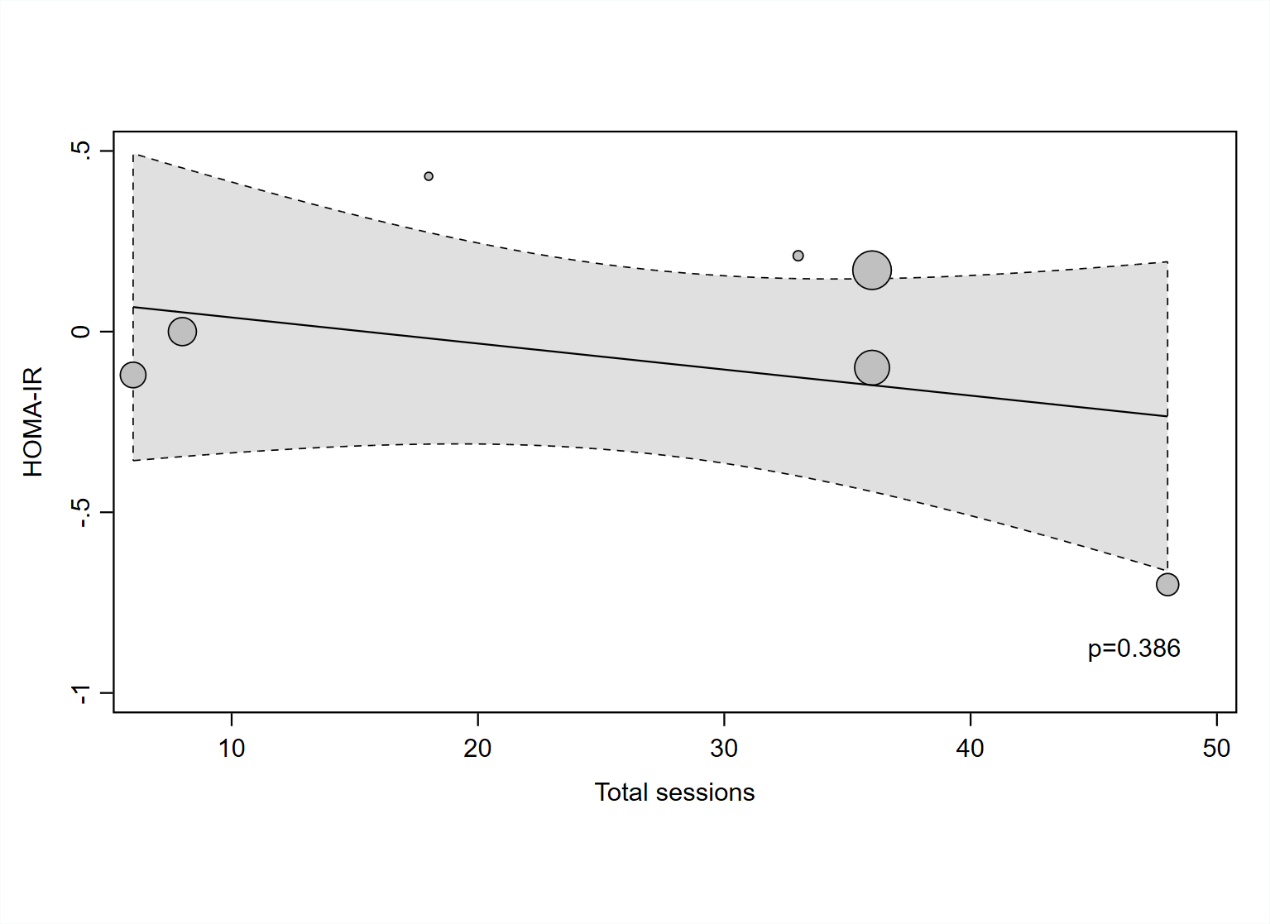

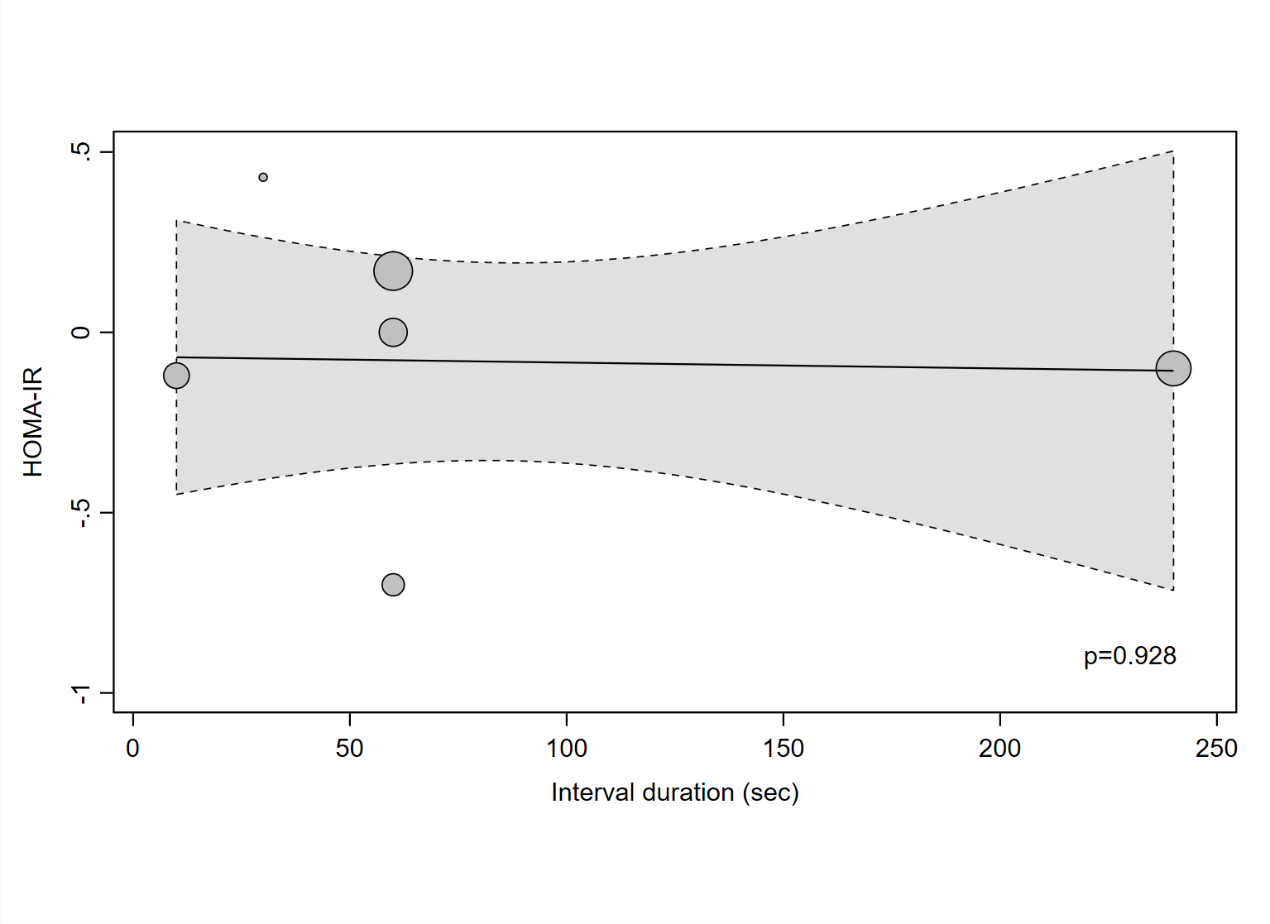

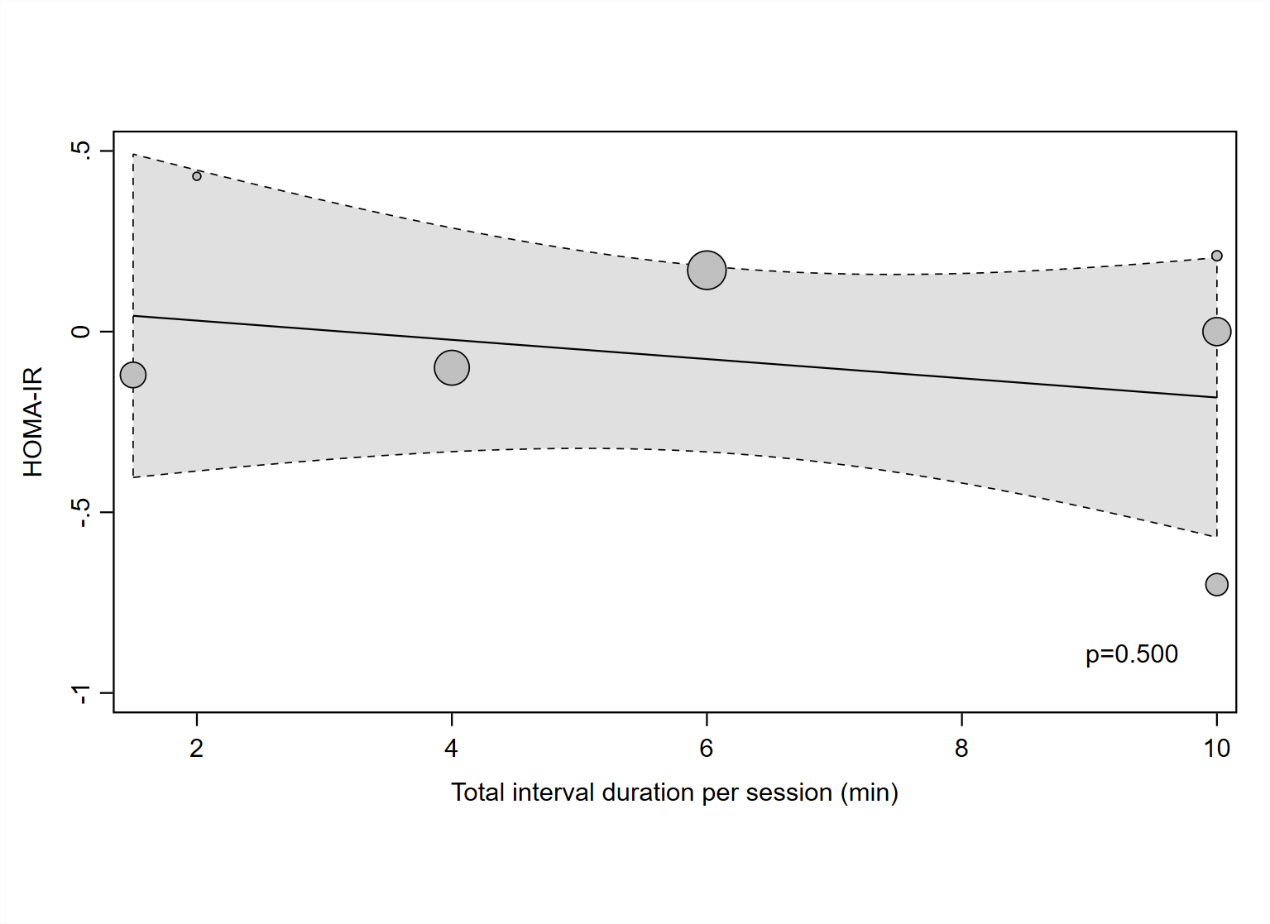

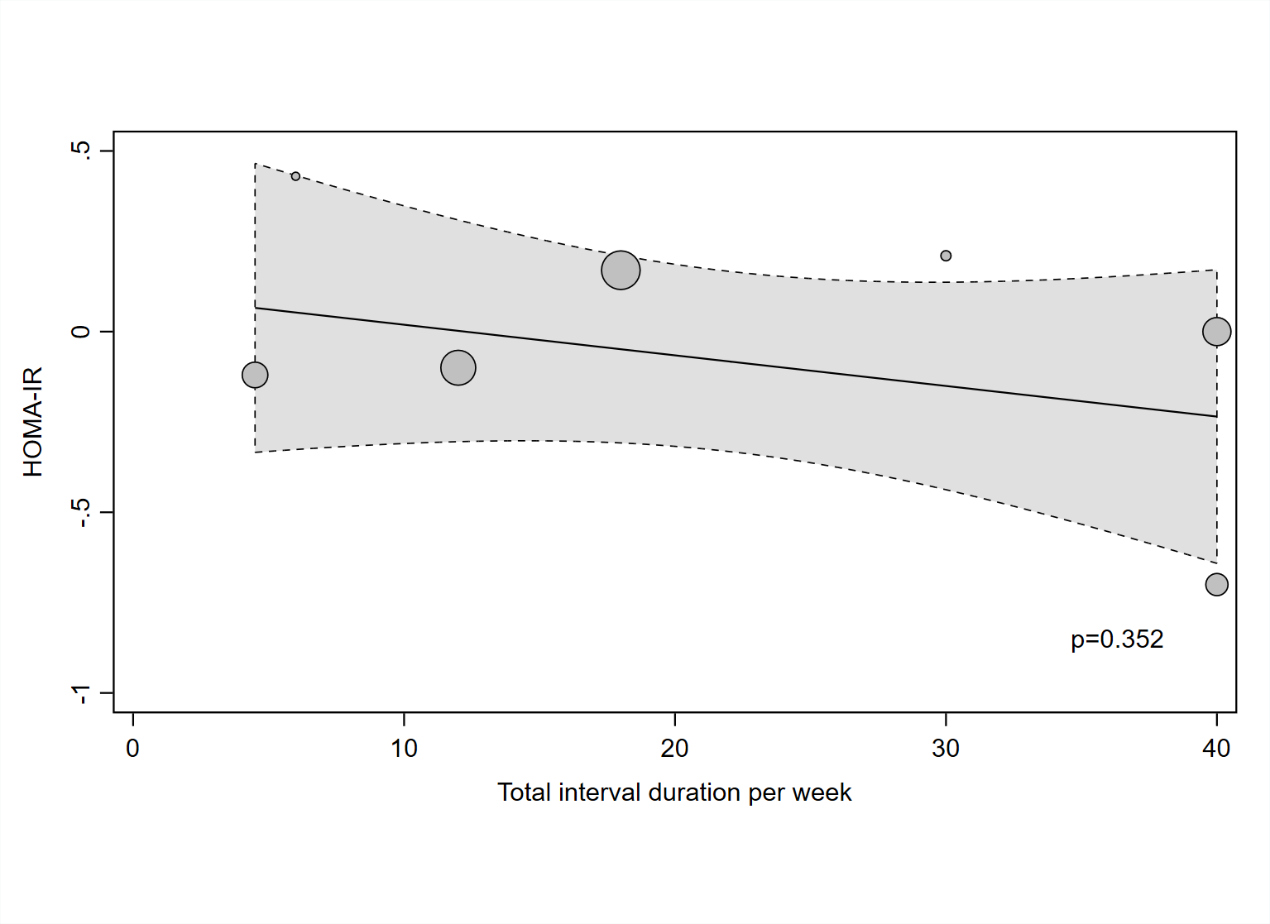

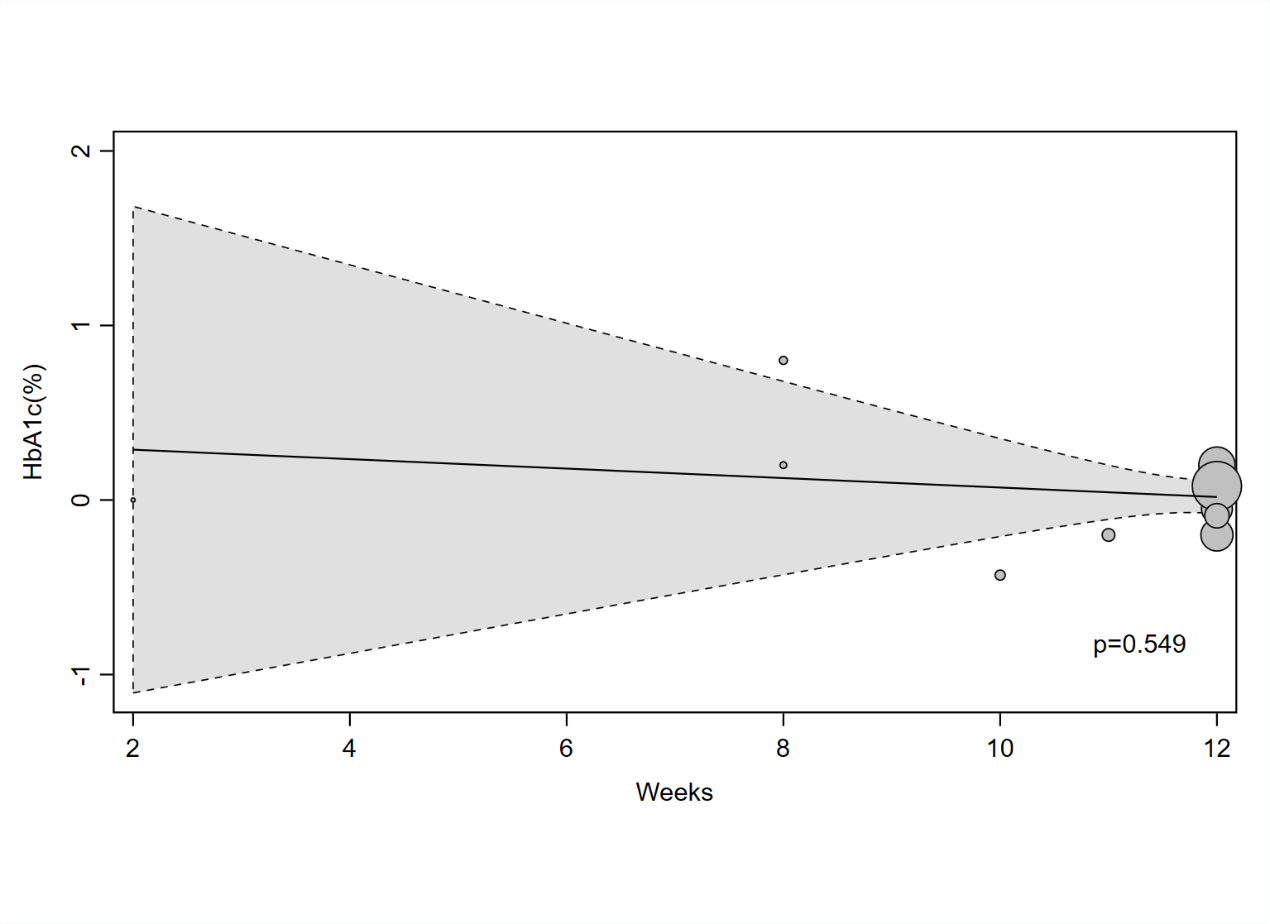

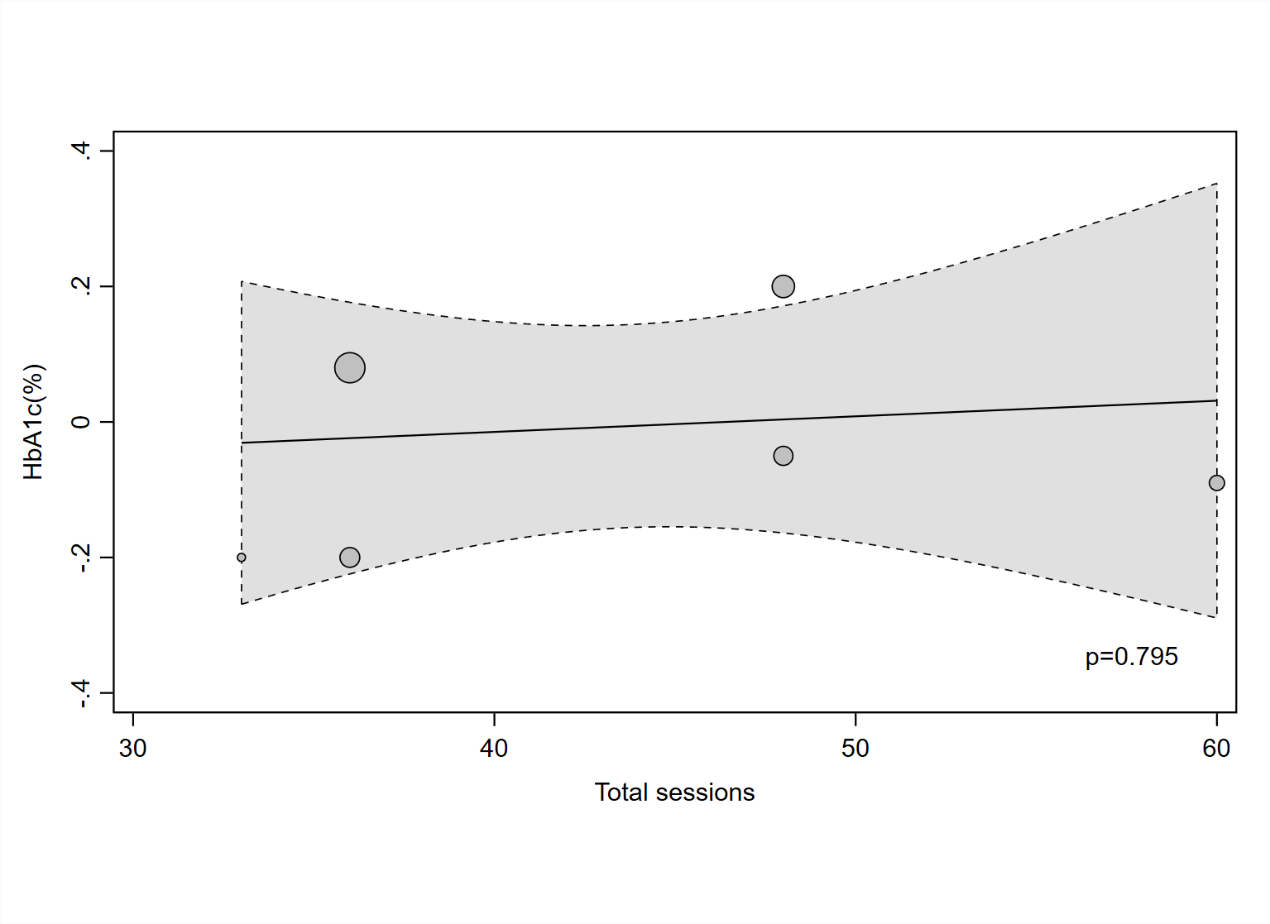

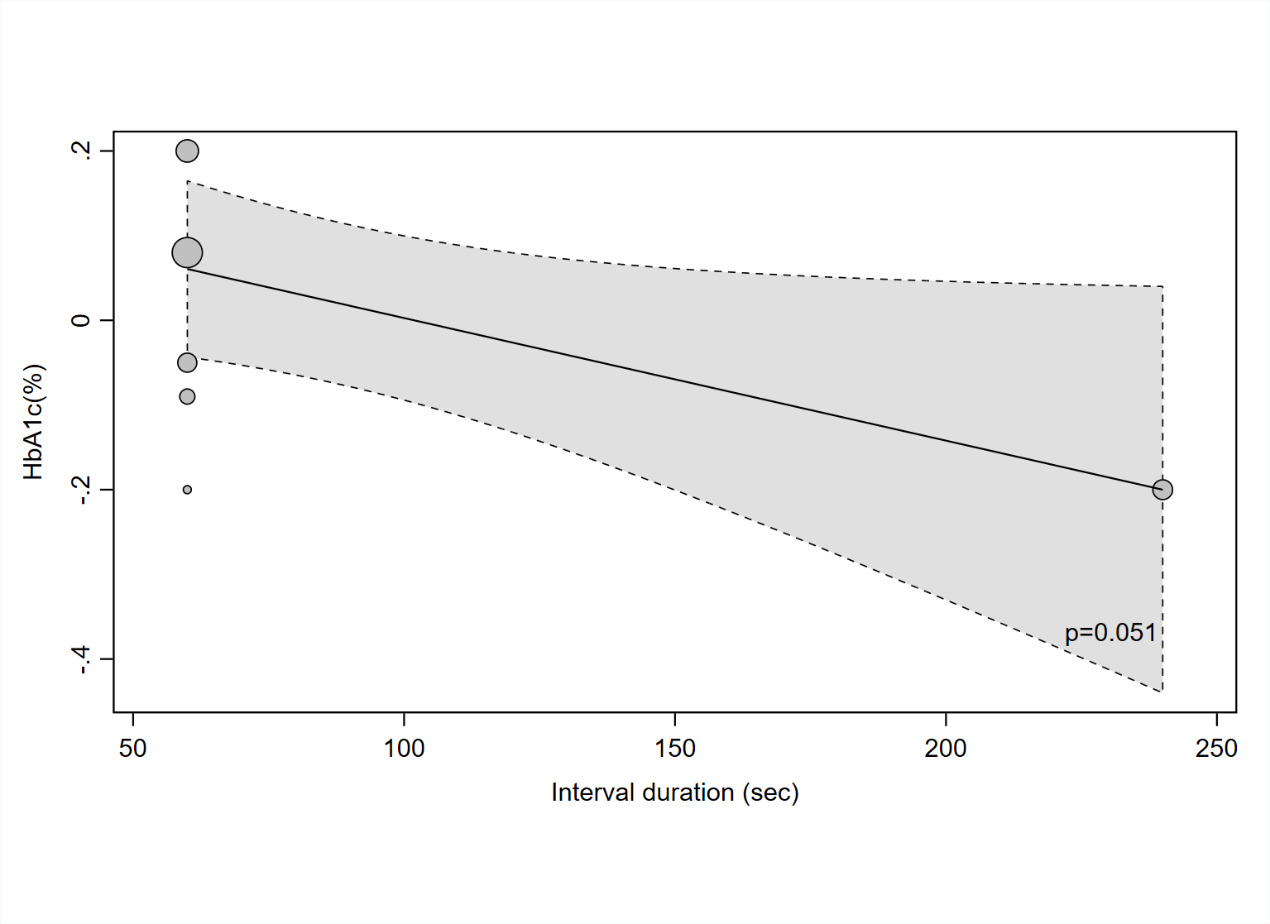

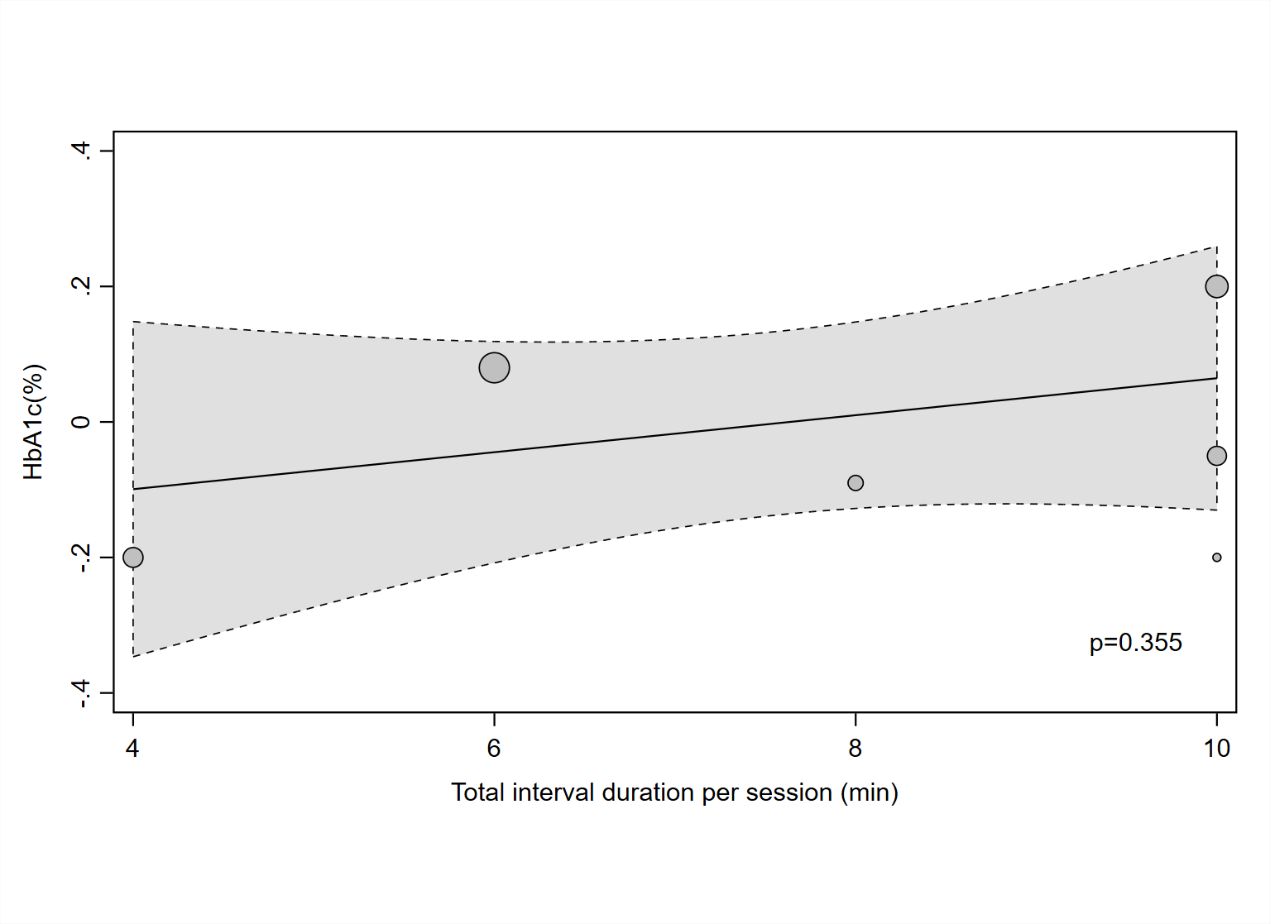

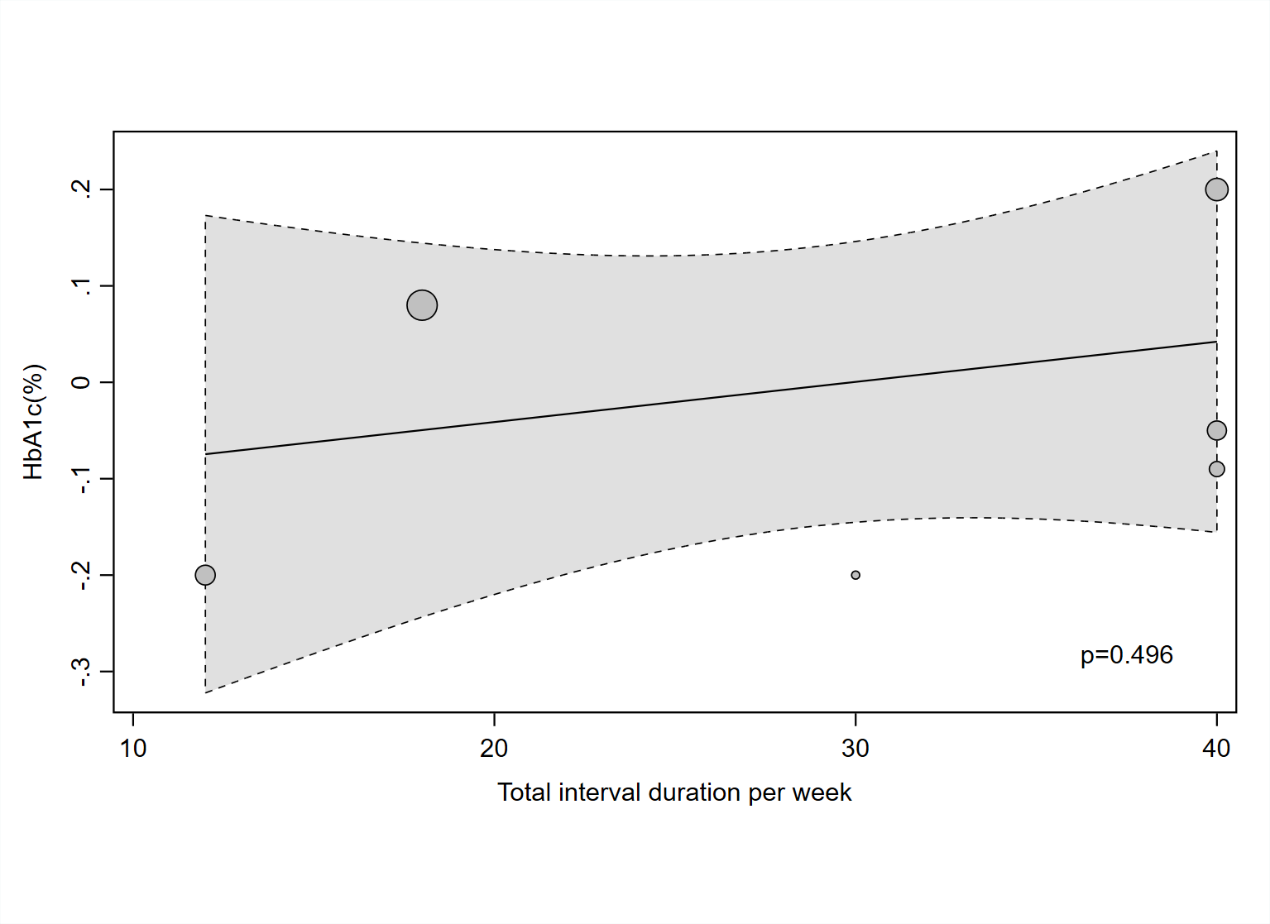


**Supplementary Table S1: Details of the search strategy**

| **Database** |  | **PubMed (n=355)** |
| --- | --- | --- |
| Date |  | January 1, 2000-January 31, 2023 |
| Search Terms | #1 | “high-intensity interval training”[MeSH] OR “high-intensity interval training”[TIAB] OR “sprint interval training”[TIAB] OR “interval training”[TIAB] OR “intermittent training”[TIAB] OR “interval exercise”[TIAB] OR “intermittent exercise”[TIAB] OR “HIIT”[All Fields] OR “HIIE”[All Fields] OR “SIT”[All Fields] Filters: Randomized Controlled Trial, Adult: 19+ years |
|  | #2 | “insulin resistance”[MeSH] OR “insulin”[MeSH] OR “insulin” [All Fields] OR “hba1c”[All Fields] OR “glucose”[All Fields] OR “HOMA-IR”[All Fields] Filters: Randomized Controlled Trial, Adult: 19+ years |
| Search Performed |  | #1 AND #2 |
| **Database** |  | **SCOPUS (N=1619)** |
| Date |  | January 1, 2000-January 31, 2023 |
|  | #1 | TITLE-ABS-KEY (“high-intensity interval training” OR “sprint interval training” OR “interval training” OR “intermittent training” OR “interval exercise” OR “intermittent exercise” OR “HIIT” OR “HIIE” OR “SIT” |
|  | #2 | “insulin resistance” OR “insulin” OR “hba1c” OR “glucose” OR “HOMA-IR” |
|  | #3 | (child*) OR (pregnan*) OR (disable*) OR (athlete) |
| Search Performed |  | #1 AND #2 AND NOT #3 |
| **Database** |  | **Web of Science (n=1877)** |
| Date |  | January 1, 2000-January 31, 2023 |
|  | #1 | TS=(“high-intensity interval training” OR “sprint interval training” OR “interval training” OR “intermittent training” OR “interval exercise” OR “intermittent exercise” OR “HIIT” OR “HIIE” OR “SIT”) |
|  | #2 | TS=(“insulin resistance” OR “insulin” OR “hba1c” OR “glucose” OR “HOMA-IR”) |
| Search Performed |  | #1) AND #2) NOT TS=((child*) OR (old*) OR (eld*) OR (pregnan*) OR (disable*) OR (athlete)) |
| **Database** |  | **The Cochrane library (n=2010)** |
| Date |  | January 1, 2000-January 31, 2023 |
|  | #1 | MeSH descriptor: [High-intensity interval training] this term only |
|  | #2 | “sprint interval training” OR “interval training” OR “intermittent training” OR “interval exercise” OR “intermittent exercise” OR “HIIT” OR “HIIE” OR “SIT” |
|  | #3 | #1 OR #2 |
|  | #4 | “insulin resistance” OR “insulin” OR “hba1c” OR “glucose” OR “HOMA-IR” |
|  | #5 | #1 OR #2 AND #4 |
|  | #6 | (child*) OR (old*) OR (eld*) OR (pregnan*) OR (disable*) OR (athlete) |
| Search Performed | #7 | #1 OR #2 AND #4 NOT #6 |

**Supplementary Table S2: Details of the study quality and risk of bias**
